# Comparative and dose-response effects of supplementary exercise training on VO_2_max in trained athletes: a Systematic Review and Bayesian network meta-analysis

**DOI:** 10.3389/fphys.2026.1906583

**Published:** 2026-07-30

**Authors:** Dongze Li, Fanli Zhou, Xinshu Li, Kong Shibo, Jie Wang, Xinmiao Feng, Jiezhong Wu

**Affiliations:** 1 School of Sports Science and Engineering, East China University of Science and Technology, Shanghai, China; 2 Jiangsu Normal University, School of Physical Education, Jiangsu, China; 3 School of Education, Beijing Sport University, Beijing, China; 4 School of Physical Education, Shanghai University of Sport, Shanghai, Shanghai, China; 5 College of Physical Education, Henan Agricultural University, Henan, China; 6 China Wushu School, Beijing Sport University, Haidian, Beijing, China; 7 Department of physical education, Fuzhou University, Minhou, Fuzhou, China

**Keywords:** athletes, dose-response, exercise training, maximal oxygen uptake, network meta-analysis, VO2max

## Abstract

**Background:**

Maximal oxygen uptake (VO_2_max) is a critical determinant of endurance performance in athletes, yet the comparative effectiveness and dose-response profiles of different supplementary exercise training modalities remain incompletely characterized. This systematic review and Bayesian network meta-analysis aimed to compare 11 exercise training modalities for improving VO_2_max in healthy athletes, examine dose-response relationships, explore effect modification by sport type, and evaluate study-level moderators using network meta-regression.

**Methods:**

Four databases were searched for randomized controlled trials. Bayesian network meta-analysis (NMA), Bayesian network meta-regression, and dose-response NMA were conducted. Confidence in the evidence was assessed using the Confidence in Network Meta-Analysis framework.

**Results:**

Sixty-one studies were included in the systematic review; 60 contributed to the main NMA and 31 to the dose-response NMA. Five exercise modalities were significantly superior to the active control condition, which consisted of maintaining regular sport-specific training without additional supplementary intervention: blood flow restriction training, high-intensity interval training (HIIT), intermittent hypoxic training, sprint interval training (SIT), and small-sided games (SSG). HIIT and SIT showed the most consistent overall support, although sparse subgroup findings remained exploratory. The dose-response analysis suggested that the modelled significant regions extended across the observed supplementary exercise dose ranges for HIIT, SIT, and SSG. Bayesian network meta-regression showed no clear effect modification by age, baseline VO_2_max, or intervention duration.

**Conclusions:**

Given the predominantly low to very low certainty of evidence, the findings should be interpreted cautiously. HIIT and SIT appeared to be the most consistently supported supplementary modalities for improving VO_2_max in trained athletes, but these model-based findings require confirmation in larger, higher-quality head-to-head trials with common active control groups and standardized VO_2_max methodology.

**Systematic Review Registration:**

https://www.crd.york.ac.uk/PROSPERO/view/CRD420261360986, identifier CRD420261360986.

## 1 Introduction

Maximal oxygen uptake (VO_2_max) is widely recognized as a primary determinant of aerobic endurance performance and a standard measure of cardiorespiratory fitness (Bassett and Howley, 2000). Defined as the maximum rate at which oxygen can be taken up, transported, and utilized by working skeletal muscle during incremental exercise, VO_2_max reflects the integrated capacity of the pulmonary, cardiovascular, and muscular systems (Joyner and Coyle, 2008). In athletic populations, VO_2_max values range from approximately 40 mL/kg/min in skill-based sport athletes to more than 85 mL/kg/min in elite endurance athletes, and this variation explains a substantial proportion of inter-individual differences in endurance performance (di Prampero, 2003; Levine, 2008). Consequently, identifying evidence-based strategies to maximize VO_2_max gains through supplementary training is a question of practical importance for athletes, coaches, and sport scientists.

The literature on exercise training for VO_2_max improvement spans a diverse array of modalities including moderate-intensity continuous training (MICT), high-intensity interval training (HIIT), sprint interval training (SIT), resistance training (RT), small-sided games (SSG), blood flow restriction training (BFR), intermittent hypoxic training (IHT), polarized training (POL), high-volume training (HVT), threshold training (THR), and aerobic exercise (AE) (Seiler, 2010; Garber et al., 2011; Salanti et al., 2011; Gibala et al., 2012; Buchheit and Laursen, 2013a; Buchheit and Laursen, 2013b; Stöggl and Sperlich, 2014; Milanović et al., 2015; MacInnis and Gibala, 2017; Wen et al., 2019; Clemente et al., 2021; Yang et al., 2021; Zhang et al., 2025). Each modality is characterized by distinct combinations of intensity, duration, and physiological target. Several pairwise meta-analyses have compared specific pairs, most frequently HIIT versus MICT (Milanović et al., 2015) or polarized versus threshold training (Rosenblat et al., 2019), and have generally reported that higher-intensity approaches produce larger VO_2_max gains. But pairwise meta-analyses answer only narrow questions: they can synthesize evidence from studies that directly compared two specific interventions, yet cannot tell an athlete which of eleven available options is most worth their limited training time. Mechanistically, these modalities may not differ only in training format, but also in the physiological signals they impose. Higher-intensity interval and sprint-based exercise may place stronger demands on central oxygen delivery, stroke volume, and peripheral oxidative adaptation than additional moderate-intensity work, whereas BFR and IHT may introduce metabolic or hypoxic stimuli that are not fully captured by conventional external-volume descriptors (Bassett and Howley, 2000; di Prampero, 2003; Joyner and Coyle, 2008; Levine, 2008; Gibala et al., 2012; Buchheit and Laursen, 2013a; Buchheit and Laursen, 2013b; MacInnis and Gibala, 2017).

Network meta-analysis (NMA) solves this problem by pooling direct and indirect evidence within a single model, enabling the simultaneous comparison and probabilistic ranking of multiple interventions, including pairs that have never been tested head to head (Salanti, 2012; Hutton et al., 2015). In the exercise science domain, recent NMA has been used to compare training intensity distribution interventions in endurance athletes (Rosenblat et al., 2025) and interval training methods across athletic populations (Yang et al., 2025). These studies show that the approach can be applied in this field, but they were still limited in scope, covering only a handful of modalities or interval subtypes. No NMA has yet compared the full menu of training options available to athletes.

A second gap is dose. The size of a VO_2_max gain depends on the standardized supplementary exercise dose, and session duration, intensity, and frequency all contribute to that exposure (Wasfy and Baggish, 2016). Conventional meta-analyses treat training as a yes or no category, ignoring the continuous relationship between dose and response. The MBNMAdose framework (Mbnmadose-Package: Mbnmadose for Dose-Response Model-Based Network Meta-Analysis in Mbnmadose: Dose-Response MBNMA Models) makes it possible to model comparative effectiveness and dose-response simultaneously. This approach has already been used in exercise dose-response analyses for blood pressure in metabolic syndrome and after stroke or transient ischemic attack (Zhang et al., 2026b; Zhang et al., 2026a), but it has not been applied to athletes, where the supplementary dose sits on top of a substantial baseline. Importantly, in athletic populations, control conditions typically involve maintenance of regular sport-specific training rather than complete physical inactivity, making further improvements in VO_2_max more difficult to achieve.

Athletes from different sports also bring different baselines to the table. A cyclist, a volleyball player, and a swimmer each maintain sport-specific training that differs in intensity distribution, energy system demands, and total volume (Seiler, 2010; Stöggl and Sperlich, 2014). Whether supplementary training works differently depending on what sport an athlete comes from is a question no large-scale NMA has addressed. Answering it is essential for moving from generic recommendations to ones that fit the athlete in front of you.

The present study set out to do four things: (1) run a comprehensive Bayesian NMA ranking 11 training modalities on VO_2_max improvement in healthy athletes, with robustness tested through two prespecified sensitivity analyses and formal inconsistency assessment; (2) model the dose-response relationship between supplementary training volume (METs-min/week) and VO_2_max gain for each modality using MBNMAdose; (3) test whether comparative effectiveness differs by sport type through prespecified subgroup analyses; and (4) explore study-level moderators using Bayesian network meta-regression for age, baseline VO_2_max, and intervention duration.

## 2 Methods

### 2.1 Protocol and registration

This systematic review and network meta-analysis was registered with PROSPERO CRD420261360986 and conducted in accordance with the PRISMA extension for network meta-analysis guidelines (Hutton et al., 2015).

### 2.2 Search strategy and selection

A systematic search was performed on 3 April 2026 in PubMed, Web of Science, Embase, and the Cochrane Central Register of Controlled Trials, with no language restrictions. The search strategy combined controlled vocabulary (MeSH, Emtree) and free-text keywords across four domains linked by AND: population (athletes), intervention (exercise training modalities), outcome (maximal oxygen uptake), and study design (randomized controlled trials). Full search strategies for each database are documented in **Supplementary Appendix 1**. Backward citation tracking and forward citation searching were also conducted. After removing duplicates, two reviewers independently screened titles and abstracts. Potentially eligible records underwent full-text assessment against the eligibility criteria. Disagreements were resolved through discussion with a third reviewer.

### 2.3 Eligibility criteria

Eligibility criteria were defined using the PICOS framework and applied independently by two reviewers. (1) Population: healthy athletes engaged in regular, structured sport-specific training at any competitive level. Athlete status was verified from the original study descriptions, including explicit identification as athletes or players, participation in organized sport-specific training, membership of a sport team or club, or reported involvement in competitive or structured training programs. Studies in which participants were described only as physically active, recreationally active, or untrained were excluded unless regular sport-specific training was clearly documented. (2) Intervention: any structured exercise training modality performed in addition to regular sport-specific training, lasting at least 4 weeks. The 4-week minimum was chosen to focus on training-induced adaptations rather than acute or short-term familiarization effects, while retaining short mesocycle interventions that are common in athlete training studies. (3) Comparison: control conditions were defined as maintenance of regular sport-specific training without additional structured supplementary exercise intervention (CG), or active treatment arms in head-to-head comparisons. (4) Outcome: change in VO_2_max (mL/kg/min) from baseline to post-intervention, assessed via direct gas analysis during a maximal graded exercise test. Studies reporting estimated or predicted VO_2_max were excluded. (5) Study design: randomized controlled trials only.

### 2.4 Data extraction

Two reviewers independently extracted data using a standardized form: study characteristics, participant characteristics (sport type, sex, age, baseline VO_2_max), intervention details (modality, session duration, weekly frequency, intensity descriptors, intervention length), and outcome data (mean change in VO_2_max, SD, n per arm). When SD of the change score was not directly reported, it was estimated from confidence intervals, standard errors, p-values, or imputed using a pre-post correlation coefficient of 0.5 (Higgins et al., 2019). Multi-arm trials and duplicate arms of the same modality were handled at the study level before analysis; where multiple arms of the same modality existed within a study, arms were pooled using a sample-size weighted average of means, and a pooled SD was calculated from the combined variance. Standard errors were derived as $\text{SE}=\frac{\text{SD}}{\sqrt{\text{N}}}$ (Higgins et al., 2019). Sensitivity analyses using alternative pre-post correlations were not performed and are acknowledged as a limitation.

### 2.5 Risk of bias

Risk of bias was assessed using the revised Cochrane Risk of Bias tool (RoB 2) (Sterne et al., 2019). Each study was evaluated across five domains. Two reviewers performed assessments independently, with disagreements resolved through consensus. Domain-level judgements for each study and the overall summary across domains are presented in **Supplementary Figure 3** and **S4**.

### 2.6 Bayesian network meta-analysis

A Bayesian random-effects NMA was conducted using the gemtc package (v1.0-2) in R (v4.5.2) (van Valkenhoef et al., 2012), interfacing with JAGS (v4.3.1). The model used a normal likelihood, identity link, consistency model, and random treatment effects. CG, maintaining regular sport-specific training, served as the reference. Four MCMC chains were run with 5,000 adaptation iterations and 20,000 sampling iterations, thinned by 10 (8,000 posterior samples). Convergence was assessed using the Gelman-Rubin diagnostic (R-hat < 1.05) (Gelman and Rubin, 1992) and visual inspection of trace plots (**Supplementary Figure 7**). Treatment rankings used SUCRA (Salanti et al., 2011). Inconsistency was assessed via: (1) DIC comparison of consistency vs UME models, with a difference below 5 considered acceptable (Spiegelhalter et al., 2002); (2) node splitting (mtc.nodesplit) for comparisons with both direct and indirect evidence, with p < 0.05 indicating inconsistency (Dias et al., 2010) (**Supplementary Tables 6**-**S7** and **Supplementary Figure 8**); and (3) within-design heterogeneity (Q, I²). Transitivity was assessed descriptively before interpreting network estimates by comparing the distribution of plausible effect modifiers across treatment nodes, including sport type, age, sex, baseline VO_2_max, intervention duration, competitive level when reported, and the nature of the control condition. This assessment was qualitative because individual participant data and detailed background training-load data for control arms were not consistently available. The transitivity assessment is summarized in **Supplementary Table 20**. Prespecified Bayesian network meta-regression models were also fit separately for age, baseline VO_2_max, and intervention duration. Models used the default vague priors in gemtc: relative treatment effects were assigned vague normal priors and the between-study SD was assigned the default uniform prior on the outcome scale. No separate prior-sensitivity analysis was performed, and this is acknowledged as a limitation.

### 2.7 Sensitivity and subgroup analyses

Two pre-specified sensitivity analyses were conducted: Sensitivity 1 excluded six high risk-of-bias studies (n=54); Sensitivity 2 excluded ten IHT studies performed under hypoxic/altitude conditions (n = 50). A planned split of IHT into normoxic-IHT and hypoxic-IHT was considered but could not be implemented reliably because the resulting network was too sparse for stable estimation; the transitivity concern is therefore addressed through Sensitivity 2 and in the limitations. For subgroup analyses, studies were classified by sport type: endurance sports (25 studies), team ball sports (18), water sports (7), and combat and racket sports (10). These categories were retained to preserve consistency with the prespecified analyses and supplementary results, but the sparse non-endurance networks mean that subgroup findings should be treated as exploratory. The endurance subgroup received a full Bayesian NMA. The remaining three subgroups had insufficient network connectivity for reliable NMA; pairwise random-effects meta-analyses (DerSimonian-Laird) were performed for each treatment with direct CG comparisons (DerSimonian and Laird, 1986). These subgroup pairwise analyses were exploratory checks only, and their frequentist p values should not be directly compared with Bayesian CrIs from the primary NMA.

### 2.8 Dose-response analysis

Thirty-one studies with quantifiable supplementary dose data entered the dose-response NMA. MET-min/week was used as a pragmatic common external-dose metric to quantify weekly supplementary training exposure across interventions differing in duration, frequency, and reported intensity. This metric was not assumed to represent equivalent internal physiological load, recovery cost, or adaptive stimulus across modalities, particularly for supramaximal or resistance-based exercise. Accordingly, modality-level MET anchors, including those for SIT and HIIT, were used as standardized approximations for external-dose harmonization rather than empirically measured internal-load values for each trial. Weekly dose (METs-min/week) was calculated as MET value × session duration × weekly frequency×average adherence rate, representing net supplementary training beyond regular sport-specific practice. MET values were assigned via a two-tier strategy: direct matching to the 2024 Adult Compendium of Physical Activities (Herrmann et al., 2024) for studies reporting absolute workload, and standardized modality-level anchoring for studies reporting relative intensity (SIT = 15.0 METs (Gibala et al., 2012); HIIT = 12.0 (Buchheit and Laursen, 2013a); AE = 5.5 (Seiler, 2010); SSG = 9.5 (Hill-Haas et al., 2011); MICT = 8.0 (Seiler, 2010); RT = 6.0 (American College of Sports Medicine, 2009); CG = 0; see **Supplementary Table 13**). The resulting study-arm-level dose values used in the dose-response model are provided in **Supplementary Table 19**. The dose- and agent-level network connectivity was verified prior to modelling (**Supplementary Figure 14**-**S15**). Eight dose-response models were compared via DIC using the MBNMAdose package (Lu and Ades, 2004). The quadratic function model had the lowest DIC and was selected for the primary dose-response analysis. An effect was considered significant when the full 95% CrI exceeded zero. A note on SIT dose limitations is in **Supplementary Appendix 14.2**.

### 2.9 Publication bias and evidence quality

Publication bias was assessed via comparison-adjusted funnel plots and Egger’s regression test (Egger et al., 1997). Confidence in evidence was evaluated using the CINeMA framework (Nikolakopoulou et al., 2020) across six domains, with each comparison rated as high, moderate, low, or very low confidence; the network plot of study limitations contributing to the CINeMA appraisal is provided in **Supplementary Figure 13**.

## 3 Results

### 3.1 Study selection

The database search yielded 4,310 records from electronic databases (PubMed: 1,171; Web of Science: 723; Embase: 1,749; Cochrane: 667). After removing 1,182 duplicates, 3,128 records were screened by title and abstract, and 2,937 were excluded. A total of 191 full-text reports from the database search were assessed for eligibility; 132 were excluded for not meeting the PICOS criteria, leaving 59 eligible studies from the database-search pathway. In parallel, 13 records were identified from previous reviews and citation tracking, of which 2 met the eligibility criteria and were added. Overall, 61 studies were included in the systematic review; 60 contributed to the main Bayesian NMA and 31 provided sufficient quantifiable supplementary dose data for the dose-response NMA (**Figure 3**).

### 3.2 Study characteristics

The 11 modalities evaluated were AE, BFR, HIIT, HVT, IHT, MICT, POL, RT, SIT, SSG, and THR (definitions: **Supplementary Table 1**). Athletes came from 25 sport disciplines across four categories: endurance sports (25 studies, 41.7%), team ball sports (18, 30.0%), combat and racket sports (10, 16.7%), and water sports (7, 11.7%). Detailed study characteristics are provided in **Supplementary Table 2** and **S3**.

Participant age ranged from 13.5 to 47.2 years, baseline VO_2_max from 35.25 to 81.00 mL/kg/min, and intervention duration from 4 to 25 weeks. Across the main NMA dataset, 1,377 participants were represented at the study-arm level: 823 in male-only arms (59.8%), 84 in female-only arms (6.1%), 398 in mixed-sex arms (28.9%), and 72 in arms where sex was not reported (5.2%). Only three studies included female-only samples. Competitive level was extracted when explicitly reported, but terminology was inconsistent across trials; therefore, it was summarized descriptively rather than modelled as a formal moderator. Treatment-node-level distributions of potential effect modifiers were not fully balanced across modalities and are summarized in **Supplementary Table 20**.

### 3.3 Risk of bias

Of 61 studies, 36 (59.0%) had low risk of bias, 19 (31.1%) raised some concerns, and 6 (9.8%) had high risk (study-level domain judgements: **Supplementary Figure 3**; cross-domain summary: **Supplementary Figure 4**). Domain 2 (deviations from intended interventions) was the most frequent concern, unsurprising given that blinding participants to exercise modality is impossible. All 61 studies were low risk on Domain 4 (outcome measurement), reflecting standardized gas analysis for VO_2_max. The six high-risk studies were excluded in Sensitivity 1.

### 3.4 Network geometry and inconsistency

The network comprised 11 nodes connected by 36 unique pairwise comparisons across 14 study designs (**Supplementary Figure 1**; geometry summary in **Supplementary Table 4**). Global DIC comparison (consistency model: 99.37; UME: 98.39; difference: −0.98) showed acceptable global consistency. Node splitting evaluated 19 comparisons; 17 (89.5%) were consistent (all p > 0.05) (**Supplementary Tables 6**-**S7**; visualized in **Supplementary Figure 8**). Two comparisons involving IHT showed evidence of inconsistency: AE vs IHT (p = 0.023) and HIIT vs IHT (p = 0.050, at the conventional threshold). The HIIT and IHT comparison is more precisely described as showing marginal inconsistency given the borderline p value. In both cases, indirect evidence suggested larger effects than direct evidence, likely reflecting the challenge of pooling normoxic and hypoxic training conditions under a common transitivity assumption. All MCMC chains converged (R-hat range: 1.000 to 1.004) (**Supplementary Figure 7**).

### 3.5 Primary NMA results

**Figure 1:**
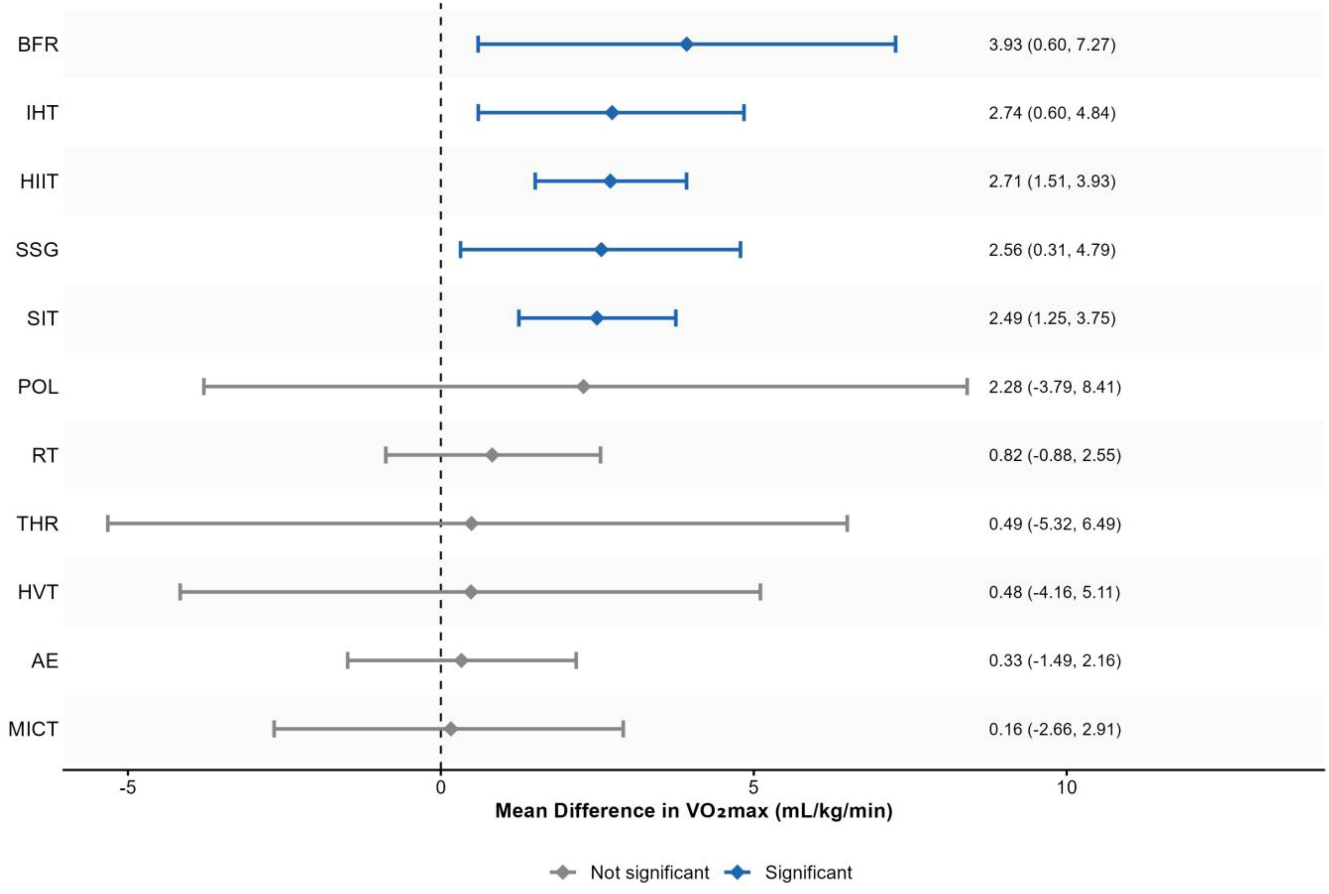
Forest plot.

**Figure 2:**
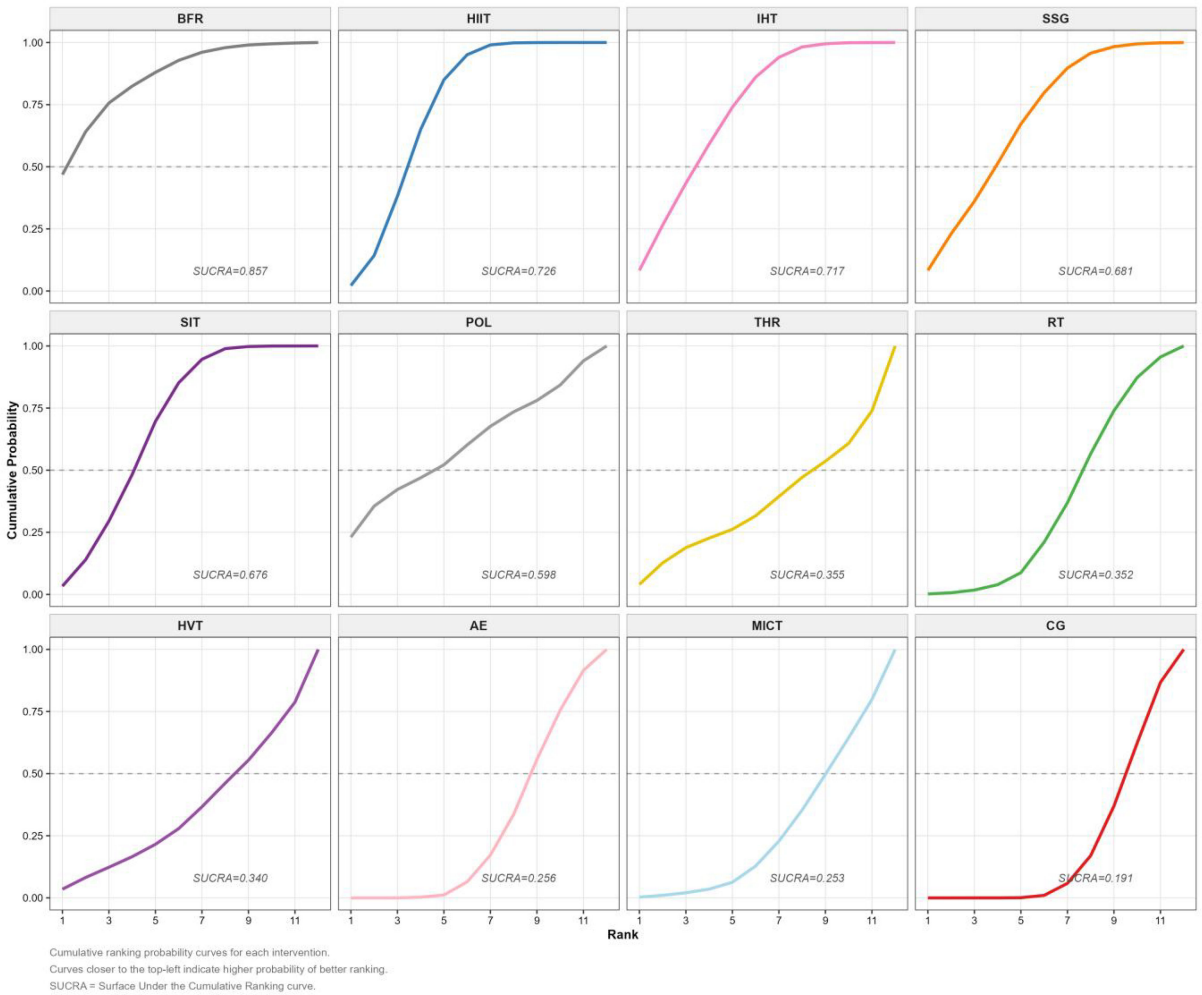
SUCRA ranking probabilities for the 11 exercise modalities and the control condition on VO_2_max.

All 11 modalities had positive point estimates versus CG, although only five had 95% CrIs excluding zero (**Table 1**; **Figure 1**; pairwise league table visualized in **Supplementary Figure 6**). These five modalities were BFR (3.93 mL/kg/min [0.60, 7.27]), IHT (2.74 [0.60, 4.84]), HIIT (2.71 [1.51, 3.93]), SSG (2.56 [0.31, 4.79]), and SIT (2.49 [1.25, 3.75]). HIIT and SIT had the narrowest credible intervals among significant treatments. The remaining six, POL (2.28 [−3.79, 8.41]), RT (0.82 [−0.88, 2.55]), HVT (0.48 [−4.16, 5.11]), THR (0.49 [−5.32, 6.49]), AE (0.33 [−1.49, 2.16]), and MICT (0.16 [−2.66, 2.91]), did not reach significance. SUCRA identified BFR as having the highest probability of being best (0.857), followed by HIIT (0.726), IHT (0.717), SSG (0.681), and SIT (0.676). CG ranked last (0.191) (SUCRA bar chart: **Figure 2**). SUCRA reflects ranking probability, not certainty: BFR’s top rank coexists with its widest credible interval. Tau was 1.22 (I² = 34.5%).

**Table 1:** Treatment effects vs CG and SUCRA rankings.

Treatment	MD vs CG	95% CrI	SUCRA	Rank
BFR^a^	3.93	[0.60, 7.27]	0.857	1
HIIT^b^	2.71	[1.51, 3.93]	0.726	2
IHT^c^	2.74	[0.60, 4.84]	0.717	3
SSG^d^	2.56	[0.31, 4.79]	0.681	4
SIT^e^	2.49	[1.25, 3.75]	0.676	5
POL^f^	2.28	[-3.79, 8.41]	0.598	6
THR^g^	0.49	[-5.32, 6.49]	0.355	7
RT^h^	0.82	[-0.88, 2.55]	0.352	8
HVT^i^	0.48	[-4.16, 5.11]	0.340	9
AE^j^	0.33	[-1.49, 2.16]	0.256	10
MICT^k^	0.16	[-2.66, 2.91]	0.253	11
CG^l^	—	—	0.191	12

^a^BFR, Blood Flow Restriction Training; ^b^HIIT, High-Intensity Interval Training; ^c^IHT, Intermittent Hypoxic Training; ^d^SSG, Small-Sided Games; ^e^SIT, Sprint Interval Training; ^f^POL, Polarized Training; ^g^THR, Threshold Training; ^h^RT, Resistance Training; ^i^HVT, High-Volume Training; ^J^AE , Aerobic Exercise; ^k^MICT, Moderate-Intensity Continuous Training; ^l^CG, Control Group.

**Figure 3:**
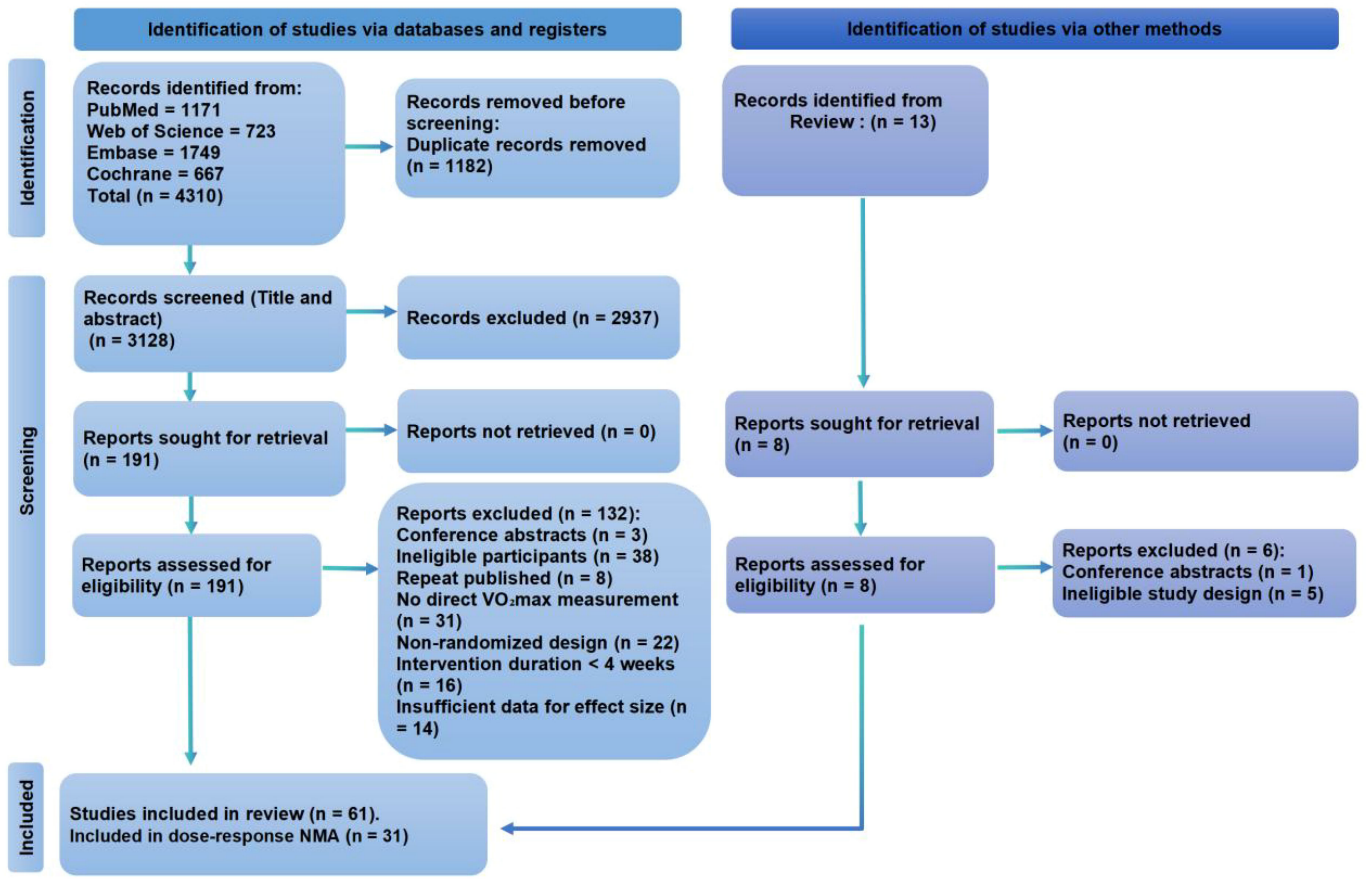
PRISMA flow diagram for included and excluded studies.

Note: BFR : Blood Flow Restriction Training;HIIT: High-Intensity Interval Training; IHT: Intermittent Hypoxic Training; SSG: Small-Sided Games; SIT: Sprint Interval Training; POL: Polarized Training; THR: Threshold Training; RT: Resistance Training;HVT: High-Volume Training;AE: Aerobic Exercise;MICT: Moderate-Intensity Continuous Training.

Note: BFR : Blood Flow Restriction Training;HIIT: High-Intensity Interval Training; IHT: Intermittent Hypoxic Training; SSG: Small-Sided Games; SIT: Sprint Interval Training; POL: Polarized Training; THR: Threshold Training; RT: Resistance Training;HVT: High-Volume Training;AE: Aerobic Exercise;MICT: Moderate-Intensity Continuous Training;CG: Control Group.

### 3.6 Sensitivity analyses

Three modalities, BFR, HIIT, and SIT, stayed significant in both sensitivity analyses (**Supplementary Figure 9**; **Supplementary Table 8**). When high-risk studies were removed (Sensitivity 1, n = 54), all five primary significant treatments held. When IHT studies were removed (Sensitivity 2, n = 50), BFR, HIIT, and SIT remained significant, but SSG’s interval widened to cross zero, suggesting that its primary-analysis significance partly depended on indirect evidence routed through IHT. This matches the node splitting finding that IHT-related comparisons showed inconsistency. IHT, absent from Sensitivity 2 by design, remained significant in Sensitivity 1.

### 3.7 Network meta-regression

Prespecified Bayesian network meta-regression models were fitted separately for age, baseline VO_2_max, and intervention duration (**Supplementary Table 9**). These exploratory study-level models did not detect clear effect modification, and all 95% CrIs crossed zero. As modelled, the shared beta represents the estimated change in treatment effect associated with a one-unit increase in the covariate: one year for age, 1 mL/kg/min for baseline VO_2_max, and one week for intervention duration. The shared beta was −1.09 (95% CrI −3.04 to 0.91) for age, −1.22 (−2.98 to 0.57) for baseline VO_2_max, and 0.42 (−1.19 to 1.96) for intervention duration; however, the wide CrIs indicate that these directions should not be interpreted as reliable moderator effects. Model fit was similar across the three models: DIC was 219.9, 220.3, and 221.3, respectively; residual deviance was 122.5, 122.6, and 123.2. Between-study SD estimates were also similar, at 1.65 (1.13 to 2.29), 1.63 (1.12 to 2.29), and 1.67 (1.15 to 2.36), respectively.

### 3.8 Subgroup analyses

Endurance sports (25 studies, 11 treatments): Full Bayesian NMA. IHT ranked first (SUCRA = 0.821), followed by BFR (0.671) and MICT (0.623) (full SUCRA rankings: **Supplementary Table 10**; visualized in **Supplementary Figure 10**). No treatment reached significance versus CG, with all 95% CrIs crossing zero. This reflects limited precision, not zero effect. All 12 node splitting comparisons in this subgroup were consistent.

Team ball (18), water (7), and combat/racket (10) sports: Networks were too sparse for NMA. Pairwise random-effects meta-analyses were run for treatments with direct CG comparisons (full results: **Supplementary Table 11**; forest plots: **Supplementary Figure 11**). In team ball sports, SIT showed a favorable estimate with k = 5 (MD 2.70 [1.17, 4.23]; p = 0.001), while HIIT did not reach significance (k = 4; MD 2.85 [−1.57, 7.28]; p = 0.207). RT (k = 2; MD 2.90 [0.69, 5.11]; p = 0.010) and SSG (k = 1; MD 5.30 [0.00, 10.60]; p = 0.050) should be interpreted only as directionally favorable exploratory estimates because the number of contributing studies was very small. In water sports, HIIT showed a directionally favorable but exploratory estimate (k = 2; MD 4.69 [1.76, 7.62]; p = 0.002), while SIT was not significant. In combat and racket sports, SIT (k = 5; MD 2.59 [1.20, 3.99]; p < 0.001) and HIIT (k = 3; MD 2.46 [0.75, 4.17]; p = 0.005) were favorable, while RT was not. BFR, AE, HVT, and MICT had zero direct CG comparisons in these subgroups. I² values of 0% across all pairwise comparisons (k = 2 to 5) should not be mistaken for genuine homogeneity; with few studies, the DerSimonian-Laird estimator truncates τ² to zero whenever Q < (k − 1), and statistical power to detect heterogeneity approaches zero.

### 3.9 Dose-response analysis

**Figure 4:**
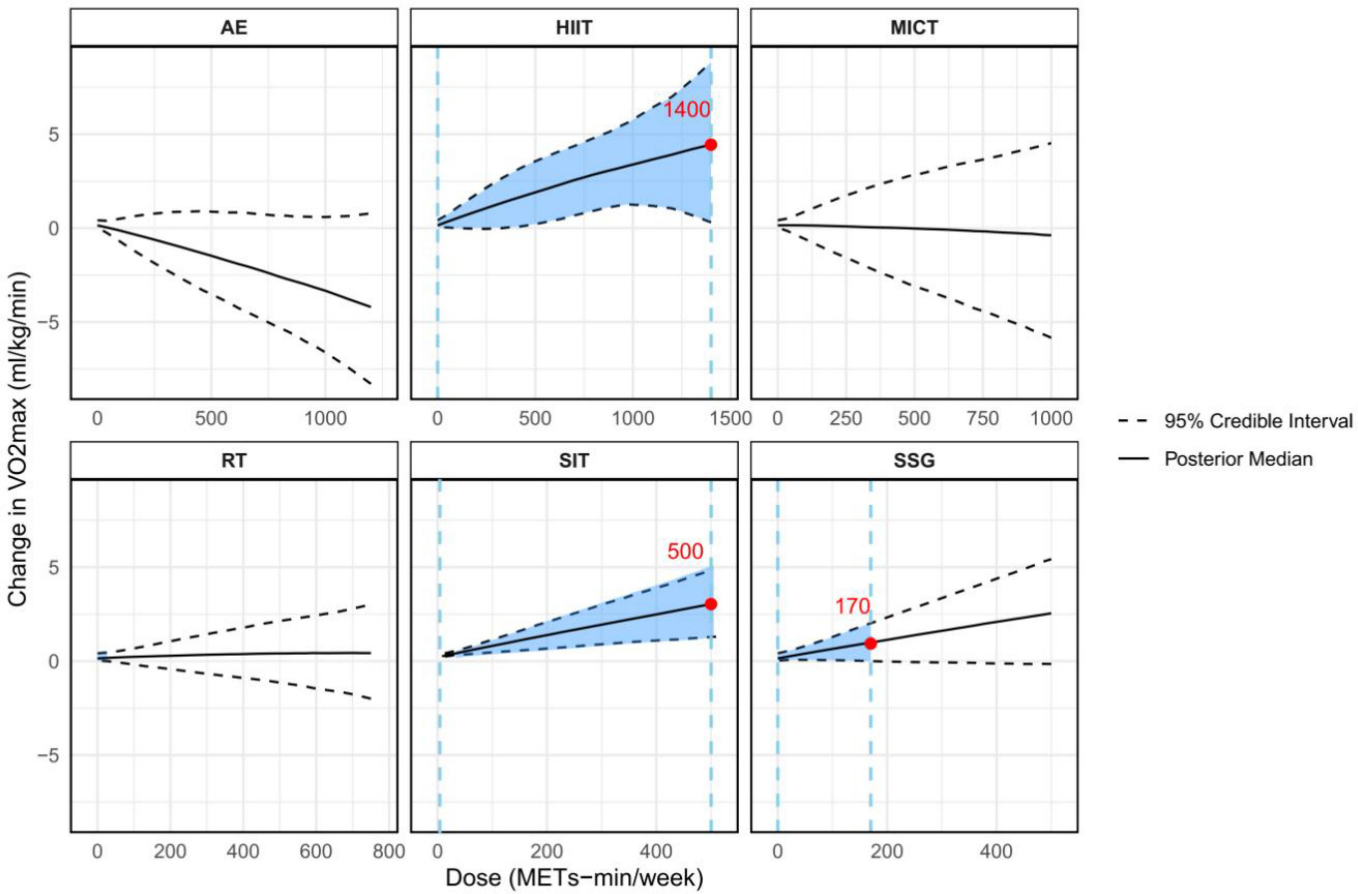
Dose-response curves.

Thirty-one studies entered the dose-response NMA (dose- and agent-level network connectivity: **Supplementary Figures S14-S15**). The quadratic function model fit best (DIC = 272.1) versus spline (284.2), log-linear (272.6), Emax (272.9), non-parametric monotonic (287.3), and exponential (288.3) models (**Supplementary Table 16**). Because the DIC differences between the quadratic, log-linear, and Emax models were < 1, the selected functional form should not be over-interpreted. For HIIT, SIT, and SSG, the modelled significant regions extended across the observed supplementary exercise dose ranges (**Figure 4**; **Supplementary Table 17**; full dose-response NMA summary in **Supplementary Table 18**). For HIIT, the observed data were compatible with progressively greater benefits across the studied dose range, with the largest estimated effect occurring at the highest observed dose (1400 METs-min/week; 4.43mL/kg/min [0.31,8.83]). Because the largest HIIT estimate occurred at the upper boundary of the observed data, this should not be interpreted as evidence of a biologically established optimum. SIT showed its largest model-estimated effect near 500 METs-min/week (2.99 [1.10, 4.98]), and SSG near 170 METs-min/week (1.01 [0.01, 2.02]). These values should be interpreted as model-estimated dose-response reference points within the observed data rather than established optimal prescriptions. AE, MICT, and RT showed no significant dose-response, with 95% CrIs crossing zero across the full dose range (**Supplementary Table 17**).

### 3.10 Publication bias and evidence quality

The comparison-adjusted funnel plot showed reasonable symmetry (**Supplementary Figure 12**), and Egger’s test did not detect statistically significant funnel plot asymmetry (p = 0.132), providing no statistical evidence of small-study effects. CINeMA assessment of 27 comparisons returned predominantly low or very low confidence ratings (**Supplementary Table 12**; contributing study-limitations network: **Supplementary Figure 13**). Imprecision, mainly wide CrIs, and heterogeneity were the main drivers of downgrading. No comparison reached high confidence. HIIT vs. CG (12 studies) and SIT vs. CG (11 studies) achieved low confidence, the highest ratings in the network.

## 4 Discussion

This systematic review included 61 studies; the main Bayesian NMA synthesized 60 RCTs across 11 training modalities, and the dose-response NMA included 31 studies with quantifiable supplementary dose data. Five modalities, BFR, HIIT, IHT, SIT, and SSG, had 95% CrIs excluding zero versus an active control condition consisting of continued sport-specific training, with model-estimated mean differences ranging from 2.49 to 3.93 mL/kg/min. In already-trained athletes, these estimates should be interpreted as uncertain marginal gains beyond ongoing sport-specific training rather than as guaranteed improvements, because baseline fitness, sport-specific training load, and ceiling effects may constrain further VO_2_max adaptation. Three of these, HIIT, SIT, and BFR, held up under both sensitivity analyses. The dose-response analysis found that the modelled significant region extended across the full observed supplementary exercise dose range for HIIT, SIT, and SSG, although the quadratic, log-linear, and Emax models were not materially separated by DIC. Across sport-type subgroup analyses, HIIT and SIT generally showed favorable estimates where data were available, but several subgroup estimates were exploratory and not statistically significant, especially in sparse non-endurance comparisons. The prespecified study-level network meta-regression did not detect clear effect modification by age, baseline VO_2_max, or intervention duration, but these null findings remain limited by aggregate data. Because most CINeMA ratings were low or very low, these findings should be treated as provisional rather than definitive practice guidance.

The strong showing of HIIT and SIT is broadly consistent with two recent network meta-analyses, but the scope and interpretation differ. Rosenblat et al. focused on training-intensity distribution in endurance athletes and found that intensity distribution influences maximal oxygen uptake and time-trial performance (Rosenblat et al., 2025). Yang et al. compared interval-training methods and reported favorable VO_2_max effects for HIIT, SIT, and repeated-sprint training in athletes (Yang et al., 2025). Our analysis agrees with those studies in supporting higher-intensity supplementary modalities, but extends the comparison to a broader set of 11 modalities, including SSG, BFR, IHT, RT, AE, and MICT. At the same time, our rankings should not be read as directly equivalent to those previous networks because the present analysis included a wider range of sports, a more heterogeneous active control condition, and a dose-response component that depended on MET-based exposure estimates. The magnitude of these estimates should be interpreted against prior athlete-focused syntheses. Previous pairwise meta-analyses generally support favorable VO_2_max effects of HIIT, SIT, and higher-intensity training distributions, but they also highlight substantial variation in protocols, athlete samples, and outcome assessment methods (Milanović et al., 2015; Wen et al., 2019; Yang et al., 2021; Rosenblat et al., 2025; Yang et al., 2025). Therefore, the present estimates should not be read as universal expected gains for all trained athletes. Between-study differences in VO_2_max testing protocols, including ramp versus step protocols and treadmill, cycle, rowing, swimming, or sport-specific ergometry, may have contributed to variation in observed change scores. Baseline training status and proximity to an adaptation ceiling may also affect how much additional VO_2_max improvement remains physiologically attainable.

BFR’s top SUCRA ranking (0.857) deserves particular caution. The point estimate versus CG was the largest in the network (3.93 mL/kg/min), but the credible interval was also wide [0.60, 7.27], and BFR had no direct CG comparisons in team ball, water, or combat/racket subgroups. This pattern suggests a promising but unstable signal rather than firm evidence of superiority. SUCRA captures ranking probability, not evidential weight (Salanti et al., 2011), and a high SUCRA value can coexist with sparse direct evidence and imprecise estimates. Although the comparison-adjusted funnel plot and Egger’s test did not provide statistical evidence of small-study effects, these methods have limited power when comparisons are few. Recent BFR reviews in athletes still report heterogeneous and uneven evidence (Yang et al., 2024; Zhang et al., 2025), so the top ranking should be interpreted as hypothesis-generating rather than as a basis for changing training guidelines.

The biological plausibility of BFR and IHT should therefore be separated from the certainty of the comparative evidence. BFR may increase local metabolic stress, intramuscular hypoxia-like signaling, fast-twitch fiber recruitment, and muscle perfusion adaptations at relatively low external workloads, which could provide a plausible pathway for improving oxygen extraction or utilization despite limited direct evidence (Yang et al., 2024; Zhang et al., 2025). IHT may influence VO_2_max through hypoxic signaling and, depending on the protocol, hematological and muscular adaptations related to oxygen transport and utilization, including changes in erythropoietic drive, hemoglobin mass, mitochondrial function, and muscle oxygen handling.

However, these mechanisms do not remove the methodological concern that normoxic and hypoxic IHT contexts were pooled in one node. Given the IHT-related inconsistency signals in node-splitting, both the BFR and IHT findings should be treated as biologically plausible but not yet definitive.

AE and MICT, two of the most widely recommended training modes, did not reach significance in either the primary NMA or the dose-response analysis. That result should be read in context. The athletes in these studies were not sedentary. They were already accumulating large volumes of sport-specific aerobic work. Adding more moderate-intensity exercise on top of that may simply fail to clear the threshold needed for further central cardiovascular adaptation, a ceiling effect you would not expect to see in untrained populations. The dose-response curves back this up: AE and MICT showed positive but non-significant trends across all doses. None of this means athletes should stop doing aerobic base work. AE and MICT underpin recovery, durability, and long-term development. The finding is about marginal gains: when the goal is pushing VO_2_max higher on top of an existing program, HIIT and SIT offer more return per unit of added training time.

The dose-response curves for HIIT, SIT, and SSG show that the modelled significant region, where the 95% CrI of the predicted VO_2_max change excludes zero, spans a broad dose range for each of these modalities (**Figure 4**; **Supplementary Tables S17-S18**). Importantly, the lower bound of the dose-response curve approaching zero should not be interpreted as no exercise. In the present analysis, a dose of zero represented the absence of additional supplementary training beyond regular sport-specific practice. Thus, the estimated effects reflect the incremental benefit of supplementary exercise added to an existing athletic training background. For HIIT, the significant region covers 0 to 1,400 METs-min/week, with the largest estimated effect at the highest observed dose (4.43 mL/kg/min [0.31, 8.83]). For SIT, the largest model-estimated effect occurred near 500 METs-min/week (2.99 [1.10, 4.98]); for SSG, near 170 METs-min/week (1.01 [0.01, 2.02]). AE, MICT, and RT showed no significant dose-response: the 95% CrI crossed zero across the full dose range.

The contrast between modalities that showed significant dose-response patterns (HIIT, SIT, and SSG) and those that did not (AE, MICT, and RT) is physiologically plausible, although not definitive. In trained athletes, further VO_2_max improvement usually requires a stimulus that challenges both central oxygen delivery and peripheral oxygen utilization beyond the athlete’s habitual sport-specific training. HIIT and SIT may provide such a stimulus through repeated exposure to severe or supramaximal intensity domains, high cardiac output, large stroke-volume demand, and substantial peripheral metabolic stress, which may promote both central cardiovascular and skeletal-muscle oxidative adaptations (Bassett and Howley, 2000; di Prampero, 2003; Joyner and Coyle, 2008; Levine, 2008; Gibala et al., 2012; Buchheit and Laursen, 2013a; Buchheit and Laursen, 2013b; MacInnis and Gibala, 2017). Redox regulation may also be relevant to oxygen-related adaptation, although evidence from hyperoxia-injury models, such as CXCR1-related glutamine metabolism and redox (glutathione and ROS) regulation, should be considered mechanistically indirect rather than direct evidence for exercise-training effects (Qin et al., 2023). For SIT specifically, the computed MET-min/week dose may underrepresent the true physiological stimulus because supramaximal bouts impose substantial anaerobic, neuromuscular, and recovery demands that are not fully captured by external-dose scaling. This helps explain why SIT can show favorable VO_2_max effects at a lower computed dose without implying that its internal load is actually low. SSG may act through repeated sport-specific high-intensity transitions, combining aerobic, anaerobic, and neuromuscular demands within an intermittent game-like context (Hill-Haas et al., 2011; Clemente et al., 2021). By contrast, AE and MICT may overlap more closely with the regular aerobic work already performed by many trained athletes; adding further moderate-intensity work may therefore produce diminishing marginal VO_2_max gains once a high aerobic baseline has been reached.

The shape of each dose-response curve should be interpreted cautiously. HIIT showed a positive modelled relationship across the observed range (0 to 1,400 MET-min/week), but because the largest estimate occurred at the upper boundary, this pattern may reflect the distribution of available HIIT data rather than a biological optimum. The SIT and SSG reference points were also model-estimated values derived from narrower observed dose ranges. Together with the small DIC differences among the quadratic, log-linear, and Emax models (**Supplementary Table 16**), these results should be treated cautiously given this model uncertainty.

The subgroup results expose a real tension. In endurance athletes, IHT ranked first (SUCRA = 0.821), yet no modality, IHT included, reached significance versus CG across 25 studies and 11 treatments. This is not evidence of no effect; it is evidence that 25 studies cannot deliver the precision needed when effects are moderate and between-study variability is real. The node-splitting inconsistency flagged specifically in IHT comparisons (Section 3.4), together with IHT’s removal in Sensitivity 2, suggests that IHT’s apparent edge in this subgroup may partly reflect network structure rather than a stable treatment effect. Future updates should split IHT into normoxic-IHT and hypoxic-IHT nodes if sufficient direct evidence becomes available.

The evidence that is missing is just as telling. BFR, AE, HVT, and MICT had zero direct comparisons with CG in team ball, water, and combat/racket athletes. That is a genuine knowledge gap, not a statistical footnote. Trials in these populations have almost exclusively pitted active treatments against each other, HIIT versus SIT, SIT versus SSG, without a shared control group that maintains regular sport-specific training. Without that common reference, you cannot estimate absolute effectiveness. The practical consequence is that for team sport and combat sport athletes, the evidence base for supplementary training beyond HIIT and SIT is sparse, and future trials in these populations need a CG arm to enable absolute effect estimation.

The biggest strength of this analysis is its scope: 61 studies in the systematic review, including 60 RCTs in the main NMA across 11 modalities, substantially larger than any prior NMA on this question. The dual approach of Bayesian NMA plus dose-response modeling addresses “which intervention” and “how much” in a single framework. The sensitivity analyses, network meta-regression, node splitting assessment, and CINeMA grading give a transparent view of what is robust and what is fragile in the evidence. The sport-type subgroup analyses also make the synthesis more relevant to coaching practice, where endurance, team sport, water sport, and combat/racket athletes differ in baseline training demands.

The limitations matter. The three smaller sport subgroups could not support full NMA and were restricted to pairwise meta-analysis. I² values of 0% in those comparisons (k = 2 to 5) should not be interpreted as genuine homogeneity: with only 2 to 5 studies per comparison, power to detect heterogeneity was essentially zero, and the DerSimonian-Laird estimator mechanically sets τ² to zero whenever Q falls below its degrees of freedom. The transitivity assumption also requires caution. Although all included participants met the eligibility definition of healthy athletes engaged in structured sport-specific training, the network combined adolescent team-sport players, adult and masters endurance athletes, and combat, racket, and water sport athletes with baseline VO_2_max values ranging from 35.25 to 81.00 mL/kg/min. The common comparator was not inactivity but continued regular sport-specific training; however, the absolute background training load embedded in this comparator, as well as competitive level and training history, was not consistently reported and likely differed substantially across sports. This heterogeneity may inflate between-study variance and affect SUCRA ranking probabilities, especially for modalities supported by sparse direct evidence or concentrated within specific sport types. The broad athlete definition also means that physiological maturity, training age, baseline aerobic capacity, and recovery capacity were likely different across adolescent team-sport players, adult combat-sport athletes, and masters endurance athletes. Therefore, rankings should be interpreted as network-level estimates conditional on the available evidence rather than as a universal hierarchy across all trained athletes.

The dose-response analysis was limited to 31 studies that reported enough detail for MET calculation. MET-min/week also has important conceptual limitations as a common dose metric: it standardizes external exposure but cannot fully capture internal load, intensity domain, anaerobic contribution, neuromuscular demand, metabolic stress, or post-exercise recovery cost. As a result, MET-based scaling may undercount supramaximal and resistance exercise and may partly explain why SIT appeared to produce relatively large effects at a lower computed dose. Sport-type-specific dose-response analyses could not be performed reliably because the 31 studies with quantifiable dose data became too sparse when stratified by sport category; therefore, the HIIT dose-response curve may partly reflect which sports contributed the high-dose data. The evidence base remained male-dominant, limiting inference for female athletes. Several treatment comparisons rest on single studies or purely indirect evidence, reflected in the predominantly low CINeMA ratings. The analysis did not include a low-risk-only sensitivity analysis, alternative pre-post correlation assumptions, or prior-sensitivity analysis. Funnel plot symmetry and Egger’s test offer limited reassurance when many comparisons involve few studies, and unpublished null findings cannot be ruled out.

From a practical perspective, the present findings may help prioritize hypotheses for supplementary training rather than define ready-to-apply prescriptions. HIIT and SIT were the most consistently supported modalities across the primary analysis and the sensitivity analyses, but the certainty of evidence was low and the estimates remain conditional on the available network structure. The model-estimated dose-response reference points for HIIT, SIT, and SSG should therefore be viewed as exploratory anchors for future research and individualized coaching discussion, not as prescriptive thresholds. BFR and IHT showed promising point estimates, but the direct evidence remains thin and uneven across sport types, so their use should be individualized and closely monitored. AE and MICT should not be dismissed, despite the absence of significant additional VO_2_max effects, because they may still support recovery, training tolerance, and long-term aerobic development.

Future trials should include common active control groups, compare modalities at matched supplementary doses, recruit more female athletes, report sex-disaggregated outcomes, provide sufficient training-dose data for MET-based analyses, standardize VO_2_max testing methodology, and distinguish normoxic from hypoxic IHT contexts.

## 5 Conclusions

This systematic review of 61 studies, including 60 randomized controlled trials in the main Bayesian network meta-analysis and 31 studies in the dose-response analysis, suggests that HIIT and SIT are the most consistently supported supplementary exercise modalities for improving VO_2_max in trained athletes. The dose-response results suggest favorable model-based patterns for HIIT, SIT, and SSG within the observed dose ranges, but these estimates should not be interpreted as established optimal prescriptions. Network meta-regression could not detect clear effect modification by age, baseline VO_2_max, or intervention duration. Given the predominantly low to very low certainty of evidence, sparse direct evidence for several modalities, and heterogeneity in sport-specific control training, these findings should be regarded as provisional estimates to guide future trials and cautious individualized interpretation rather than definitive training recommendations.

## Data Availability

The original contributions presented in the study are included in the article/**Supplementary Material**. Further inquiries can be directed to the corresponding author/s.
