# Supplementary material for: Comparative and dose-response effects of supplementary exercise training on VO2max in trained athletes: a Systematic Review and Bayesian network meta-analysis
Source: Front Physiol. 2026 Jul 30;17:1906583. doi: 10.3389/fphys.2026.1906583 (PMC13467872; doi:10.3389/fphys.2026.1906583)
Supplement: Supplementary file 1 [file DataSheet1.pdf]

Supplementary Appendix

1. Search Strategy

The following search strategies were executed on April 3, 2026. No language or date restrictions were applied.

1.1 PubMed (n = 1,171)

("Athletes"[Mesh] OR athlete*[Title/Abstract] OR "sports personnel"[Title/Abstract] OR sportsmen[Title/Abstract] OR sportswomen[Title/Abstract] OR "elite athletes"[Title/Abstract])
AND
("Exercise"[Mesh] OR "Exercise Therapy"[Mesh] OR "Motor Activity"[Mesh] OR "Sports"[Mesh] OR "Resistance Training"[Mesh] OR "Yoga"[Mesh] OR "Tai Ji"[Mesh] OR "Walking"[Mesh] OR "Physical Exertion"[Mesh] OR "physical activity"[Title/Abstract] OR movement[Title/Abstract] OR fitness[Title/Abstract] OR "aerobic exercise"[Title/Abstract] OR "high-intensity interval training"[Title/Abstract] OR "HIIT"[Title/Abstract] OR "resistance training"[Title/Abstract] OR "strength training"[Title/Abstract] OR "endurance exercise"[Title/Abstract] OR yoga[Title/Abstract] OR "Tai Chi"[Title/Abstract] OR pilates[Title/Abstract] OR dance[Title/Abstract] OR walking[Title/Abstract] OR jogging[Title/Abstract] OR stretching[Title/Abstract] OR "mind-body exercise"[Title/Abstract])
AND
("Oxygen Consumption"[Mesh] OR "Cardiorespiratory Fitness"[Mesh] OR "maximal oxygen uptake"[Title/Abstract] OR "maximal oxygen consumption"[Title/Abstract] OR VO2max[Title/Abstract] OR "VO2 max"[Title/Abstract] OR "maximal aerobic capacity"[Title/Abstract] OR "cardiorespiratory fitness"[Title/Abstract] OR "aerobic capacity"[Title/Abstract])
AND
(randomized controlled trial[pt] OR "Randomized Controlled Trial"[Title/Abstract] OR RCT[Title/Abstract] OR "randomized trial"[Title/Abstract] OR "controlled clinical trial"[Title/Abstract] OR "randomized study"[Title/Abstract] OR "randomized clinical trial"[Title/Abstract] OR RCTs[Title/Abstract] OR "randomized clinical studies"[Title/Abstract] OR "intervention trial"[Title/Abstract] OR "randomized intervention"[Title/Abstract] OR trial[Title/Abstract] OR "experimental study"[Title/Abstract])

1.2 Web of Science (n = 723)

TS=(athlete* OR "sports personnel" OR sportsmen OR sportswomen OR "elite athletes")
AND
TS=(exercise* OR "physical activity" OR movement OR fitness OR "aerobic exercise" OR "high-intensity interval training" OR HIIT OR "resistance training" OR "strength training" OR "endurance exercise" OR yoga OR "Tai Chi" OR "Tai Ji" OR pilates OR dance OR walking OR jogging OR stretching OR "mind-body exercise")
AND
TS=("maximal oxygen uptake" OR "maximal oxygen consumption" OR VO2max OR "VO2 max" OR "maximal aerobic capacity" OR "cardiorespiratory fitness" OR "aerobic capacity" OR "oxygen consumption")
AND
TS=("randomized controlled trial" OR RCT OR "randomized trial" OR "controlled clinical trial" OR "randomized study" OR "randomized clinical trial" OR RCTs OR "randomized clinical studies" OR "intervention trial" OR "randomized intervention" OR trial OR "experimental study")

1.3 Embase (n = 1,749)

('athlete'/exp OR athlete*:ti,ab OR 'sports personnel':ti,ab OR sportsmen:ti,ab OR sportswomen:ti,ab OR 'elite athletes':ti,ab)

AND
('exercise'/exp OR 'motor activity'/exp OR 'sport'/exp OR 'resistance training'/exp OR 'yoga'/exp OR 'tai chi'/exp OR 'walking'/exp OR 'physical activity':ti,ab OR movement:ti,ab OR fitness:ti,ab OR 'aerobic exercise':ti,ab OR 'high-intensity interval training':ti,ab OR hiit:ti,ab OR 'resistance training':ti,ab OR 'strength training':ti,ab OR 'endurance exercise':ti,ab OR yoga:ti,ab OR 'tai chi':ti,ab OR pilates:ti,ab OR dance:ti,ab OR walking:ti,ab OR jogging:ti,ab OR stretching:ti,ab OR 'mind-body exercise':ti,ab)
AND
('oxygen consumption'/exp OR 'cardiorespiratory fitness'/exp OR 'maximal oxygen uptake':ti,ab OR 'maximal oxygen consumption':ti,ab OR vo2max:ti,ab OR 'vo2 max':ti,ab OR 'maximal aerobic capacity':ti,ab OR 'cardiorespiratory fitness':ti,ab OR 'aerobic capacity':ti,ab)
AND
('randomized controlled trial'/exp OR 'controlled clinical trial'/exp OR 'randomized controlled trial':ti,ab OR rct:ti,ab OR 'randomized trial':ti,ab OR 'controlled clinical trial':ti,ab OR 'randomized study':ti,ab OR 'randomized clinical trial':ti,ab OR rcts:ti,ab OR 'randomized clinical studies':ti,ab OR 'intervention trial':ti,ab OR 'randomized intervention':ti,ab OR trial:ti,ab OR 'experimental study':ti,ab)

1.4 Cochrane Library (n = 667)

([mh "Athletes"] OR athlete*:ti,ab,kw OR "sports personnel":ti,ab,kw OR sportsmen:ti,ab,kw OR sportswomen:ti,ab,kw OR "elite athletes":ti,ab,kw)
AND
([mh "Exercise"] OR [mh "Exercise Therapy"] OR [mh "Motor Activity"] OR [mh "Sports"] OR [mh "Resistance Training"] OR [mh "Yoga"] OR [mh "Tai Ji"] OR [mh "Walking"] OR [mh "Physical Exertion"] OR "physical activity":ti,ab,kw OR movement:ti,ab,kw OR fitness:ti,ab,kw OR "aerobic exercise":ti,ab,kw OR "high-intensity interval training":ti,ab,kw OR HIIT:ti,ab,kw OR "resistance training":ti,ab,kw OR "strength training":ti,ab,kw OR "endurance exercise":ti,ab,kw OR yoga:ti,ab,kw OR "Tai Chi":ti,ab,kw OR pilates:ti,ab,kw OR dance:ti,ab,kw OR walking:ti,ab,kw OR jogging:ti,ab,kw OR stretching:ti,ab,kw OR "mind-body exercise":ti,ab,kw)
AND
([mh "Oxygen Consumption"] OR [mh "Cardiorespiratory Fitness"] OR "maximal oxygen uptake":ti,ab,kw OR "maximal oxygen consumption":ti,ab,kw OR VO2max:ti,ab,kw OR "VO2 max":ti,ab,kw OR "maximal aerobic capacity":ti,ab,kw OR "cardiorespiratory fitness":ti,ab,kw OR "aerobic capacity":ti,ab,kw)
AND
("randomized controlled trial":ti,ab,kw OR RCT:ti,ab,kw OR "randomized trial":ti,ab,kw OR "controlled clinical trial":ti,ab,kw OR "randomized study":ti,ab,kw OR "randomized clinical trial":ti,ab,kw OR RCTs:ti,ab,kw OR "randomized clinical studies":ti,ab,kw OR "intervention trial":ti,ab,kw OR "randomized intervention":ti,ab,kw OR trial:ti,ab,kw OR "experimental study":ti,ab,kw)

2.Definitions of exercise modalities

Table S1. Definitions of exercise modalities

Abbreviation	Full name	Definitions
AE	Aerobic Exercise	Aerobic exercise is performed by repeating sequences of light-to-moderate intensity activities for extended periods of time. e.g., walking, bicycle, and treadmill training etc.
BFR	Blood Flow Restriction	Blood flow restriction (BFR) training is a method where athletes exercise under controlled blood flow occlusion using pneumatic tourniquets applied to the proximal limbs to induce intense local hypoxia and metabolic accumulation, thereby stimulating cardiovascular adaptations and improving overall athletic performance.
CG	Control group	Maintenance of regular sport-specific training without additional structured supplementary exercise intervention
IHT	Intermittent Hypoxic Training	Intermittent hypoxic training (IHT) is a method where athletes rest or train under intermittent bouts of hypobaric or normobaric hypoxia to simulate altitude acclimatization and improve sea-level athletic performance.
SSG	Small-Sided Games	Small-sided games (SSG) are modified, scaled-down versions of team sports played on a reduced pitch with fewer players, designed to replicate the physical and technical demands of competition while targeting specific physiological adaptations.
RT	Resistance Training	Exercise training designed to improve the strength, power, endurance and size of skeletal muscles.
SIT	Sprint Interval Training	Sprint interval training (SIT) is a form of high-intensity intermittent exercise that involves repeated bouts of very short (≤30 s), all-out maximal effort sprints interspersed with extended periods of active or passive recovery (2–4 min), aimed at enhancing both anaerobic and aerobic energy systems.
HIIT	High-Intensity Interval Training	It typically consists of alternating high-intensity exercise lasting 15 seconds to 4 minutes with passive or low-intensity recovery periods. The heart rate during the high-intensity phase approaches 80% to 95% of the maximum heart rate.
POL	Polarized Training	Polarized Training (POL) is a periodization model that concentrates training intensity distribution predominantly at low intensities (zone 1, <

Abbreviation	Full name	Definitions
		first ventilatory threshold) and high intensities (zone 3, > second ventilatory threshold) with very little work in the moderate-intensity zone (zone 2), typically following a 75−80% / 5−10% / 15−20% distribution, aiming to maximize endurance performance adaptations.
HVT	High-Volume Training	High-volume training (HVT) is a continuous endurance training method performed at low to moderate intensities (typically 60−75% of maximal heart rate or below the first ventilatory threshold) for prolonged durations per session, aimed at enhancing aerobic capacity through high accumulative training volume.
MICT	Moderate-Intensity Continuous Training	Moderate-intensity continuous training (MICT) is a form of aerobic exercise performed at a steady, submaximal intensity (typically 70–80% of maximal heart rate or between the first and second ventilatory thresholds) for a sustained duration without rest intervals, aiming to improve cardiorespiratory fitness and metabolic function.
THR	Threshold Training	Threshold training (THR) is a training method where athletes perform a high volume of exercise at an intensity just below, at, or slightly above the lactate threshold (LT) to improve the body's ability to sustain a high work rate before fatigue from lactate accumulation occurs.

3. Study Characteristics

Table S2. Characteristics of included studies — Network Meta-Analysis.

Studyname	Interventions (n)	Sports items	Gender	baseline	Length(weeks)	Age (mean±sd)	Region
aagaard et al.2011[1]	RT (7)	cyclists	Male	73.5 (8.2)	16	19.5(0.8)	Denmark
	CG (7)		Male	71.5 (6.0)	16	19.5(0.8)	
Arslan et al.2022[2]	HIIT (6)	basketball	Male	45.8 (0.85)	6	14.5(0.5)	Turkey
	SSG (6)		Male	46.29 (1.03)	6	14.5(0.5)	
Bicer et al.2020[3]	RT (13)	volleyball	Male	37.85 (4.34)	8	27.08(3.99)	Turkey
	CG (13)		Male	51.81(19.03)	8	27.46(4.05)	
Bilici et al.2026[4]	HIIT (11)	volleyball	Female	36.73 (2.37)	12	16.18(1.17)	Turkey
	HIIT (10)		Female	37.40 (4.86)	12	16.00(1.05)	
	CG (11)		Female	37.82 (3.49)	12	16.64(1.29)	
Campo et al.2015[5]	IHT (9)	Triathlon	Male	59.5 (5.0)	7	26.0(6.7)	Spain
	CG (9)		Male	58.9 (4.5)	7	29.3(6.8)	
Chéilleachair et al.2017[6]	AE (9)	rowers	Mix	61.7(6.6)	8	22(4)	Ireland
	HIIT (10)		Mix	60.6(5.3)	8	22(4)	
Chen et al.2025[7]	BFR (17)	basketball	Male	39.48(6.22)	6	19.70(1.31)	China
	HIIT (16)		Male	39.75(6.92)	6	19.87(1.25)	
Clark et al.2010[8]	HIIT (17)	soccer	Female	49.69(1.15)	8	18.7(0.37)	USA
	AE (15)		Female	49.54(1.89)	8	18.6(0.51)	
Czuba et al.2017[9]	HIIT (7)	swimmers	Male	54.0(6.2)	4	20.5(1.3)	Poland

	IHT (8)		Male	56.0(4.0)	4	19.1(1.3)	
	AE (10)		Male	67.0(2.9)	4	21.8(4.0)	
Czuba et al.2018[10]	IHT (10)	cyclists	Male	66.0(4.0)	4	20.5(2.9)	Poland
	IHT (10)		Male	67.6(2.7)	4	20.7(3.1)	
driller et al.2009[11]	HIIT (10)	rowers	Mix	53.2(6.4)	4	19(2)	Australia
	CG (10)		Mix	54.8(7.3)	4	19(2)	
dufour et al.2006[12]	AE (9)	endurance runners	Male	61.5(1.1)	6	30.3(6.1)	France
	IHT (9)		Male	64.2(1.2)	6	30.3(6.3)	
Fernandez et al.2012[13]	HIIT (11)	tennis	Male	56.3(4.0)	6	22.6(4.8)	Germany
	CG (9)		Male	57.3(4.0)	6	22.1(3.3)	
Gantois et al.2022[14]	SIT (10)	volleyball	Male	50.39(4.30)	6	21.6(3.34)	Brazil
	CG (9)		Male	48.84(5.89)	6	21.33(2.91)	
held et al.2020[15]	AE (15)	rowers	Mix	63.2(8.5)	5	21.7(3.7)	Germany
	BFR (16)		Mix	63.0(7.0)	5	21.9(3.2)	
	MICT (7)		Mix	80(4)	10	28(5)	
Jarstad et al.2019[16]	HIIT (7)	endurance athletes	Mix	81(3)	10	28(5)	Norway
	CG (6)		Mix	77(4)	10	28(5)	
javaloyes et al.2019[17]	MICT (9)	cyclists	Male	56.34(7.58)	8	39.22(5.33)	spain
	AE (8)		Male	54.30(7.81)	8	37.62(7.09)	
Jin et al.2025.January[18]	HIIT (12)	swimmers	Mix	51.48(3.85)	4	19.94(1.4)	China
	CG (12)		Mix	49.40(3.75)	4	21.17(1.6)	

Jin et al.2025.March[19]	AE (10)	distance runners	Male	68.8(7.0)	6	20(1)	China
	SIT (10)		Male	67.2(5.0)	6	19(1)	
kaynak et al.2017[20]	SIT (9)	volleyball	Male	50(3.6)	6	21.2(1.3)	volleyball
	CG (9)		Male	50.4(4)	6	21.2(1.6)	
kelly et al.2021[21]	SIT (13)	football	Male	52(6)	6	26.5(4.9)	Turkey
	CG (12)		Male	53.4(4.7)	6	25.4(2.6)	
Kilit et al.2018[22]	HIIT (14)	tennis	Male	45.4(1.9)	6	13.8(0.4)	Turkey
	SSG (15)		Male	45.9(1.7)	6	13.8(0.4)	
Kim et al.2011[23]	SIT (11)	Judo	Male	49.84(4.08)	8	20.00(1.10)	Korea
	CG (19)		Male	46.63(4.99)	8	19.94(1.16)	
Kirchenberger et al.2021[24]	HIIT (10)	Rowers	Male	59.4(2.3)	8	15.4(1.3)	Germany
	MICT (7)		Male	58.4(5.7)	8	15.3(1.2)	
Kristoffersen et al.2019[25]	RT (16)	cyclists	Mix	61.1(6.0)	6	29.6(0.6)	Norway
	SIT (16)		Mix	63.3(5.9)	6	29.6(0.6)	
Latino et al.2024[26]	HIIT (15)	volleyball	Male	48.45(1.88)	16	19.14(1.61)	Italy
	CG (15)		Male	47.78(2.42)	16	19.14(1.61)	
Liu et al.2021[27]	SIT (8)	Badminton	Male	56.8(7.0)	8	20.0(1.3)	China
	CG (8)		Male	55.8(8.0)	8	21.5(2.2)	
Liu et al.2023[28]	SIT (8)	swimmers	Male	54.6(3.6)	4	25(6)	China
	CG (8)		Male	54.5(3.5)	4	25(6)	
Maggioni et al.2019[29]	SSG (12)	Basketball	NR	57.4(6.7)	8	19(1)	Italy

	SIT (9)		NR	55.7(7.2)	8	19(1)	
	CG (9)		NR	56.7(2.8)	8	19(1)	
Mallol et al.2019[30]	HIIT (8)	triathletes	Mix	42.4(5.2)	4	42.9(12.1)	Australia
	CG (8)		Mix	43.1(4.5)	4	37.2(13.3)	
meckel et al.2012[31]	HIIT (13)	soccer	Male	44.9(5.2)	7	14.5(0.6)	Israel
	SIT (11)		Male	44.6(5.3)	7	14.5(0.6)	
Millet et al.2002[32]	RT (7)	triathletes	NR	69.7(3.60	14	24.3(5.2)	France
	CG (8)		NR	67.6(6.4)	14	21.4(2.1)	
MonKS et al.2017[33]	HIIT (16)	Taekwondo	Mix	56.1(1.38)	4	18-22	Korea
	AE (17)		Mix	51.5(1.34)	4	18-22	
Morais et al.2025[34]	HIIT (12)	tennis	Male	45.21(2.06)	6	13.6(0.3)	Spain
	SIT (12)		Male	45.50(1.85)	6	13.6(0.3)	
Muñoz et al.2021[35]	HIIT (10)	Field Hockey	Female	37.50(6.04)	8	23.6(2.8)	Chile
	CG (10)		Female	35.25(5.50)	8	23.6(3.0)	
	SIT (13)		Mix	51.1(4.9)	4	16(1)	
ouergui et al.2020[36]	SIT (13)	Taekwondo	Mix	50.9(3.4)	4	16(1)	Tunisia
	CG (13)		Mix	48.3(3.2)	4	16(1)	
ØVRETVEIT et al.2019[37]	HIIT (6)	BJJ	Male	52.7(5.3)	6	30.3(4.0)	Norway
	CG (6)		Male	53.3(1.2)	6	30.3(4.0)	
Pérez et al.2019[38]	POL (11)	ultra-endurance	Male	55.8(4.9)	12	40.6(9.7)	Spain
	THR (9)	runners	Male	57.1(5.2)	12	36.8(9.2)	

ponsot et al.2006[39]	HIIT (7)	endurance runners	Male	62.0(1.4)	6	31.3(2.3)	France
	IHT (8)		Male	63.6(1.1)	6	29.9(2.3)	
Poyatos et al.2022[40]	MICT (6)	runners	NR	58.8(4.99)	8	31.5(8.36)	Spain
	AE (6)		NR	61(7.37)	8	31.2(10.3)	
Pugliese et al.2018[41]	AE (10)	Runners	Male	47.6(4.2)	8	47.2(7.4)	Italy
	HIIT (10)		Male	48.8(5.5)	8	47.2(7.4)	
	CG (10)		Male	48.4(4.4)	8	47.2(7.4)	
robach et al.2012[42]	AE (6)	endurance runners	Mix	70.8(5.3)	4	29(6)	France
	IHT (10)		Mix	73.5(2.9)	4	29(6)	
Rodriguez et al.2007[43]	AE (12)	endurance runners	Mix	55.8(8.0)	4	22.8(9.3)	USA
	IHT (11)		Mix	56.7(8.9)	4	22.2(7.0)	
Roels et al.2005[44]	AE (8)	endurance runners	Male	62.43(5.31)	7	33.0(2.85)	France
	IHT (10)		Male	70.43(5.55)	7	22.8(2.5)	
	IHT (11)		Male	57.08(7.30)	7	21.9(2.8)	
Ronnestad et al.2010.August[45]	RT (6)	cyclists	Male	65.2(2.2)	25	29(3)	Norway
	CG (6)		Mix	67.3(2.7)	25	31(3)	
Rønnestad et al.2010.November[46]	RT (11)	cyclists	Male	66.8(1.6)	12	27(2)	Norway
	CG (9)		Mix	65.9(2.0)	12	30(2)	
Sandbakk et al.2013[47]	HIIT (7)	SKIERS	Mix	67.4(7.7)	8	17.5(0.4)	Norway
	AE (7)		Mix	67.4(7.7)	8	17.5(0.4)	
Sheykhlouvand et al.2024[48]	SIT (10)	soccer	Male	51.57(2.6)	6	25.8(3.5)	Iran

	SSG (10)		Male	51.84(2.6)	6	26.2(4.0)	
	HIIT (10)		Male	46.4(3.0)	6	19.8(1.3)	
Song et al.2024[49]	HIIT (10)	Taekwondo	Male	48.8(2.5)	6	19.8(1.3)	Iran
	CG (10)		Male	46.6(2.9)	6	19.8(1.3)	
Sperlich et al.2011[50]	HIIT (8)	soccer	Male	55.1(4.9)	5	13.5(0.4)	Germany
	HVT (8)		Male	55.3(4.3)	5	13.5(0.4)	
	HIIT (10)		Mix	63.7(7.1)	9	31(6)	
Stöggl et al.2014[51]	HVT (11)	endurance athletes	Mix	60.5(9.4)	9	31(6)	Germany
	POL (11)		Mix	60.6(8.3)	9	31(6)	
	THR (8)		Mix	63.2(4.6)	9	31(6)	
Talsnes et al.2022[52]	AE (22)	Cross-Country	Mix	65.7(7.6)	8	18(1)	Norway
	HIIT (20)	Skiing、Biathlon	Mix	66.7(7.1)	8	18(1)	
	AE (16)		Male	55.9(5.7)	12	41.5(4.5)	
Tangchaisuriya et al.2022[53]	BFR (17)	cyclists	Male	54.8(4.7)	12	39.6(3.8)	Thailand
	HIIT (17)		Male	55.2(6.7)	12	41.7(3.8)	
ventura et al.2003[54]	AE (7)	cyclists	Mix	62.3(1.5)	6	25(2.2)	Switzerland
	IHT (7)		Mix	62.1(2.7)	6	25(2.2)	
	HIIT (5)		NR	45.2(3.6)	5	18-20	
Wang et al.2022[55]	MICT (5)	Basketball	NR	46.2(3.7)	5	18-20	China
	MICT (5)		NR	46.5(3.7)	5	18-20	
Wei et al.2025[56]	HIIT (14)	volleyball	Male	45.55(5.34)	8	20.7(1.4)	China

Wells et al.2014[57]	SIT (14)	soccer	Male	46.93(4.25)	8	19.8(2.67)	UK
	HIIT (8)		Male	57.6(5.4)	8	21.3(2.1)	
	CG (8)		Male	57.1(3.6)	8	21.3(2.1)	
Xu et al.2025[58]	SIT (8)	Karate	Male	41.9(2.7)	8	21.4(2.2)	China
	RT (8)		Male	42.3(2.8)	8	21.7(2.4)	
	CG (8)		Male	41.8(2.6)	8	21.2(2.4)	
Yang et al.2024[59]	SIT (10)	soccer	Male	50.2(2.3)	6	22.4(1.6)	China
	RT (10)		Male	50.1(2.2)	6	21.2(1.4)	
	CG (10)		Male	50.1(2.6)	6	22.2(1.9)	
zoll et al.2006[60]	AE (6)	distance runners	Male	64.3(1.2)	6	31.3(3.1)	France
	IHT (9)		Male	59.4(1.0)	6	30.3(2.1)	

AE: Aerobic Exercise; BFR: Blood Flow Restriction (or Aquatic Exercise); CG: Control Group; IHT: Intermittent Hypoxic Training; SSG: Small-Sided Games; RT: Resistance Training; SIT: Sprint Interval Training; HIIT: High-Intensity Interval Training; POL: Polarized Training; HVT: High-Volume Training; MICT: Moderate-Intensity Continuous Training; THR: Threshold Training; NR: Not Report.

Table S3. Characteristics of included studies — Dose-Response Analysis.

Studyname	Interventions (n)	Baseline	length (week)	Age (mean±sd)	Region	Sports items	Gender
aagaard et al.2011[1]	RT (7)	73.5(8.2)	16	19.5(0.8)	Denmark	cyclists	Male
	CG (7)	71.5(6.0)	16	19.5(0.8)			Male

Bicer et al.2020[3]	RT (13)	37.85(4.34)	8	27.08(3.99)	Turkey	volleyball	Male
	CG (13)	51.81(19.03)	8	27.46(4.05)			Male
Bilici et al.2026[4]	HIIT (10)	37.40(4.86)	12	16.00(1.05)	Turkey	volleyball	Female
	HIIT (11)	36.73(2.37)	12	16.18(1.17)			Female
	CG (11)	37.82(3.49)	12	16.64(1.29)			Female
Fernandez et al.2012[13]	HIIT (11)	56.3(4.0)	6	22.6(4.8)	Germany	tennis	Male
	SIT (12)	55.6(5.0)	6	21.2(5.1)			Male
	CG (9)	57.3(4.0)	6	22.1(3.3)			Male
Gantois et al.2022[14]	SIT (10)	50.39(4.30)	6	21.6(3.34)	Brazil	volleyball	Male
	CG (9)	48.84(5.89)	6	21.33(2.91)			Male
Jarstad et al.2019[16]	HIIT (7)	50.6(5.4)	10	28(5)	Norway	endurance athletes	Mix
	MICT (7)	50.9(7.1)	10	28(5)			Mix
	CG (6)	54.5(7.3)	10	28(5)			Mix
Jin et al.2025.March[19]	HIIT (12)	51.48(3.85)	4	19.94(1.4)	China	swimmers	Mix
	CG (12)	49.40(3.75)	4	21.17(1.6)			Mix
Jin et al.2025.January[18]	SIT (10)	67.2(5.0)	6	19(1)	China	distance runners	Male
	AE (10)	68.8(7.0)	6	20(1)			Male
kaynak et al.2017[20]	SIT (9)	50(3.6)	6	21.2(1.3)	Turkey	volleyball	Male
	CG (9)	50.4(4)	6	21.2(1.6)			Male
Kim et al.2011[23]	SIT (11)	49.84(4.08)	8	20.00(1.10)	Korea	Judo	Male
	CG (19)	46.63(4.99)	8	19.94(1.16)			Male

Kirchenberger et al.2021[24]	HIIT (10)	58.4(3.9)	8	15.4(1.3)	Germany	Rowers	Male
	MICT (7)	58.4(5.7)	8	15.3(1.2)			Male
Kristoffersen et al.2019[25]	SIT (16)	63.3(5.9)	6	29.6(0.6)	Norway	cyclists	Mix
	RT (16)	61.1(6.0)	6	29.6(0.6)			Mix
Liu et al.2023[28]	SIT (8)	54.6(3.6)	4	25(6)	Korea	swimmers	Male
	CG (8)	54.5(3.5)	4	25(6)			Male
Maggioni et al.2019[29]	SSG (12)	57.4(6.7)	8	19(1)	Italy	Basketball	NR
	SIT (9)	55.7(7.2)	8	19(1)			NR
	CG (9)	56.7(2.8)	8	19(1)			NR
Mallol et al.2019[61]	HIIT (8)	42.4(5.2)	4	42.9(12.1)	Australia	triathletes	Mix
	CG (8)	43.1(4.5)	4	37.2(13.3)			Mix
Millet et al.2002[32]	RT (7)	69.7(3.60)	14	24.3(5.2)	France	triathletes	NR
	CG (8)	67.6(6.4)	14	21.4(2.1)			NR
MonKS et al.2017[33]	HIIT (16)	56.1(1.38)	4	18-22	Korea	Taekwondo	Mix
	AE (17)	51.5(1.34)	4	18-22			Mix
Morais et al.2025[34]	HIIT (12)	45.21(2.06)	6	13.6(0.3)	Spain	tennis	Male
	SIT (12)	45.50(1.85)	6	13.6(0.3)			Male
Muñoz et al.2021[35]	HIIT (10)	37.50(6.04)	8	23.6(2.8)	Chile	Field Hockey	Female
	CG (10)	35.25(5.50)	8	23.6(3.0)			Female
ouergui et al.2020[36]	SIT (13)	50.9(3.4)	4	16(1)	Tunisia	Taekwondo	Mix
	SSG (13)	51.1(4.9)	4	16(1)			Mix

	CG (13)	48.3(3.2)	4	16(1)			Mix
ØVRETVEIT et al.2019[37]	HIIT (6)	52.7(5.3)	6	30.3(4.0)	Norway	BJJ	Male
	CG (6)	53.3(1.2)	6	30.3(4.0)			Male
Ronnestad et al.2010.August[45]	RT (6)	65.2(2.2)	25	29(3)	Norway	cyclists	Male
	CG (6)	67.3(2.7)	25	31(3)			Mix
Rønnestad et al.2010.November[46]	RT (11)	66.8(1.6)	12	27(2)	Norway	cyclists	Male
	CG (9)	65.9(2.0)	12	30(2)			Mix
Sandbakk et al.2013[47]	HIIT (7)	68(8.4)	8	17.5(0.4)	Norway	SKIERS	Mix
	HIIT (7)	67.4(7.7)	8	17.5(0.4)			Mix
	AE (7)	66.6(9.6)	8	17.5(0.4)			Mix
Sheykhlouvand et al.2024[48]	SSG (10)	51.84(2.6)	6	26.2(4.0)	Iran	soccer	Male
	SIT (10)	51.57(2.6)	6	25.8(3.5)			Male
Song et al.2024[49]	SIT (10)	48.8(2.5)	6	19.8(1.3)	Iran	Taekwondo	Male
	SSG (10)	46.4(3.0)	6	19.8(1.3)			Male
	CG (10)	46.6(2.9)	6	19.8(1.3)			Male
Wei et al.2025[56]	SIT (14)	46.93(4.25)	8	19.8(2.67)	China	volleyball	Male
	HIIT (14)	45.55(5.34)	8	20.7(1.4)			Male
Wells et al.2014[57]	HIIT (8)	57.6(5.4)	8	21.3(2.1)	UK	soccer	Male
	CG (8)	57.1(3.6)	8	21.3(2.1)			Male
Xu et al.2025[58]	RT (8)	41.9(2.7)	8	21.4(2.2)	China	Taekwondo	Male
	SIT (8)	42.3(2.8)	8	21.7(2.4)			Male

	CG (8)	41.8(2.6)	8	21.2(2.4)			Male
Yang et al.2024[59]	RT (10)	50.1(2.2)	6	21.2(1.4)	China	soccer	Male
	SIT (10)	50.2(2.3)	6	22.4(1.6)			Male
	CG (10)	50.1(2.6)	6	22.2(1.9)			Male
Yu et al.2025[62]	RT (16)	64.92(5.18)	8	16.81(0.61)	China	endurance runners	Male
	RT (16)	65.84(5.95)	8	16.75(0.68)			Male

AE: Aerobic Exercise; BFR: Blood Flow Restriction (or Aquatic Exercise); CG: Control Group; IHT: Intermittent Hypoxic Training; SSG: Small-Sided Games; RT: Resistance Training; SIT: Sprint Interval Training; HIIT: High-Intensity Interval Training; POL: Polarized Training; HVT: High-Volume Training; MICT: Moderate-Intensity Continuous Training; THR: Threshold Training; NR: Not Report.

4.Network Geometry

Table S4. Network geometry summary.

Characteristic	Value
Number of studies	60
Number of treatment arms (after pooling)	135
Number of treatments (nodes)	12
Number of pairwise comparisons	36
Number of study designs	14
Multi-arm studies	4 (3-arm: 3; 4-arm: 1)
Reference treatment	CG (Control group)

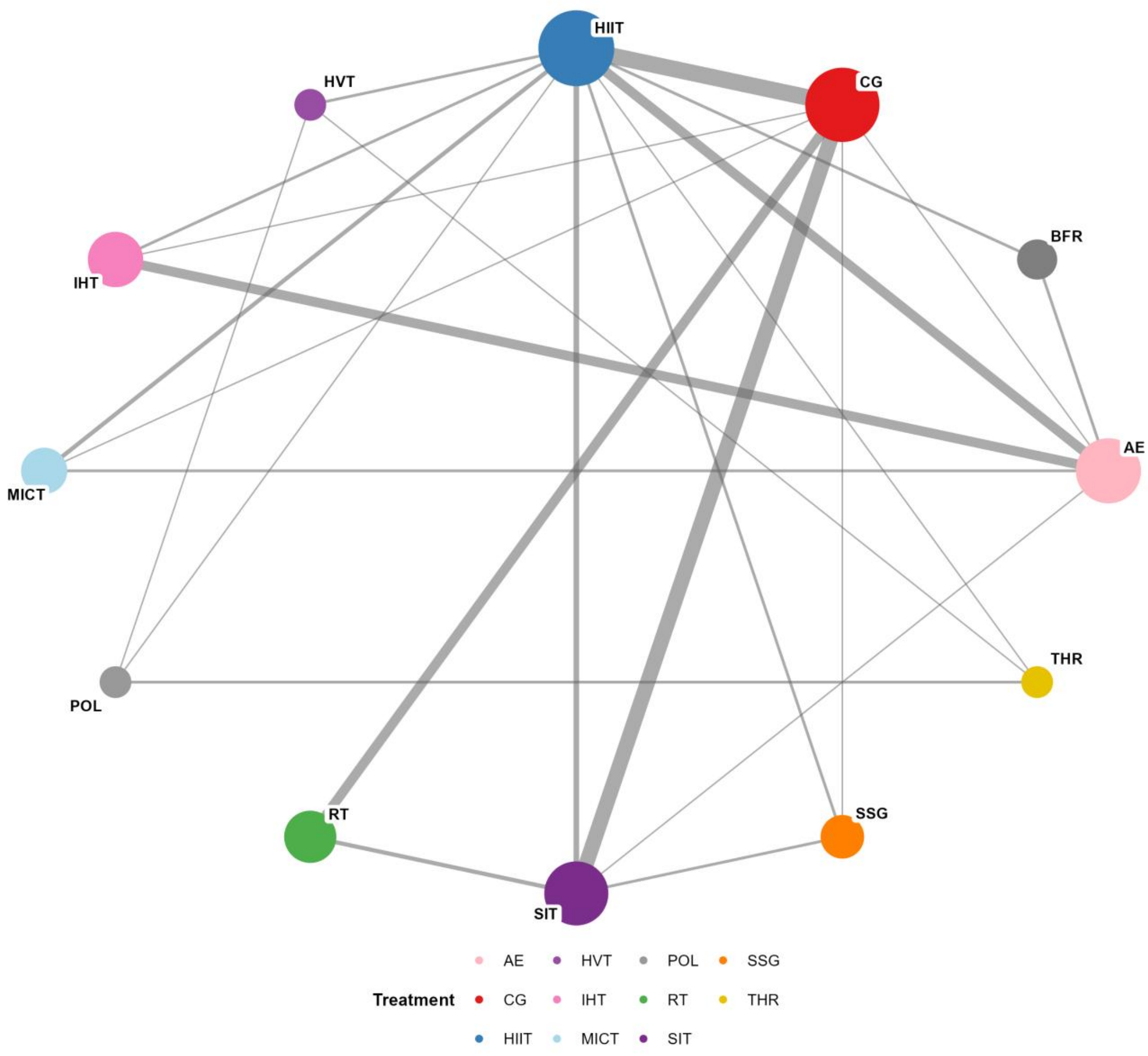

Figure S1. Network plot

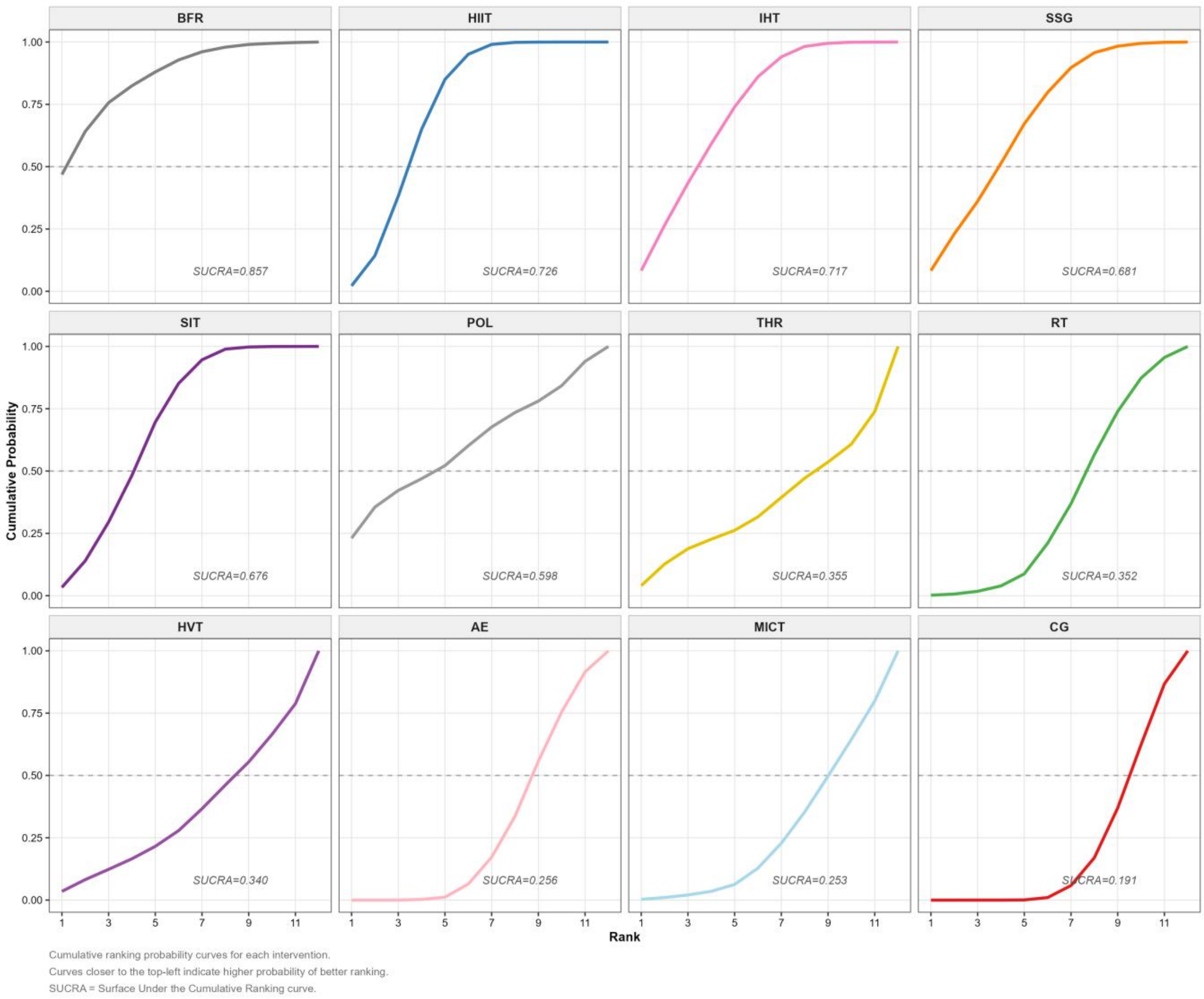

Figure S2. SUCRA rankings plot

5.Risk of Bias Assessment

Risk of bias domains

Study	D1	D2	D3	D4	D5	Overall
Aagaard et al.2011	+	+	+	+	+	+
Arslan et al.2022	+	+	+	+	+	+
Bicer et al.2020	-	+	+	+	+	-
Bilici et al.2026	+	+	+	+	+	+
Campo et al.2015	+	-	+	+	+	-
Cheilleachair et al.2017	+	+	+	+	+	+
Chen et al.2025	+	+	+	+	+	+
Clark et al.2010	-	-	+	+	+	-
Czuba et al.2017	+	+	+	+	+	+
Czuba et al.2018	+	+	+	+	+	+
driller et al.2009	+	+	+	+	+	+
dufour et al.2006	-	-	+	+	+	-
Fernandez et al.2012	+	X	+	+	-	X
Gantois et al.2022	+	+	-	+	+	-
held et al.2020	+	+	+	+	+	+
Jarstad et al.2019	+	+	-	+	+	-
javaloyes et al.2019	+	+	+	+	+	+
Jin et al.2025.March	+	-	+	+	+	-
Jin et al.2025.January	+	+	+	+	+	+
kaynak et al.2017	+	+	+	+	+	+
kelly et al.2021	+	+	+	+	+	+
Kilit et al.2018	-	X	+	+	+	X
Kim et al.2011	+	+	+	+	+	+
Kirchenberger et al.2021	+	-	+	+	+	-
Kristoffersen et al.2019	+	X	-	+	+	X
Latino et al.2024	+	+	+	+	+	+
Liu et al.2021	+	+	+	+	+	+
Liu et al.2023	+	+	+	+	+	+
Maggioni et al.2019	+	+	+	+	+	+
Mallol et al.2019	+	-	+	+	+	-
meckel et al.2012	+	X	+	+	+	X
Millet et al.2002	+	+	+	+	+	+
MonKS et al.2017	+	-	+	+	+	-
Morais et al.2025	+	+	+	+	+	+
Munoz et al.2021	+	+	+	+	+	+
ouergui et al.2020	+	-	+	+	+	-
OVRETVEIT et al.2019	+	-	+	+	+	-
Perez et al.2019	-	+	+	+	-	-
ponsot et al.2006	+	-	+	+	+	-
Poyatos et al.2022	+	+	+	+	+	+
Pugliese et al.2018	+	-	+	+	+	-
robach et al.2012	+	+	+	+	+	+
Rodriguez et al.2007	+	X	+	+	+	X
Roels et al.2005	+	+	+	+	+	+
Ronnestad et al.2010.August	-	X	+	+	+	X
Ronnestad et al.2010.November	+	+	+	+	+	+
Sandbakk et al.2013	+	-	+	+	+	-
Sheykhlouvand et al.2024	+	+	+	+	+	+
Song et al.2024	+	+	+	+	+	+
Sperlich et al.2011	+	-	+	+	-	-
Stoggl et al.2014	+	+	-	+	+	-
Talsnes et al.2022	+	+	+	+	+	+
Tangchaisuriya et al.2022	+	+	+	+	+	+
ventura et al.2003	+	+	+	+	+	+
Wang et al.2022	+	-	+	+	-	-
Wei et al.2025	+	+	+	+	+	+
Wells et al.2014	+	+	+	+	+	+
Xu et al.2025	+	+	+	+	+	+
Yang et al.2024	+	+	+	+	+	+
Yu et al.2025	+	+	+	+	+	+
zoll et al.2006	+	+	+	+	+	+

Domains:
D1: Bias due to randomisation.
D2: Bias due to deviations from intended intervention.
D3: Bias due to missing data.
D4: Bias due to outcome measurement.
D5: Bias due to selection of reported result.

Judgement
X High
- Some concerns
+ Low

Figure S3. The detail of risk of bias.

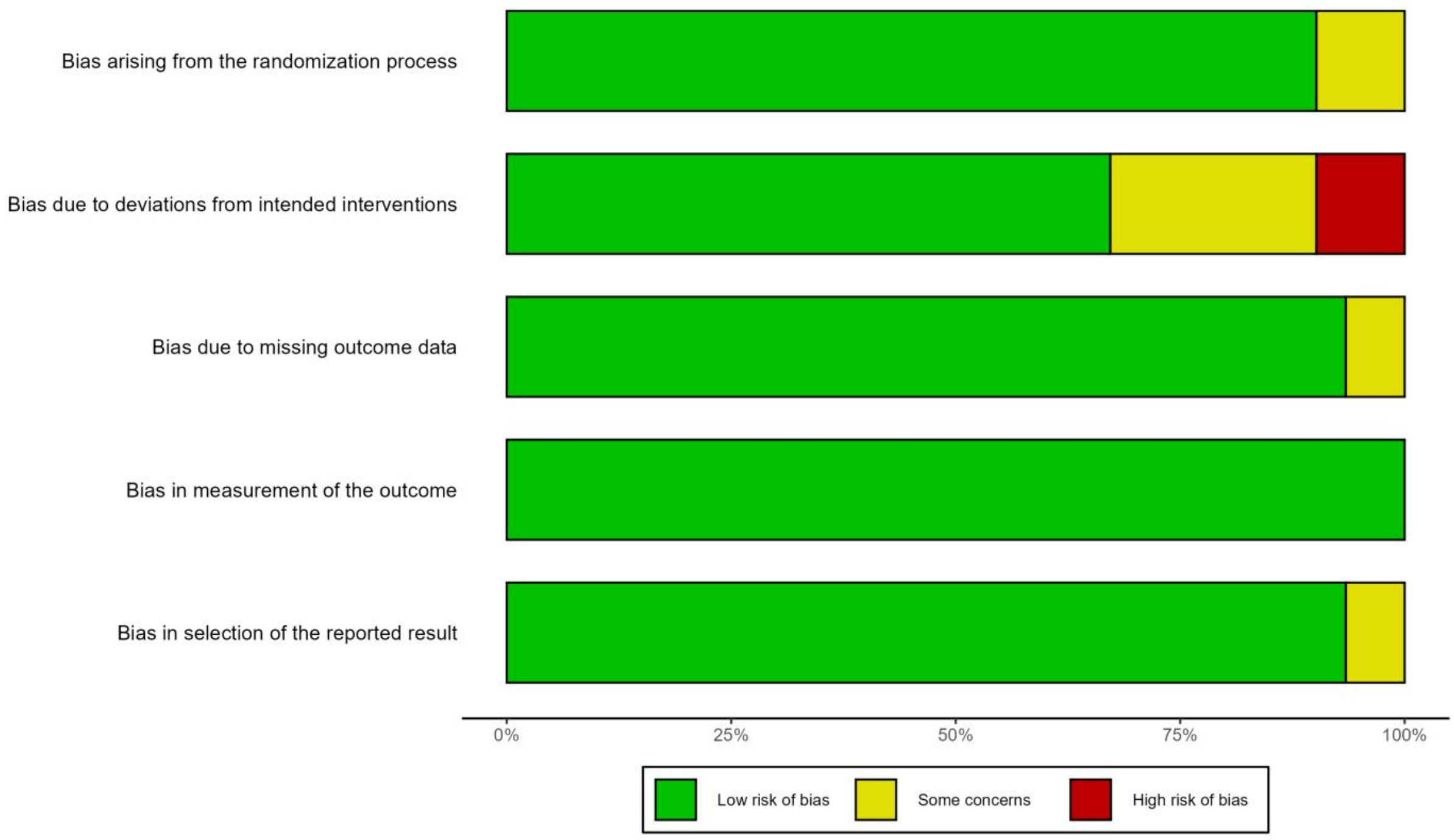

Figure S4. The summary of risk of bias.

6. Network Meta-Analysis Main Results

Table S5. Random-effects treatment estimates vs. CG and SUCRA rankings

Treatment	MD vs CG	95% CrI	SUCRA	Rank
BFR	3.93	[0.60, 7.27]	0.857	1
HIIT	2.71	[1.51, 3.93]	0.726	2
IHT	2.74	[0.60, 4.84]	0.717	3
SSG	2.56	[0.31, 4.79]	0.681	4
SIT	2.49	[1.25, 3.75]	0.676	5
POL	2.28	[-3.79, 8.41]	0.598	6
THR	0.49	[-5.32, 6.49]	0.355	7
RT	0.82	[-0.88, 2.55]	0.352	8
HVT	0.48	[-4.16, 5.11]	0.340	9
AE	0.33	[-1.49, 2.16]	0.256	10
MICT	0.16	[-2.66, 2.91]	0.253	11
CG	—	—	0.191	12

Heterogeneity: tau = 1.22 (posterior mean). Five treatments significantly superior to CG: BFR, HIIT, IHT, SIT, SSG (95% CrI excludes zero).

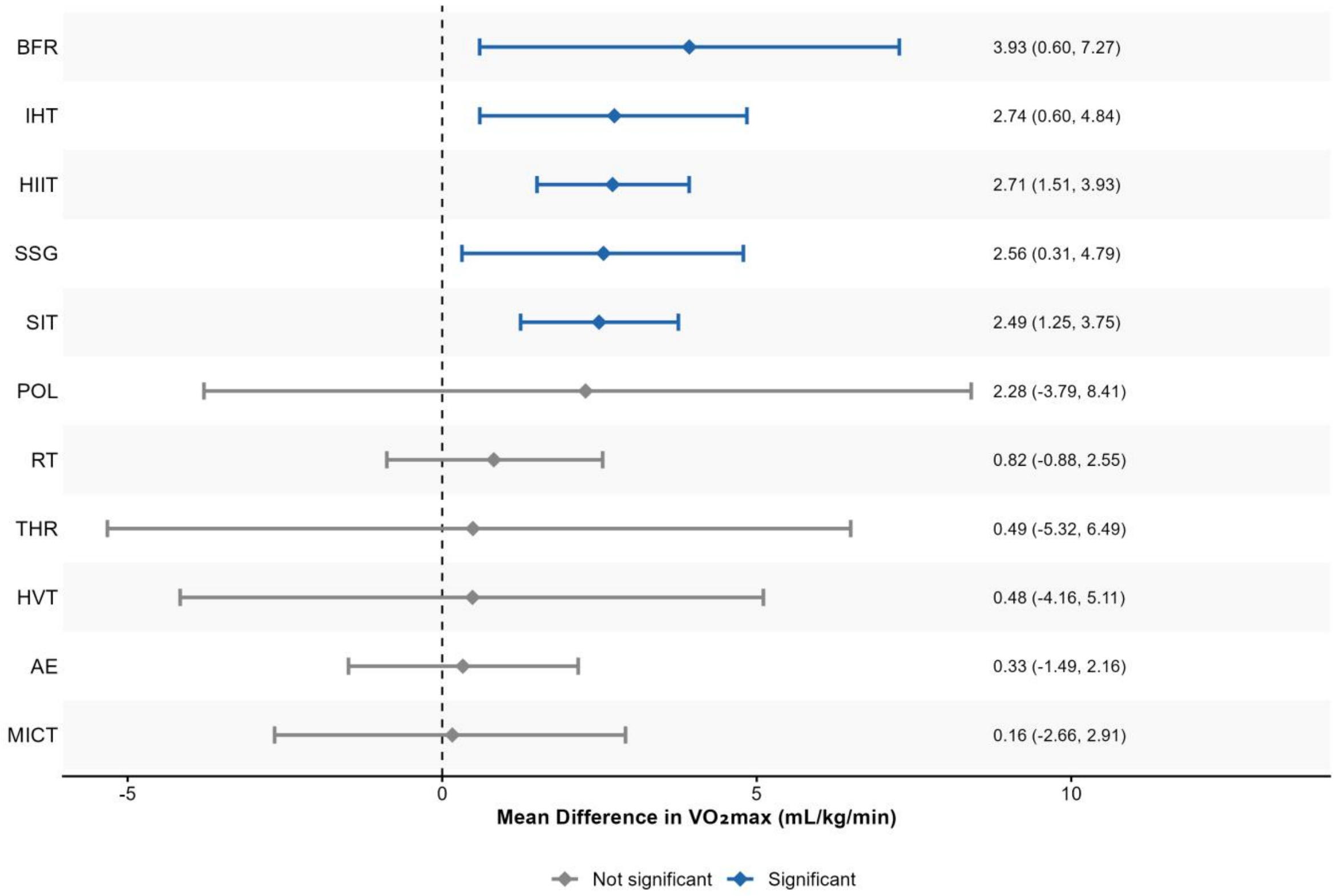

Figure S5. Forest plot

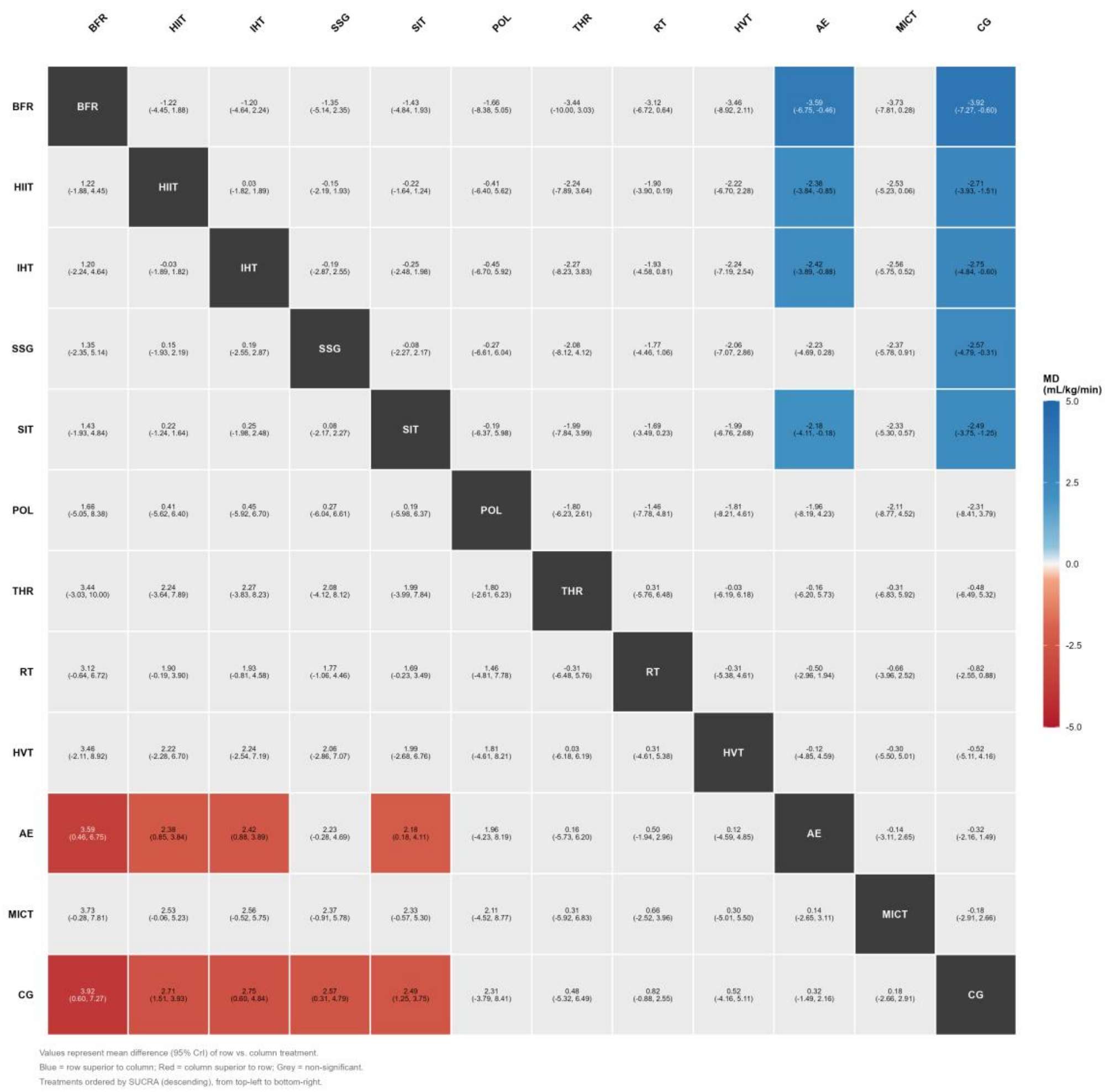

Figure S6. League plot

7. Convergence Diagnostics

All model parameters achieved R-hat < 1.05 (range: 1.000–1.004), confirming adequate convergence of all four MCMC chains.

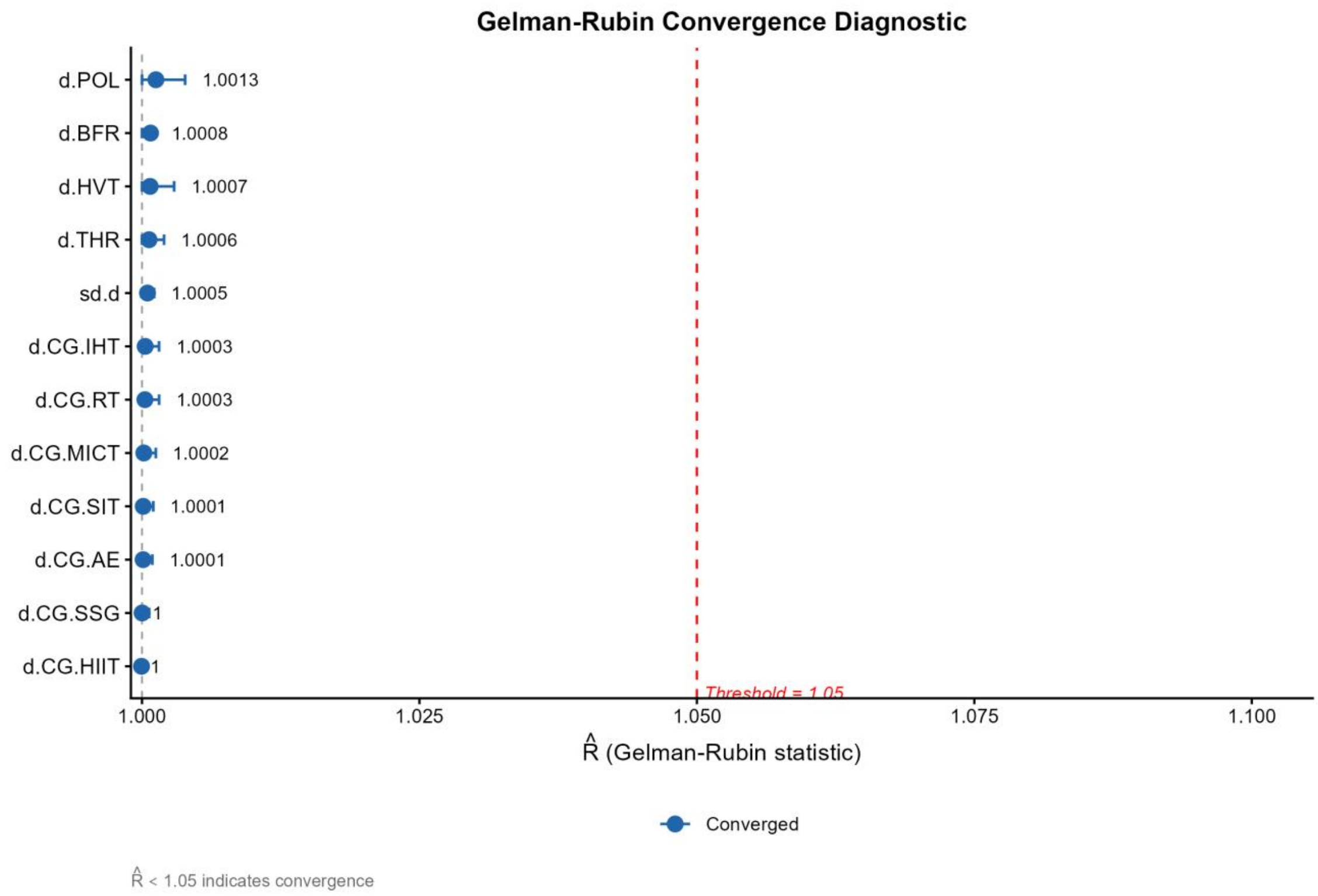

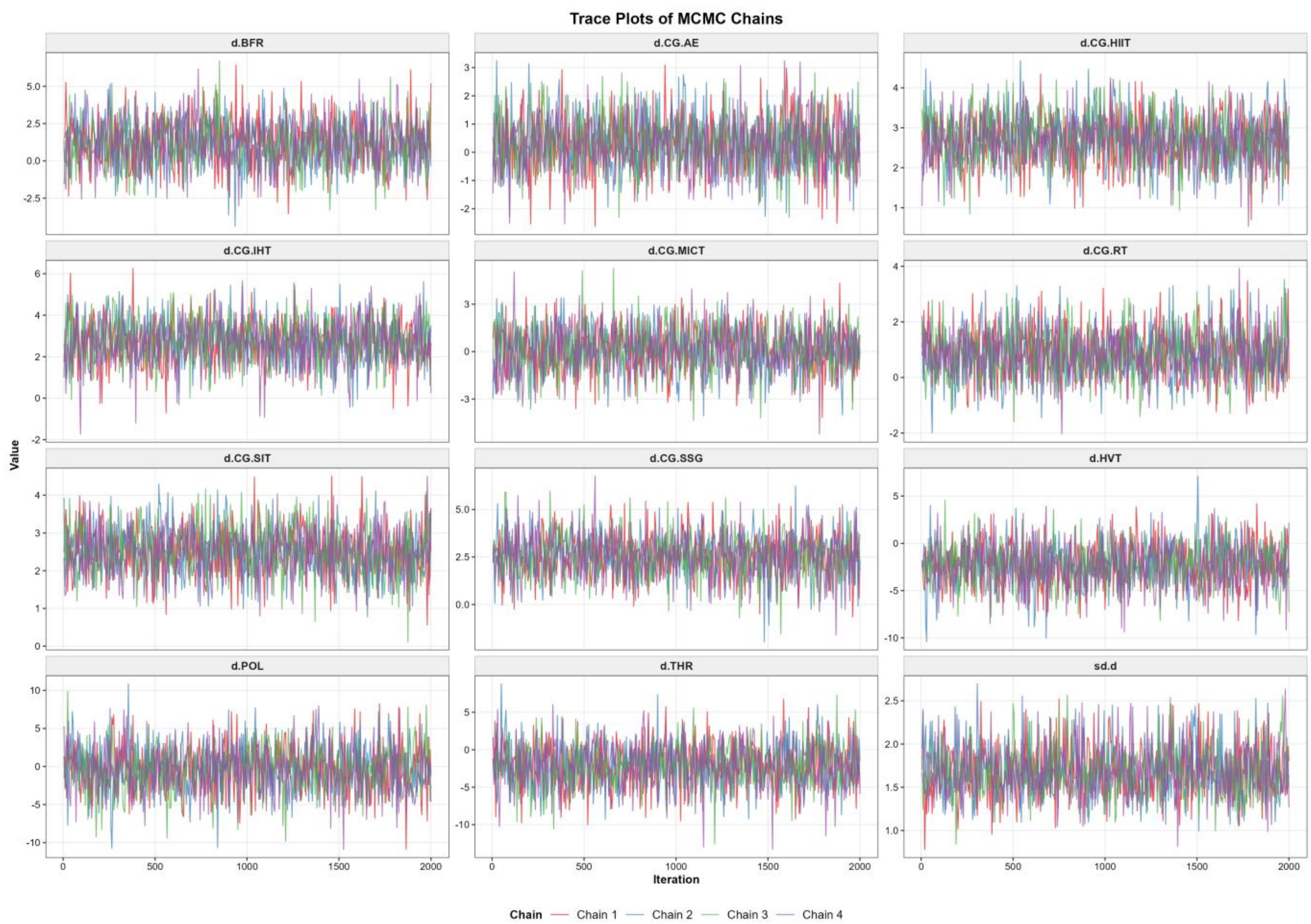

Figure S7. Convergence diagnostic plots

8.Inconsistency Assessment

DIC comparison: Consistency model DIC = 99.37; UME model DIC = 98.39; Difference = -0.98 (< 5, indicating good global consistency).

Table S6. Node-splitting results — significantly inconsistent comparisons ($p < 0.05$).

Comparison	Direct MD [95% CrI]	Indirect MD [95% CrI]	Network MD [95% CrI]	p-value	Assessment
AE vs IHT	1.58 [-0.01, 3.09]	5.84 [2.64, 8.89]	2.40 [0.84, 3.89]	0.023	Inconsistent
HIIT vs IHT	2.49 [-0.56, 5.37]	-1.30 [-3.42, 0.85]	0.00 [-1.86, 1.89]	0.050	Inconsistent

Table S7. (continued). Node-splitting — consistent comparisons ($p \geq 0.05$).

Comparison	Direct MD [95% CrI]	Indirect MD [95% CrI]	Network MD [95% CrI]	p-value	Assessment
AE vs HIIT	3.29 [1.48, 4.98]	0.48 [-1.92, 3.05]	2.41 [0.89, 3.86]	0.085	Consistent
RT vs SIT	0.82 [-1.80, 3.43]	2.81 [-0.07, 5.56]	1.67 [-0.24, 3.52]	0.309	Consistent
CG vs RT	0.48 [-1.40, 2.38]	3.52 [-2.15, 9.12]	0.81 [-0.90, 2.54]	0.314	Consistent
CG vs SSG	5.29 [-0.88, 11.59]	2.19 [-0.25, 4.50]	2.54 [0.35, 4.77]	0.352	Consistent
CG vs IHT	5.79 [-1.67, 13.30]	2.47 [0.19, 4.68]	2.71 [0.56, 4.81]	0.400	Consistent
BFR vs HIIT	-1.16 [-4.99, 2.69]	-2.68 [-9.73, 4.30]	-1.22 [-4.28, 1.86]	0.717	Consistent
AE vs BFR	3.30 [-0.85, 7.40]	4.32 [-1.13, 9.74]	3.63 [0.51, 6.75]	0.773	Consistent
SIT vs SSG	-0.45 [-3.92, 3.21]	0.17 [-2.90, 3.09]	0.06 [-2.13, 2.28]	0.791	Consistent
HIIT vs MICT	-1.64 [-4.71, 1.45]	-2.41 [-8.25, 3.33]	-2.54 [-5.19, 0.08]	0.819	Consistent
HIIT vs SIT	-0.04 [-2.33, 2.29]	-0.34 [-2.20, 1.53]	-0.21 [-1.65, 1.21]	0.835	Consistent
AE vs SIT	2.70 [-3.74, 9.24]	2.08 [-0.03, 4.14]	2.18 [0.19, 4.12]	0.842	Consistent
CG vs HIIT	2.52 [1.09, 3.91]	2.80 [0.36, 5.31]	2.71 [1.46, 3.91]	0.851	Consistent
CG vs SIT	2.56 [1.03, 4.02]	2.34 [-0.08, 4.76]	2.48 [1.23, 3.72]	0.860	Consistent
AE vs CG	-0.56 [-6.10, 5.00]	-0.30 [-2.26, 1.61]	-0.31 [-2.09, 1.50]	0.935	Consistent
AE vs MICT	0.06 [-5.59, 5.61]	-0.17 [-3.59, 3.17]	-0.13 [-3.14, 2.73]	0.950	Consistent
CG vs MICT	-1.01 [-5.88, 3.85]	-1.10 [-4.50, 2.38]	0.17 [-2.62, 2.94]	0.975	Consistent
HIIT vs SSG	-0.17 [-2.66, 2.42]	-0.15 [-3.68, 3.61]	-0.16 [-2.24, 1.96]	0.995	Consistent

BFR, HIIT, and SIT demonstrated robust significant effects vs. CG across both sensitivity analyses. IHT was conditionally significant (present in the primary analysis and Sensitivity 1, absent by design from Sensitivity 2). SSG lost statistical significance when IHT interventions were excluded (Sensitivity 2), suggesting its effect estimate is partly dependent on indirect evidence routed through IHT nodes in the network.

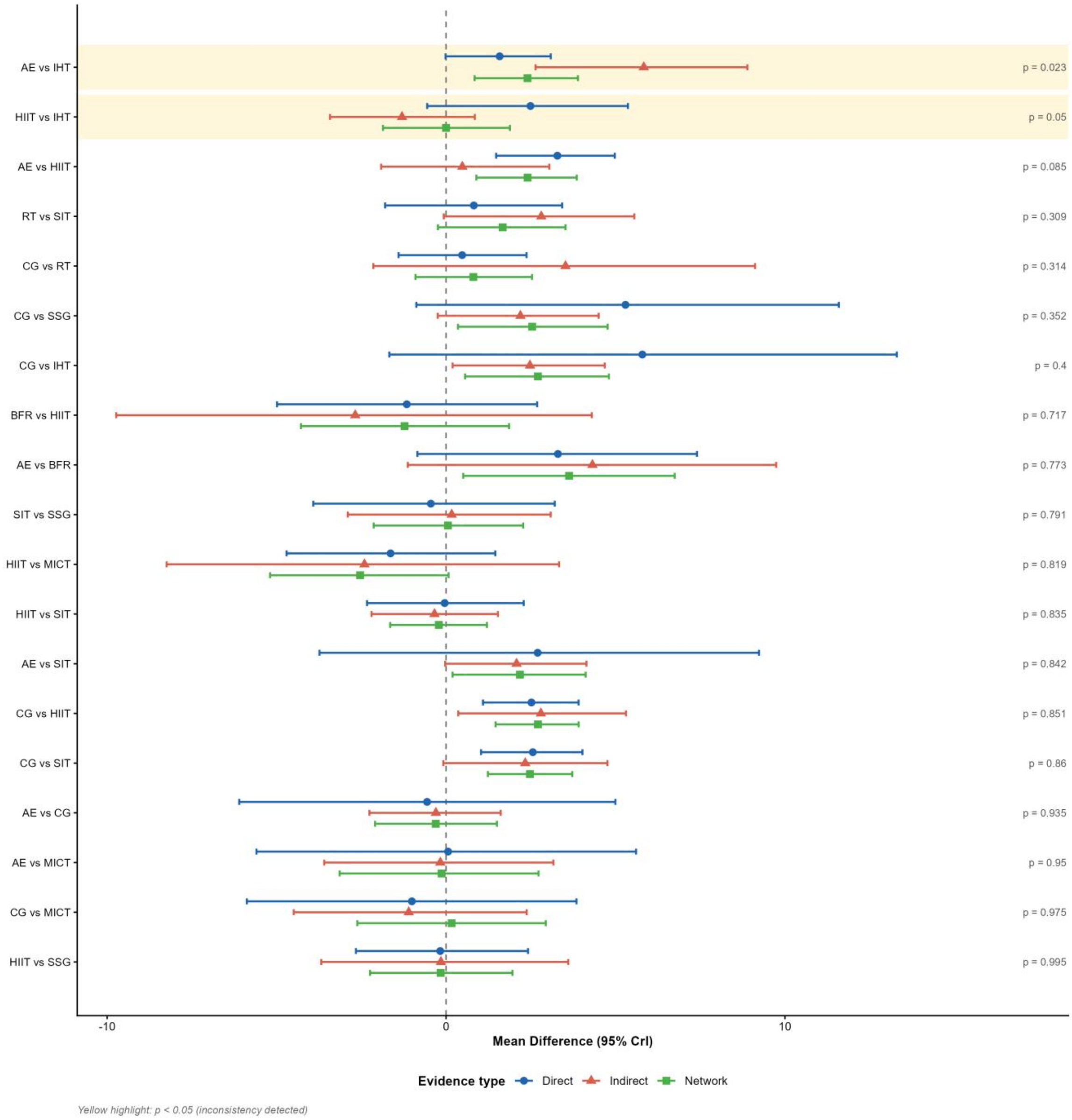

Figure S8. Node-splitting plot

9.Sensitivity Analyses

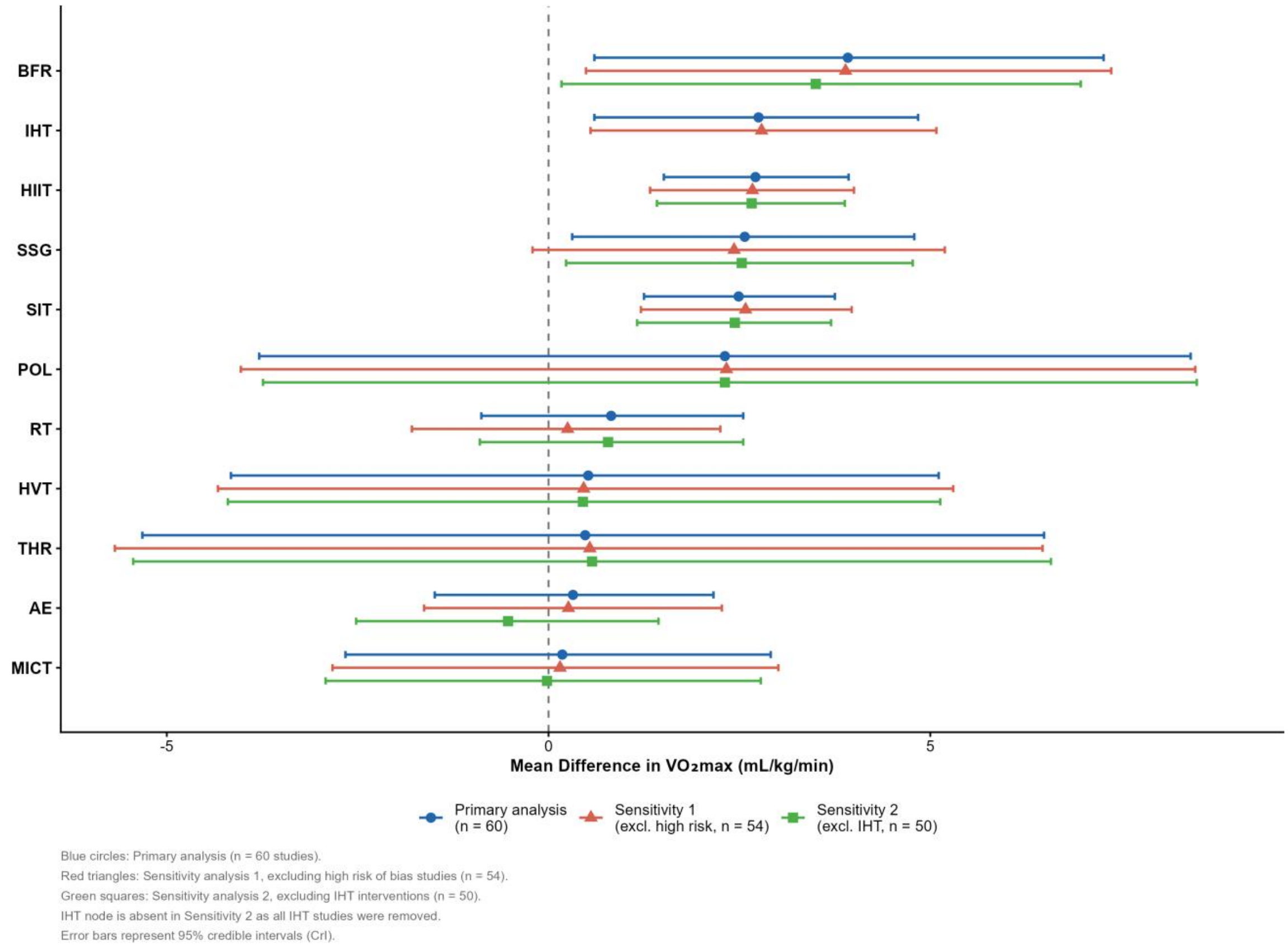

Figure S9. Sensitivity analysis summary

Figure S9 summarizes the primary analysis and both sensitivity analyses: Blue circles = primary analysis (n = 60); Red triangles = Sensitivity 1, excluding high risk of bias studies (n = 54); Green squares = Sensitivity 2, excluding IHT interventions (n = 50).

Table S8. Robustness assessment of treatments with significant effects vs. CG.

Treatment vs CG	Primary (n=60)	Sens.1: No high RoB (n=54)	Sens.2: No IHT (n=50)	Robust?
BFR	3.92 [0.60, 7.27] ✓	✓	✓	Yes
HIIT	2.71 [1.51, 3.93] ✓	✓	✓	Yes
SIT	2.49 [1.25, 3.75] ✓	✓	✓	Yes
IHT	2.75 [0.60, 4.84] ✓	✓	— (excluded)	Conditional
SSG	2.57 [0.31, 4.79] ✓	✓	Crosses zero	Unstable

Note: SSG lost statistical significance when IHT interventions were excluded (Sensitivity 2), suggesting its effect estimate is partly dependent on indirect evidence routed through IHT nodes in the network.

10. Network Meta-Regression

Table S9. Network meta-regression (age, baseline VO2max, intervention duration)

Covariate	DIC	Residual deviance	Shared beta (median and 95%CrI)	SD
Age (years)	219.9	122.5	-1.09 (-3.04, 0.91)	1.65 (1.13, 2.29)
Baseline VO2max (mL/kg/min)	220.3	122.6	-1.22 (-2.98, 0.57)	1.63 (1.12, 2.29)
Intervention length (weeks)	221.3	123.2	0.42 (-1.19, 1.96)	1.67 (1.15, 2.36)

Note: DIC: deviance information criterion; SD: standard deviation

11. Subgroup Analysis by Sport Type

Table S10. SUCRA rankings — Endurance subgroup (25 studies, Bayesian NMA).

Rank	Treatment	SUCRA
1	IHT	0.821
2	BFR	0.671
3	MICT	0.623
4	SIT	0.557
5	CG	0.508
6	POL	0.448
7	RT	0.437
8	AE	0.423
9	HIIT	0.416
10	HVT	0.338
11	THR	0.258

No treatment showed statistically significant difference vs. CG (all 95% CrI cross zero). All 12 node-splitting comparisons consistent. Tau = 1.22.

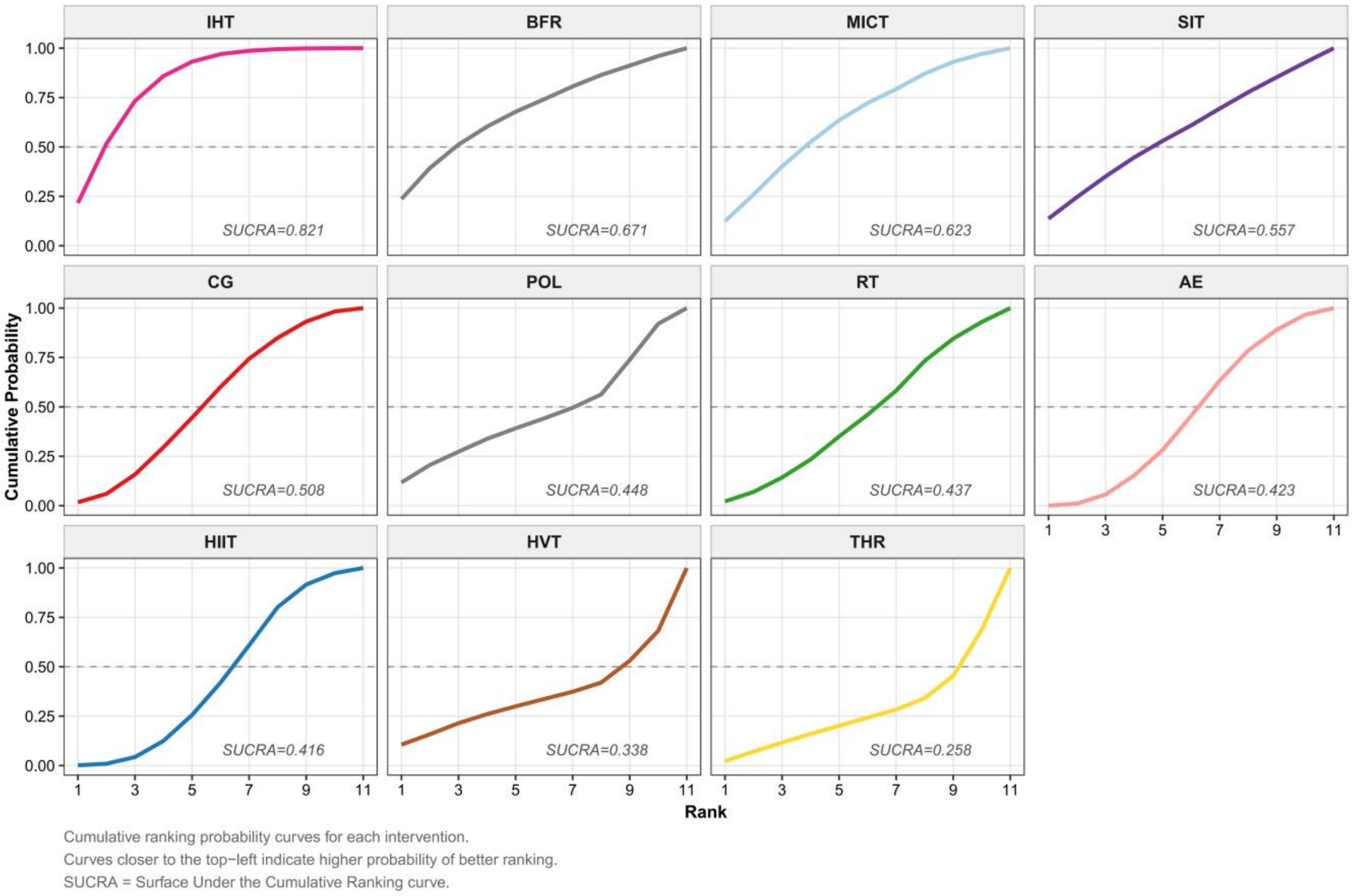

Figure S10. SUCRA rankings plot

Table S11. Pairwise meta-analysis vs. CG — subgroups with sparse networks (random-effects, DerSimonian-Laird).

Subgroup	Treatment	k	MD [95% CI]	I²	p-value
Team Ball (18)	SIT	5	2.70 [1.17, 4.23]	0%	0.001
	RT	2	2.90 [0.69, 5.11]	0%	0.010
	HIIT	4	2.85 [-1.57, 7.28]	1%	0.207
	SSG	1	5.30 [0.00, 10.60]	—	0.050
Water Sports (7)	HIIT	2	4.69 [1.76, 7.62]	0%	0.002
	SIT	1	2.50 [-0.87, 5.87]	—	0.145
Combat/Racket (10)	SIT	5	2.59 [1.20, 3.99]	0%	<0.001
	HIIT	3	2.46 [0.75, 4.17]	0%	0.005
	RT	1	-0.30 [-2.88, 2.28]	—	0.820

k = number of studies with direct CG comparison. BFR, AE, HVT, and MICT had no direct CG comparisons in these subgroups. SIT and HIIT were the only treatments with consistent, statistically significant benefits vs. CG across multiple sport subgroups.

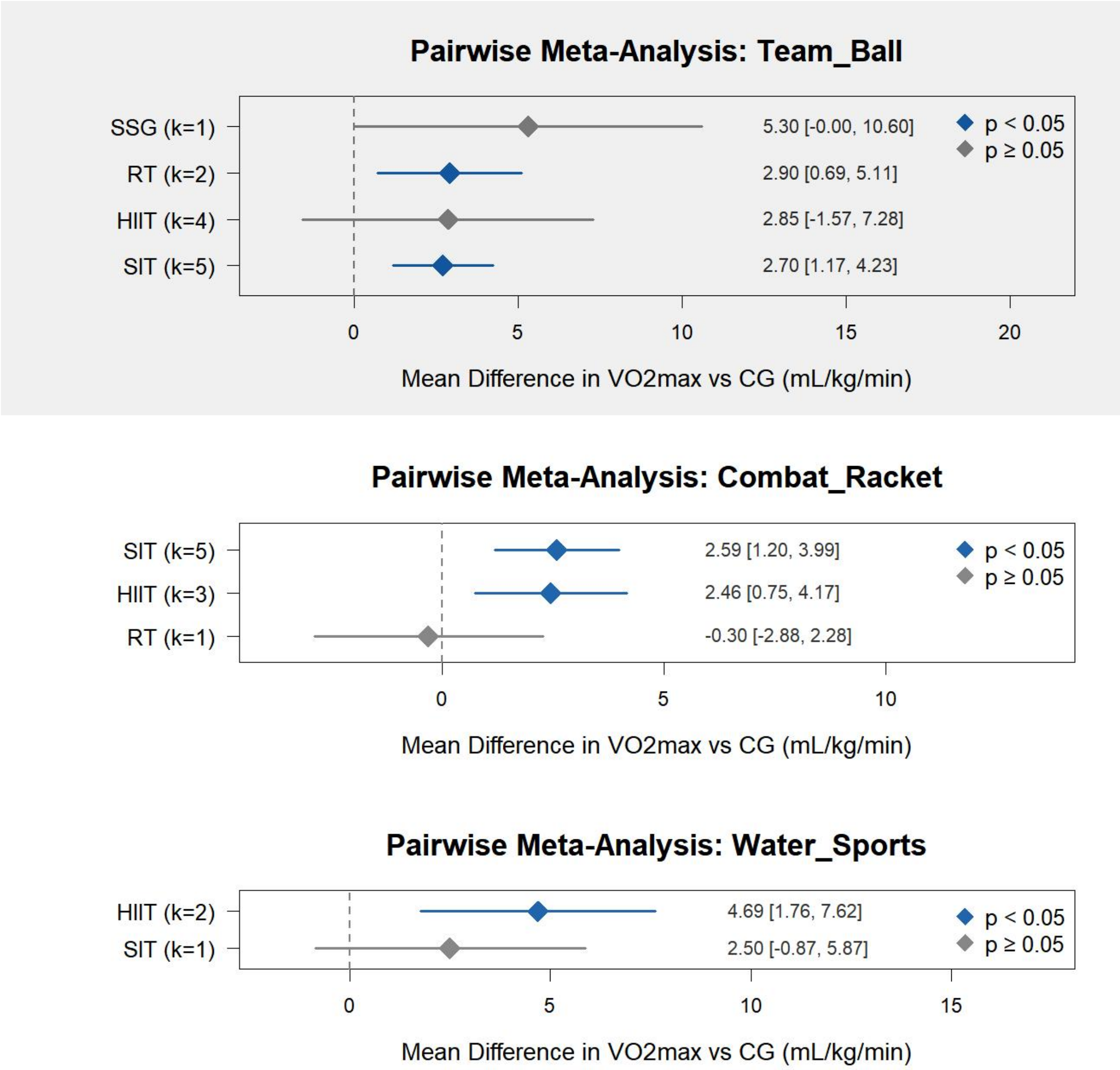

Figure S11. Forest plots

12. Publication Bias

Publication bias was assessed using a comparison-adjusted funnel plot with Egger's regression test.

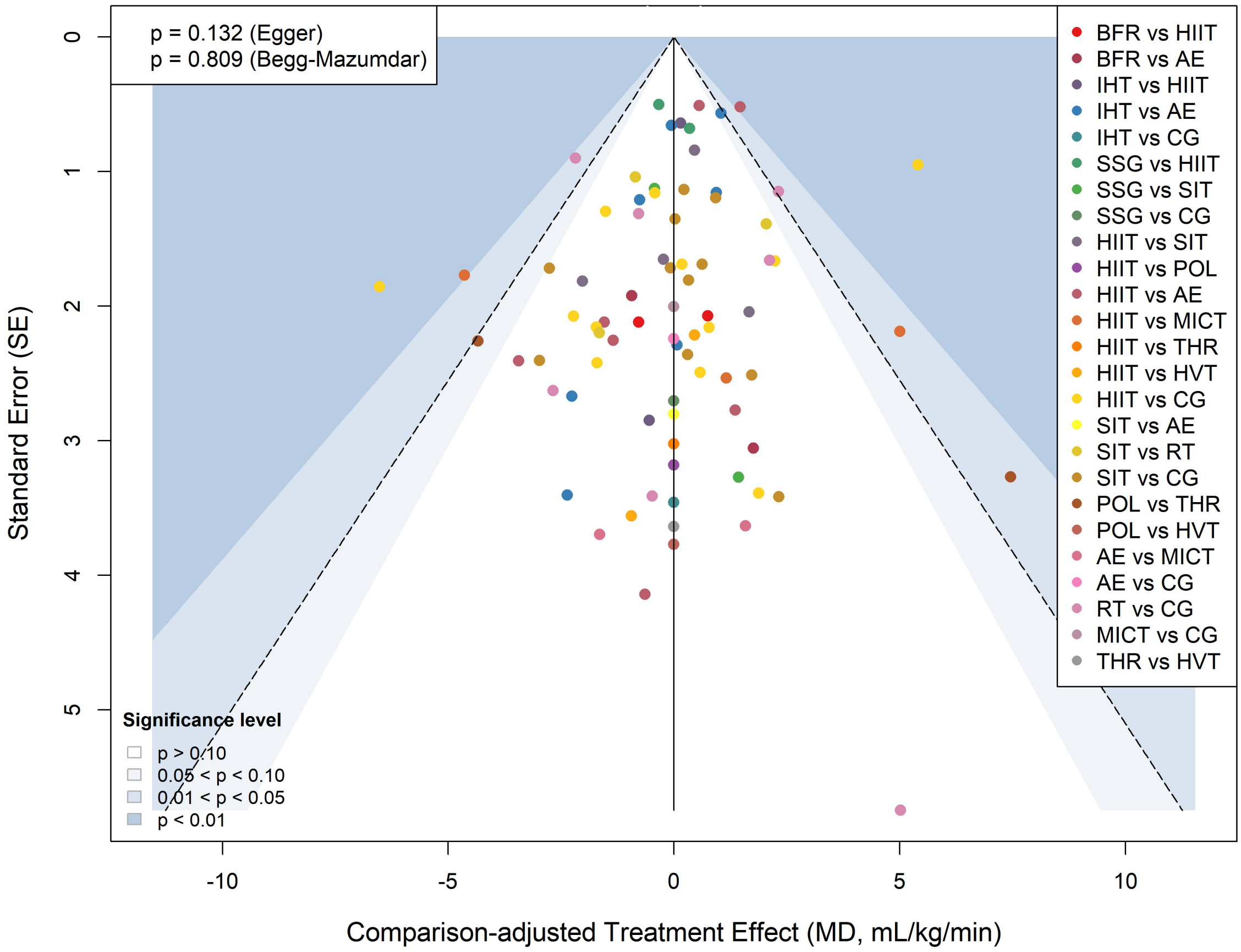

Figure S12. Comparison-adjusted funnel plot

13.Grade summary of all studies

Based on the CINeMA online guidelines (https://cinema.ispm.unibe.ch/), we evaluated the confidence of evidence for the maximal oxygen uptake (VO_2max) outcome:

13.1 Within-study Bias

Each included study was rated for risk of bias as low (RoB2 1 point), moderate (RoB2 2 points), or high (RoB2 3 points). The rule applied was the average RoB. No downgrade occurred for "no concerns"; one level was downgraded for "some concerns," and two levels for "major concerns."

13.2 Reporting Bias (Publication Bias and Selective Reporting)

To minimize publication bias, we examined the reference lists of recent relevant studies. Publication bias was assessed using comparison-adjusted funnel plots, Begg's and Egger's test (Section 12; Figure S12). The results indicated no statistically significant bias ($p > 0.05$), suggesting a low risk of publication bias. Furthermore, no high risk of selective reporting was identified; therefore, this domain was not downgraded.

13.3 Indirectness

Indirectness assessment was conducted by analyzing the clinical and methodological relevance of the included studies to the research question. All included studies strictly adhered to the predefined PICOS criteria: they involved healthy athletes, employed various exercise training modalities, established appropriate control groups (maintaining baseline training), and used maximum oxygen uptake (VO_2 max) as the primary outcome measure. Although variations in specific sports disciplines and baseline cardiorespiratory fitness levels across studies may introduce some indirectness, this was effectively addressed through predefined subgroup analyses (e.g., endurance vs. team sports) and rigorous sensitivity analyses.

13.4 Imprecision

In this network meta-analysis, maximum oxygen uptake (VO_2 max) was analyzed as a continuous variable. Because VO_2 max is universally reported in standardized units (mL/kg/min) across the included trials, the mean difference (MD) was employed as the effect size indicator, calculated via change scores (endpoint minus baseline values). Recognizing the lack of a universally established minimum clinically important difference (MCID) for VO_2 max improvements across diverse athletic disciplines, the null value (0 mL/kg/min) was established as the absolute reference threshold to evaluate the efficacy of the exercise interventions.

13.5 Heterogeneity

Beyond statistical heterogeneity, physiological and methodological heterogeneity arising from variations in baseline cardiorespiratory fitness, specific athletic disciplines, foundational training loads, and the standardization of exercise interventions was rigorously evaluated when assigning heterogeneity ratings. Comparisons exhibiting prediction intervals that extended substantially beyond their corresponding credible intervals—indicating variability of practical relevance to athletic training prescription—were downgraded accordingly, as detailed in the individual comparison assessments in Table S12.

13.6 Incoherence

Incoherence was addressed by p-value thresholds: no downgrade for $p > 0.1$, one level for p 0.05–0.1, and two levels for $p < 0.05$.

13.7 Summarizing Judgments Across CINeMA Domains

The CINeMA framework assesses confidence in evidence across six domains: within-study bias, reporting bias, indirectness, imprecision, heterogeneity, and incoherence. These judgments are summarized into an overall confidence rating using the GRADE approach (high, moderate, low, or very low). As a single issue may impact multiple domains (e.g., indirectness due to intransitivity or heterogeneity affecting both imprecision and bias), all domains are considered together to avoid redundant downgrading and ensure a balanced assessment.

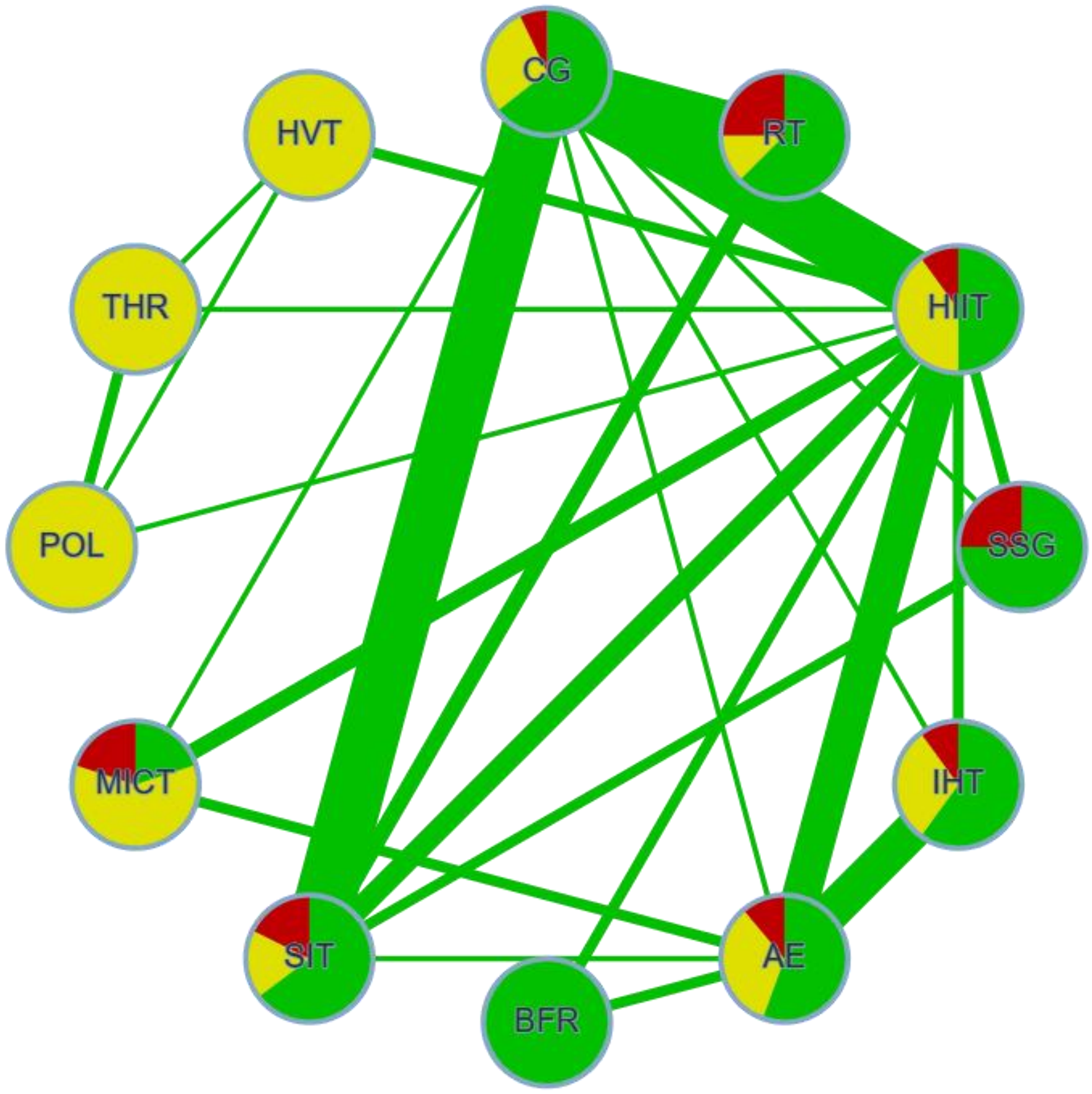

Figure S13. Network plot of study limitations of the included studies

The colors in the circles indicate the percentage of low RoB studies (green), moderate RoB studies (yellow), high RoB studies (red) about each physical activity type. Edge width by the number of studies. Edge color by average RoB. The colors of the lines indicate the summative RoB assessment of each comparison. Low RoB is green, moderate RoB is yellow, high RoB is red.

Table S12. The confidence of evidence using the CINeMA

Comparison	Number of studies	Within-study bias	Reporting bias	Indirectness	Imprecision	Heterogeneity	Incoherence	Confidence rating	Reason (s) for downgrading
AE:BFR	2	No concerns	Low risk	No concerns	No concerns	Major concerns	No concerns	Low	["Heterogeneity"]
**AE:CG**	1	Some concerns	Low risk	No concerns	Major concerns	No concerns	No concerns	**Very low**	["Within-study bias","Imprecision"]
AE:HIIT	7	Some concerns	Low risk	No concerns	No concerns	Major concerns	No concerns	Low	["Within-study bias","Heterogeneity"]
AE:IHT	7	No concerns	Low risk	No concerns	No concerns	Major concerns	Major concerns	Very low	["Heterogeneity","Incoherence"]
AE:MICT	2	Some concerns	Low risk	No concerns	Major concerns	No concerns	No concerns	Very low	["Within-study bias","Imprecision"]
AE:SIT	1	Some concerns	Low risk	No concerns	No concerns	Major concerns	No concerns	Very low	["Within-study bias","Heterogeneity"]
BFR:HIIT	2	No concerns	Low risk	No concerns	Major concerns	No concerns	No concerns	Low	["Imprecision"]
**HIIT:CG**	12	No concerns	Low risk	No concerns	No concerns	Major concerns	No concerns	**Low**	["Heterogeneity"]
**IHT:CG**	1	Some concerns	Low risk	No concerns	No concerns	Major concerns	No concerns	**Very low**	["Within-study bias","Heterogeneity"]
**MICT:CG**	1	Some concerns	Low risk	No concerns	Major concerns	No concerns	No concerns	**Very low**	["Within-study bias","Imprecision"]
**RT:CG**	7	No concerns	Low risk	No concerns	Major concerns	No concerns	No concerns	**Low**	["Imprecision"]
**SIT:CG**	11	No concerns	Low risk	No concerns	No concerns	Major concerns	No concerns	**Low**	["Heterogeneity"]
**SSG:CG**	1	No concerns	Low risk	No concerns	No concerns	Major concerns	No concerns	**Low**	["Heterogeneity"]
HIIT:HVT	2	Some concerns	Low risk	No concerns	Major concerns	No concerns	Major concerns	Very low	["Within-study bias","Imprecision","Incoherence"]
HIIT:IHT	2	Some concerns	Low risk	No concerns	Major concerns	No concerns	No concerns	Very low	["Within-study bias","Imprecision"]

Comparison	Number of studies	Within-study bias	Reporting bias	Indirectness	Imprecision	Heterogeneity	Incoherence	Confidence rating	Reason (s) for downgrading
HIIT:MICT	3	Some concerns	Low risk	No concerns	Major concerns	No concerns	No concerns	Very low	["Within-study bias","Imprecision"]
HIIT:POL	1	Some concerns	Low risk	No concerns	Major concerns	No concerns	No concerns	Very low	["Within-study bias","Imprecision"]
HIIT:SIT	4	Some concerns	Low risk	No concerns	Major concerns	No concerns	No concerns	Very low	["Within-study bias","Imprecision"]
HIIT:SSG	2	Some concerns	Low risk	No concerns	Major concerns	No concerns	No concerns	Very low	["Within-study bias","Imprecision"]
HIIT:THR	1	Some concerns	Low risk	No concerns	Major concerns	No concerns	No concerns	Very low	["Within-study bias","Imprecision"]
HVT:POL	1	Some concerns	Low risk	No concerns	Major concerns	No concerns	No concerns	Very low	["Within-study bias","Imprecision"]
HVT:THR	1	Some concerns	Low risk	No concerns	Major concerns	No concerns	No concerns	Very low	["Within-study bias","Imprecision"]
POL:THR	2	Some concerns	Low risk	No concerns	Major concerns	No concerns	Major concerns	Very low	["Within-study bias","Imprecision","Incoherence"]
RT:SIT	3	No concerns	Low risk	No concerns	Major concerns	No concerns	No concerns	Low	["Imprecision"]
SIT:SSG	2	No concerns	Low risk	No concerns	Major concerns	No concerns	No concerns	Low	["Imprecision"]

14.Dose-Response Analysis

14.1 Quantification of Training Dose

Due to heterogeneity in exercise intensity reporting across included RCTs—ranging from absolute mechanical workload to relative physiological thresholds (%HRmax, %VO_2max) or qualitative descriptors ("all-out")—a hierarchical data extraction strategy was developed to standardize the quantification of training dose in METs.

Tier 1: Direct matching. For studies reporting absolute mechanical workload (e.g., power output in watts, absolute speed), the precise MET value was assigned using the 2024 Compendium of Physical Activities [63].

Tier 2: Standardized MET anchoring matrix. For studies reporting only relative intensity, the following standardized MET values were applied. Baseline values were derived from the 2024 Compendium, with intensity-specific boundaries calibrated against consensus thresholds from exercise physiology literature.

Table S13. Standardized MET assignment rules for dose-response modelling

Training Modality	Intensity Description / Physiological Anchor	Assigned MET	Key Reference
SIT (Sprint Interval Training)	All-out / Supramaximal (>100% VO_2max)	15.0	Gibala et al [64].
HIIT (High-Intensity Interval Training)	90–95% HRmax / Severe intensity domain	12.0	Buchheit & Laursen [65]
AE (High-Intensity Aerobic Exercise)	80–90% HRmax	5.5	Seiler [66]; ACSM [67].
SSG (Small-Sided Games)	75–85% HRmax / Sport-specific	9.5	Hill-Haas et al [68].
MICT (Moderate-Intensity Continuous Training)	70–80% HRmax	8.0	Seiler [66]; ACSM [67].
RT (Heavy Resistance Training)	≥80% 1RM	6.0	ACSM [69].
CG (Control Group)	Usual training maintenance	0	—

Note: MET values were used as external-dose anchors for harmonizing heterogeneous supplementary training interventions, not as measured internal physiological load. When studies reported absolute workload or directly matchable activities, values were matched to the 2024 Adult Compendium of Physical Activities. When studies reported relative intensity descriptors, modality-level anchors were applied using established exercise-physiology references. For supramaximal SIT and resistance-based exercise, MET-based scaling may underestimate internal physiological stimulus, anaerobic contribution, neuromuscular demand, and recovery cost. Study-arm-level dose values used in the model are reported in Supplementary Table S19. MET, metabolic equivalent of task; SIT, sprint interval training; HIIT, high-intensity interval training; AE, vigorous aerobic exercise; SSG, small-sided games; MICT, moderate-intensity continuous training; RT, resistance training; CG, control group; HRmax, maximum heart rate; 1RM, one-repetition maximum. Assigned MET values were calibrated against the 2024 Compendium of Physical Activities and corresponding physiological consensus guidelines.
The specific dose calculation method is detailed in Section 14.3.

14.2 Note on SIT Dose Characteristics

In the dose-response models, SIT sometimes showed a relatively low computed MET-min/week dose with favourable estimated effects. This should not be interpreted as a low physiological stimulus. MET-min/week is an external exposure metric and may underrepresent supramaximal exercise because it does not fully capture anaerobic contribution, neuromuscular demand, or recovery cost.

14.3 Standardized MET assignment rules for supplementary training modalities.

A subset of 31 studies with quantified supplementary dose data was analyzed using a Bayesian dose-response NMA with the quadratic dose-response model selected by DIC (Section 14.7). Weekly dose (METs-min/week) was calculated as METs × session duration × frequency ×average adherence rate, representing net additional training beyond regular sport-specific practice.

- **Agent:** The agent level.
- **MET_min:** The energy expenditure per min in the study.
- **Min_session：** The duration of a single exercise intervention.
- **Frequency:** The number of days participants engaged in physical activity.
- **Exact_dose:** The exact estimated METs accumulated per week by participants.
- **Residual_dose:** The difference between the exact dose and the approximated dose.
- **Dose:** The dose group by approximation.
- **CG:** Control group

studyID	Agent	MET_min	min_session	MET_session	frequency	exact_dose	residual_dose	dose	Average adherence rate (%)	n
1	RT	3.5	90	315	2	504	4	500	80%	20
1	CG	0	0	0	0	0	0	0	0	20

Note: MET values were assigned using a two-step approach. When studies reported absolute workload or directly matchable activities, values were matched to the 2024 Adult Compendium of Physical Activities. When studies reported relative intensity descriptors, standardized modality-level anchors were assigned using established exercise-physiology references. These values were used to calculate weekly supplementary dose as MET value × session duration × weekly frequency × adherence.

Table S19. Study-arm-level data used in the dose-response network meta-analysis.

Study	Study ID	Agent	Model dose (MET-min/week)	Original dose (MET-min/week)	Baseline VO2max	Change (y)	SD	SE	n	Length (weeks)	Age	Country/Region	Sport	Sex
aagaard et al.2011	1	RT	700	700	73.5（8.2）	1.50	7.35	2.78	7	16	19.5(0.8)	Denmark	cyclists	Male
aagaard et al.2011	1	CG	0	0	71.5（6.0）	1.50	5.24	1.98	7	16	19.5(0.8)	Denmark	cyclists	Male
Bicer et al.2020	2	RT	750	740	37.85（4.34）	4.65	7.70	2.14	13	8	27.08(3.99)	Turkey	volleyball	Male
Bicer et al.2020	2	CG	0	0	51.81（19.03）	-0.85	19.24	5.34	13	8	27.46(4.05)	Turkey	volleyball	Male
Bilici et al.2026	3	HIIT	550	550	37.40(4.86)	0.90	4.87	1.54	10	12	16.00(1.05)	Turkey	volleyball	Female
Bilici et al.2026	3	HIIT	650	650	36.73(2.37)	1.45	2.54	0.77	11	12	16.18(1.17)	Turkey	volleyball	Female
Bilici et al.2026	3	CG	0	0	37.82(3.49)	0.18	3.35	1.01	11	12	16.64(1.29)	Turkey	volleyball	Female
Fernandez et al.2012	4	HIIT	500	486	56.3(4.0)	2.80	3.58	1.08	11	6	22.6(4.8)	Germany	tennis	Male
Fernandez et al.2012	4	SIT	150	120	55.6(5.0)	3	4.35	1.26	12	6	21.2(5.1)	Germany	tennis	Male
Fernandez et al.2012	4	CG	0	0	57.3(4.0)	0.10	3.90	1.30	9	6	22.1(3.3)	Germany	tennis	Male
Gantois et al.2022	5	SIT	50	52.70	50.39(4.30)	1.91	4.64	1.47	10	6	21.6(3.34)	Brazil	volleyball	Male
Gantois et al.2022	5	CG	0	0	48.84(5.89)	-0.97	5.55	1.85	9	6	21.33(2.91)	Brazil	volleyball	Male
Jarstad et al.2019	6	HIIT	700	700	50.6(5.4)	2.90	6.29	2.38	7	10	28(5)	Norway	endurance athletes	Mix
Jarstad et al.2019	6	MICT	1000	1000	50.9(7.1)	2.40	7.15	2.70	7	10	28(5)	Norway	endurance athletes	Mix
Jarstad et al.2019	6	CG	0	0	54.5(7.3)	1.20	7.73	3.16	6	10	28(5)	Norway	endurance athletes	Mix
Jin et al.2025	7	HIIT	1350	1350	51.48(3.85)	3.55	4.47	1.29	12	4	19.94(1.4)	China	swimmers	Mix
Jin et al.2025	7	CG	0	0	49.40(3.75)	-1.21	3.65	1.05	12	4	21.17(1.6)	China	swimmers	Mix
Jin et al.2025	8	SIT	150	150	67.2（5.0）	2.90	5.38	1.70	10	6	19（1）	China	distance runners	Male
Jin et al.2025	8	AE	700	720	68.8（7.0）	0.10	7.05	2.23	10	6	20(1)	China	distance runners	Male
kaynak et al.2017	9	SIT	50	34.10	50(3.6)	3.40	3.12	1.04	9	6	21.2(1.3)	Turkey	volleyball	Male
kaynak et al.2017	9	CG	0	0	50.4(4)	0.20	4	1.33	9	6	21.2(1.6)	Turkey	volleyball	Male
Kim et al.2011	10	SIT	250	250	49.84(4.08)	3.28	4.47	1.35	11	8	20.00(1.10)	Korea	Judo	Male
Kim et al.2011	10	CG	0	0	46.63(4.99)	3.46	4.68	1.07	19	8	19.94(1.16)	Korea	Judo	Male
Kirchenberger et al.2021	11	HIIT	450	448	58.4(3.9)	3.70	3.40	1.07	10	8	15.4(1.3)	Germany	Rowers	Male
Kirchenberger et al.2021	11	MICT	800	800	58.4(5.7)	-0.10	6.08	2.30	7	8	15.3(1.2)	Germany	Rowers	Male
Kristoffersen et al.2019	12	SIT	50	36	63.3（5.9）	1.50	6.06	1.51	16	6	29.6（0.6）	Norway	cyclists	Mix
Kristoffersen et al.2019	12	RT	550	540	61.1（6.0）	2.30	6.38	1.59	16	6	29.6（0.6）	Norway	cyclists	Mix
Liu et al.2023	13	SIT	130	135	54.6（3.6）	3.20	3.42	1.21	8	4	25(6)	Korea	swimmers	Male

Study	Study ID	Agent	Model dose (MET-min/week)	Original dose (MET-min/week)	Baseline VO2max	Change (y)	SD	SE	n	Length (weeks)	Age	Country/Region	Sport	Sex
Liu et al.2023	13	CG	0	0	54.5（3.5）	0.70	3.45	1.22	8	4	25(6)	Korea	swimmers	Male
Maggioni et al.2019	14	SSG	300	288	57.4(6.7)	2.80	8.38	2.42	12	8	19(1)	Italy	Basketball	NR
Maggioni et al.2019	14	SIT	50	65	55.7(7.2)	1.80	6.61	2.20	9	8	19(1)	Italy	Basketball	NR
Maggioni et al.2019	14	CG	0	0	56.7(2.8)	-2.50	3.63	1.21	9	8	19(1)	Italy	Basketball	NR
Mallol et al.2019	15	HIIT	350	352	42.4(5.2)	2.80	5.64	1.99	8	4	42.9(12.1)	Australia	triathletes	Mix
Mallol et al.2019	15	CG	0	0	43.1(4.5)	-0.30	4.23	1.50	8	4	37.2(13.3)	Australia	triathletes	Mix
Millet et al.2002	16	RT	550	540	69.7(3.60)	-2.50	4.06	1.53	7	14	24.3(5.2)	France	triathletes	NR
Millet et al.2002	16	CG	0	0	67.6(6.4)	-0.30	6.04	2.14	8	14	21.4(2.1)	France	triathletes	NR
MonKS et al.2017	17	HIIT	250	240	56.1（1.38）	4.70	1.49	0.37	16	4	18-22	Korea	Taekwondo	Mix
MonKS et al.2017	17	AE	550	550	51.5（1.34）	0.90	1.44	0.35	17	4	18-22	Korea	Taekwondo	Mix
Morais et al.2025	18	HIIT	200	216	45.21(2.06)	3.62	2.18	0.63	12	6	13.6(0.3)	Spain	tennis	Male
Morais et al.2025	18	SIT	50	45	45.50(1.85)	3.13	1.93	0.56	12	6	13.6(0.3)	Spain	tennis	Male
Muñoz et al.2021	19	HIIT	150	154	37.50(6.04)	2.70	5.64	1.78	10	8	23.6（2.8）	Chile	Field Hockey	Female
Muñoz et al.2021	19	CG	0	0	35.25(5.50)	1.88	5.18	1.64	10	8	23.6（3.0）	Chile	Field Hockey	Female
ouergui et al.2020	20	SIT	150	125	50.9(3.4)	4.10	3.18	0.88	13	4	16(1)	Tunisia	Taekwondo	Mix
ouergui et al.2020	20	SSG	100	95	51.1(4.9)	5.30	4.42	1.23	13	4	16(1)	Tunisia	Taekwondo	Mix
ouergui et al.2020	20	CG	0	0	48.3(3.2)	1.20	3.36	0.93	13	4	16(1)	Tunisia	Taekwondo	Mix
ØVRETVEIT et al.2019	21	HIIT	400	384	52.7（5.3）	4.10	5.11	2.09	6	6	30.3(4.0)	Norway	BJJ	Male
ØVRETVEIT et al.2019	21	CG	0	0	53.3（1.2）	0.80	1.37	0.56	6	6	30.3(4.0)	Norway	BJJ	Male
Rønnestad et al.2010	22	RT	400	400	65.2(2.2)	8.70	2.84	1.16	6	25	29(3)	Norway	cyclists	Male
Rønnestad et al.2010	22	CG	0	0	67.3(2.7)	6.10	2.92	1.19	6	25	31(3)	Norway	cyclists	Mix
Rønnestad et al.2010	23	RT	550	540	66.8(1.6)	2.20	1.60	0.48	11	12	27（2）	Norway	cyclists	Male
Rønnestad et al.2010	23	CG	0	0	65.9(2.0)	3.90	2.29	0.76	9	12	30（2）	Norway	cyclists	Mix
Sandbakk et al.2013	24	HIIT	600	603	68(8.4)	2.20	7.85	2.97	7	8	17.5(0.4)	Norway	SKIERS	Mix
Sandbakk et al.2013	24	HIIT	1000	1000	67.4(7.7)	2.60	7.80	2.95	7	8	17.5(0.4)	Norway	SKIERS	Mix
Sandbakk et al.2013	24	AE	1200	1200	66.6(9.6)	0.20	7.70	2.91	7	8	17.5(0.4)	Norway	SKIERS	Mix
Sheykhlouvand et al.2024	25	SSG	500	500	51.84（2.6）	2.03	2.39	0.76	10	6	26.2(4.0)	Iran	soccer	Male
Sheykhlouvand et al.2024	25	SIT	450	432	51.57（2.6）	2.89	2.65	0.84	10	6	25.8(3.5)	Iran	soccer	Male
Song et	26	SIT	400	400	48.8(2.5)	3.10	2.66	0.84	10	6	19.8（1.3）	Iran	Taekwondo	Male

Study	Study ID	Agent	Model dose (MET-min/week)	Original dose (MET-min/week)	Baseline VO2max	Change (y)	SD	SE	n	Length (weeks)	Age	Country/Region	Sport	Sex
al.2024														
Song et al.2024	26	SSG	350	324	46.4(3.0)	2.10	3.10	0.98	10	6	19.8（1.3）	Iran	Taekwondo	Male
Song et al.2024	26	CG	0	0	46.6(2.9)	0.50	3.06	0.97	10	6	19.8（1.3）	Iran	Taekwondo	Male
Wei et al.2025	27	SIT	200	209	46.93(4.25)	3.97	4.21	1.13	14	8	19.8(2.67)	China	volleyball	Male
Wei et al.2025	27	HIIT	201	216	45.55(5.34)	1.98	5.34	1.43	14	8	20.7(1.4)	China	volleyball	Male
Wells et al.2014	28	CG	0	0	57.1（3.6）	0.50	3.38	1.19	8	8	21.3（2.1）	UK	soccer	Male
Wells et al.2014	28	HIIT	250	228	57.6（5.4）	1.30	5.09	1.80	8	8	21.3（2.1）	UK	soccer	Male
Xu et al.2025	29	RT	200	198	41.9（2.7）	0.20	2.65	0.94	8	8	21.4（2.2）	China	Taekwondo	Male
Xu et al.2025	29	SIT	350	346	42.3（2.8）	2.30	2.95	1.04	8	8	21.7（2.4）	China	Taekwondo	Male
Xu et al.2025	29	CG	0	0	41.8(2.6)	0.10	2.55	0.90	8	8	21.2(2.4)	China	Taekwondo	Male
Yang et al.2024	30	RT	200	200	50.1（2.2）	3.10	2.36	0.75	10	6	21.2(1.4)	China	soccer	Male
Yang et al.2024	30	SIT	500	500	50.2（2.3）	3.10	2.30	0.73	10	6	22.4(1.6)	China	soccer	Male
Yang et al.2024	30	CG	0	0	50.1（2.6）	0.30	2.76	0.87	10	6	22.2(1.9)	China	soccer	Male
Yu et al.2025	31	RT	200	198	64.92（5.18）	3.42	5.43	1.36	16	8	16.81(0.61)	China	endurance runners	Male
Yu et al.2025	31	RT	300	284	65.84（5.95）	3.33	5.76	1.44	16	8	16.75(0.68)	China	endurance runners	Male

Note: MET assignment rules for each modality are provided in Supplementary Table S13. The model dose column reports the MET-min/week value used in the dose-response network meta-analysis. Original dose refers to the extracted or pre-rounded dose value before recoding for model input. CG was assigned a dose of 0 because it represented maintenance of regular sport-specific training without additional supplementary exercise.

14.4 Connectivity

Connectivity is a fundamental assumption in network meta-analysis (NMA). Insufficient connectivity, often resulting from limited direct comparisons, can reduce statistical power and bias results. In our study, connectivity was assessed across all dose and agent levels for each outcome (Figure S14-S15), with no evidence of network disconnection observed.

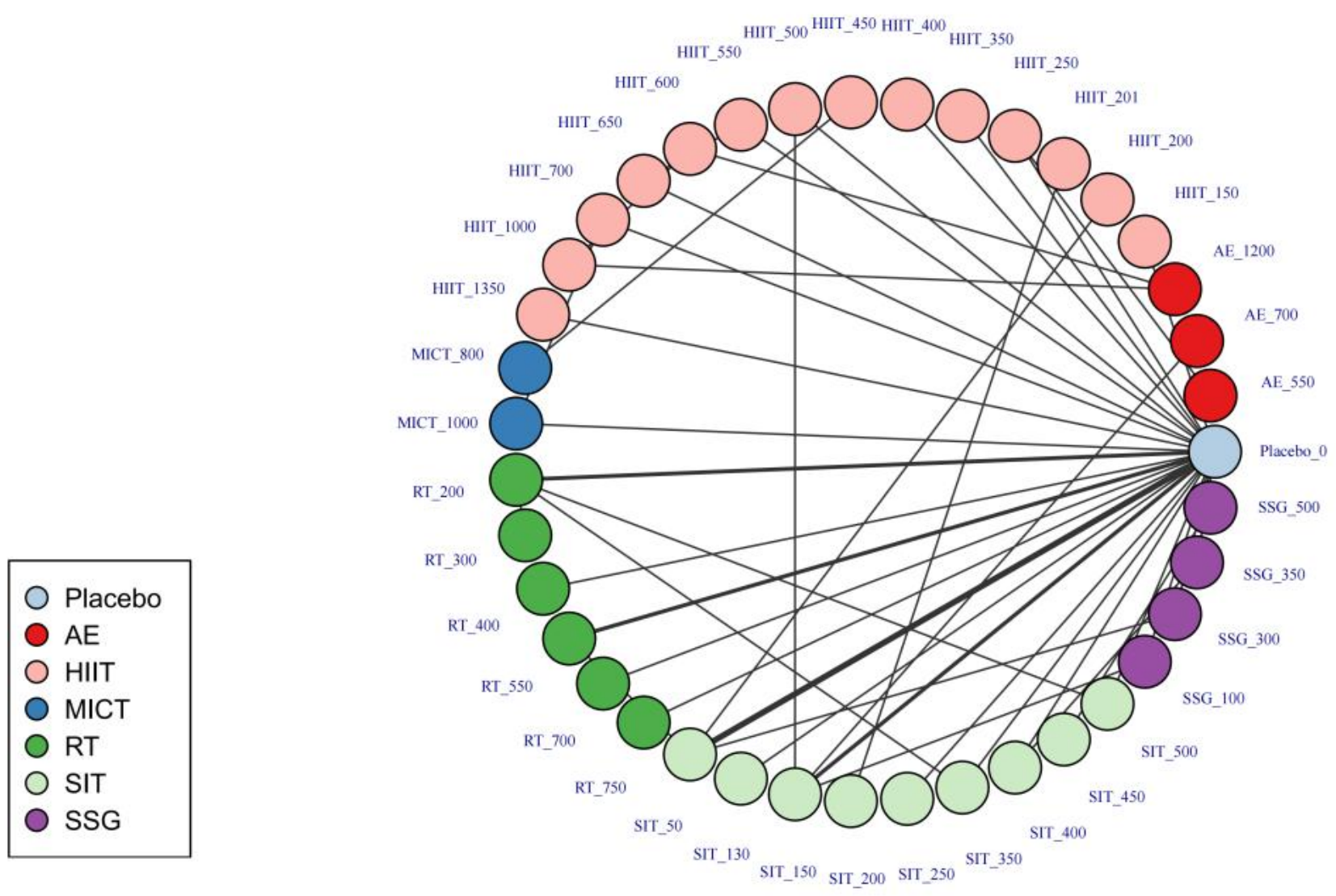

Figure S14. Dose-level network

Line thickness represents the number of studies available for comparison. The first value represents the specific intervention, and the second value corresponds to the dose (METs-min/week).

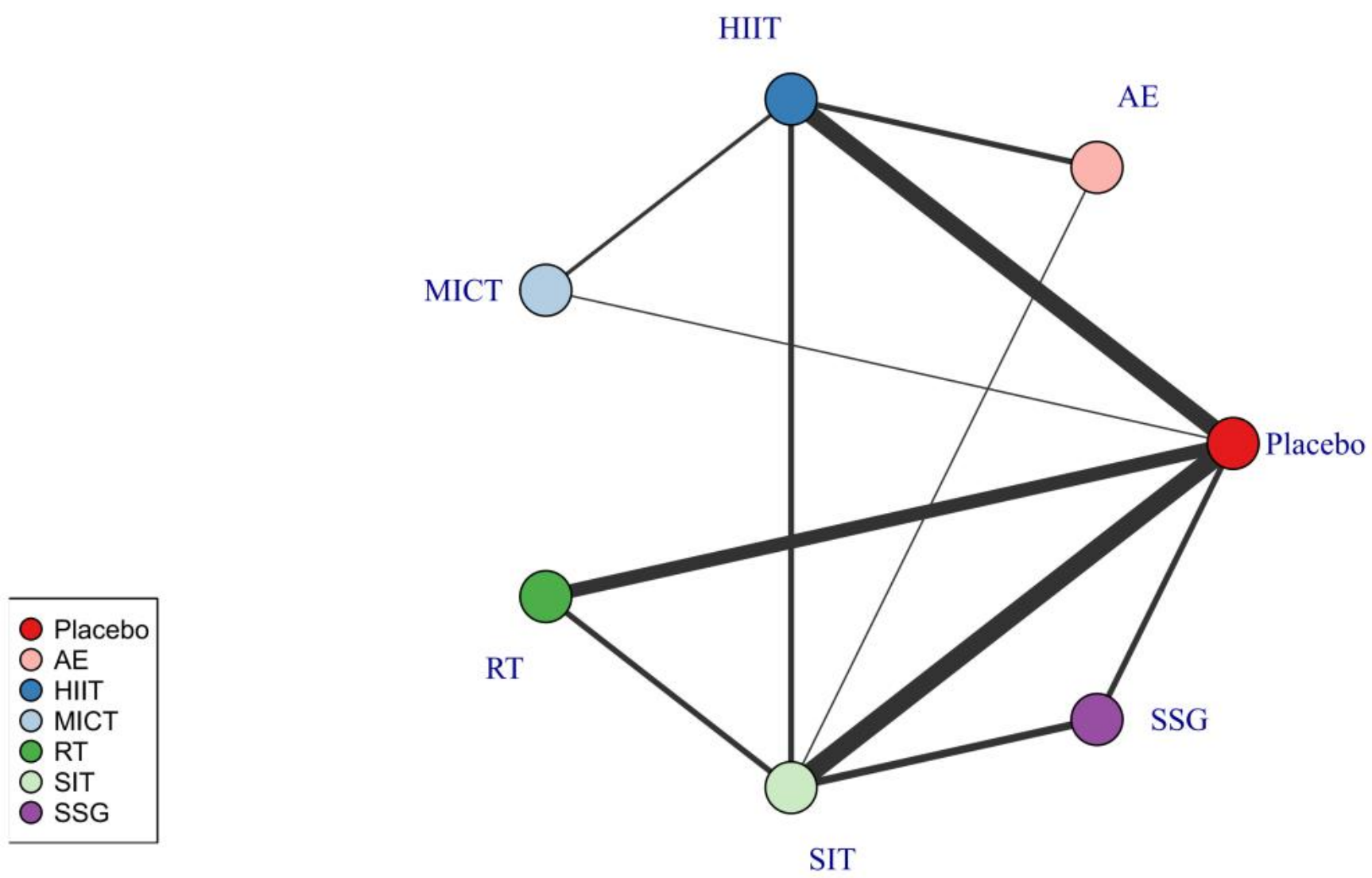

Figure S15. Agent-level network

Line thickness represents the number of studies available for comparison.

14.5 Consistency

We performed a consistency analysis of the data by comparing the consistency of the network i.e., network effect size with the unrelated mean effects UME model i.e., pairwise effect sizes. In practice, we checked whether the bias, the number of estimated parameters in the network, and the Deviance Information Criterion DIC metric were similar for both models, indicating a good fit. The comparison of these parameters showed a good agreement between the different models Table S14.

Table S14. Comparison of consistency and UME model fit.

Model	PD	Deviance	Residual deviance	DIC	SD
Consistent	47.2	67.106	234.838	281.5	1.011
UME	53.2	66.725	234.457	286.6	1.352

Note: UME = unrelated mean effects model; DIC = deviance information criterion; PD = effective number of parameters; SD = standard deviation

14.6 Consistency assessment

Node-splitting was used to compare direct and indirect evidence where both sources were available. This analysis evaluates local inconsistency rather than transitivity. The node-splitting results for the dose-response network meta-analysis are presented in Table S15. The separate descriptive assessment of potential effect modifiers relevant to the transitivity assumption is provided in Supplementary Table S20.

Table S15. Node-splitting analysis of consistency.

==

Comparison	p-value	Median	2.5%	97.5%
SSG_500 vs SIT_450	**0.608**			
-> direct		-0.853	-3.726	1.931
-> indirect		0.465	-1.785	2.876
-> MBNMA		-0.114	-1.804	1.661
SIT_50 vs RT_550	**0.568**			
-> direct		-0.906	-5.369	3.635
-> indirect		-0.950	-2.650	0.663
-> MBNMA		-0.741	-2.309	0.779
RT_300 vs RT_200	**0.086**			
-> direct		-0.114	-4.194	3.938
-> indirect		0.037	-0.061	0.211
-> MBNMA		0.036	-0.058	0.200
MICT_800 vs HIIT_450	**0.562**			
-> direct		-3.996	-9.281	1.457
-> indirect		-0.456	-6.701	6.022
-> MBNMA		-2.662	-6.678	1.759
SIT_200 vs HIIT_201	**0.323**			
-> direct		1.961	-2.113	5.779
-> indirect		-0.230	-1.582	1.099
-> MBNMA		-0.371	-1.647	0.904

SIT_50 vs HIIT_200	**0.576**			
-> direct		-0.488	-2.891	1.901
-> indirect		0.482	-1.041	1.849
-> MBNMA		0.451	-0.887	1.695
HIIT_1000 vs AE_1200	**0.115**			
-> direct		3.103	-3.737	10.632
-> indirect		-4.201	-6.131	-1.996
-> MBNMA		-4.277	-6.159	-2.168
HIIT_600 vs AE_1200	**0.207**			
-> direct		1.816	-6.366	9.431
-> indirect		-4.166	-6.122	-2.115
-> MBNMA		-4.142	-5.990	-2.036
SIT_150 vs AE_700	**0.102**			
-> direct		2.812	-3.264	8.451
-> indirect		-3.903	-6.196	-1.398
-> MBNMA		-3.794	-5.833	-1.513
HIIT_250 vs AE_550	**0.066**			
-> direct		3.800	1.598	5.902
-> indirect		-2.205	-6.411	2.031
-> MBNMA		-3.647	-5.305	-1.730
SSG_350 vs Placebo_0	**0.631**			
-> direct		1.695	-1.293	4.433
-> indirect		2.825	0.687	5.132
-> MBNMA		2.322	0.507	4.299
SSG_300 vs Placebo_0	**0.387**			
-> direct		4.664	-0.404	10.137
-> indirect		2.055	0.261	3.971
-> MBNMA		2.268	0.496	4.175
SSG_100 vs Placebo_0	**0.293**			
-> direct		3.756	0.565	6.981
-> indirect		1.280	-0.008	2.934
-> MBNMA		1.691	0.375	3.529
SIT_500 vs Placebo_0	**0.519**			
-> direct		1.752	-0.634	4.456
-> indirect		2.937	1.548	4.480
-> MBNMA		2.580	1.462	3.848
SIT_400 vs Placebo_0	**0.583**			
-> direct		2.615	-0.341	5.698
-> indirect		2.523	1.323	3.855
-> MBNMA		2.505	1.434	3.706
SIT_350 vs Placebo_0	**0.607**			
-> direct		2.343	-0.577	5.284
-> indirect		2.462	1.269	3.800

-> MBNMA		2.455	1.393	3.610
SIT_250 vs Placebo_0	**0.207**			
-> direct		-0.156	-3.785	3.593
-> indirect		2.523	1.513	3.623
-> MBNMA		2.311	1.319	3.379
SIT_150 vs Placebo_0	**0.519**			
-> direct		2.746	0.579	4.965
-> indirect		1.915	0.963	3.043
-> MBNMA		2.053	1.132	3.031
SIT_130 vs Placebo_0	**0.419**			
-> direct		2.454	-1.175	6.218
-> indirect		1.947	1.025	2.935
-> MBNMA		1.966	1.064	2.959
RT_750 vs Placebo_0	**0.192**			
-> direct		4.510	-5.622	16.387
-> indirect		0.662	-0.820	2.072
-> MBNMA		0.667	-0.688	2.105
RT_700 vs Placebo_0	**0.373**			
-> direct		-0.049	-6.608	6.606
-> indirect		0.685	-0.769	2.261
-> MBNMA		0.664	-0.680	2.086
RT_400 vs Placebo_0	**0.334**			
-> direct		2.493	-0.901	5.985
-> indirect		0.368	-0.934	1.728
-> MBNMA		0.623	-0.640	1.978
RT_200 vs Placebo_0	**0.331**			
-> direct		1.561	-0.364	3.527
-> indirect		0.001	-1.246	1.436
-> MBNMA		0.536	-0.558	1.742
MICT_1000 vs Placebo_0	**0.597**			
-> direct		1.586	-5.317	8.593
-> indirect		-1.784	-7.110	4.054
-> MBNMA		-0.431	-4.646	4.223
HIIT_1350 vs Placebo_0	**0.291**			
-> direct		4.675	1.034	8.419
-> indirect		2.040	0.564	3.683
-> MBNMA		2.475	1.051	4.060
HIIT_700 vs Placebo_0	**0.313**			
-> direct		1.382	-7.353	9.874
-> indirect		2.396	0.984	3.797
-> MBNMA		2.342	0.992	3.725
HIIT_650 vs Placebo_0	**0.623**			
-> direct		1.619	-1.340	4.493

|-> indirect		2.341	0.993	3.872
|-> MBNMA		2.325	0.983	3.678
HIIT_550 vs Placebo_0	**0.362**			
|-> direct		0.576	-3.275	4.388
|-> indirect		2.521	1.086	4.086
|-> MBNMA		2.281	0.955	3.561
HIIT_500 vs Placebo_0	**0.532**			
|-> direct		2.738	-1.076	6.367
|-> indirect		2.173	0.805	3.592
|-> MBNMA		2.248	0.942	3.491
HIIT_400 vs Placebo_0	**0.397**			
|-> direct		3.326	-1.224	7.558
|-> indirect		2.033	0.824	3.341
|-> MBNMA		2.167	0.914	3.358
HIIT_350 vs Placebo_0	**0.389**			
|-> direct		3.130	-1.946	8.512
|-> indirect		2.003	0.842	3.299
|-> MBNMA		2.111	0.901	3.272
HIIT_250 vs Placebo_0	**0.336**			
|-> direct		0.485	-3.829	5.227
|-> indirect		2.019	0.912	3.112
|-> MBNMA		1.952	0.842	3.088
HIIT_150 vs Placebo_0	**0.352**			
|-> direct		0.857	-4.030	5.964
|-> indirect		1.709	0.756	2.869
|-> MBNMA		1.661	0.721	2.803

Note: The first value represents the specific intervention, and the second value corresponds to the dose (METs-min/week).

14.7 Model Selection

For model selection, we adopted a quadratic function model. In accordance with the default settings in MBNMAdose, we applied vague (non-informative) prior distributions to minimize bias and allow the data to drive the results, thereby enabling the model to adapt flexibly to the underlying data structure. This approach is designed to capture a specific form of non-linear dose–response relationship and to identify an optimal dose under the assumption of a quadratic association. Below, we present a comparison of parameter estimates across different models (Table S16). In general, lower DIC indicate a better model fit. As the quadratic function model exhibited the lowest DIC value, we selected it for our analysis.

Table S16. Models fit comparison

Model	DIC	SD	Deviance	Residual deviance	pD
Emax (random treatment effects)	272.9	0.730	229.671	61.939	43.7

Restricted cubic spline (random treatment effects; 3 knots)	284.2	0.616	231.008	63.276	52.9
Log Linear (random treatment effect)	272.6	0.724	229.690	61.958	42.8
Non-parametric monotonically up (random treatment effects)	287.3	0.555	231.767	64.035	54.8
Exponential dose-response function (random treatment effects)	288.3	0.320	238.934	71.202	50.3
Quadratic function (random treatment effects)	272.1	1.048	234.929	67.197	47.1
Spline dose-response functions (random treatment effects)	285.2	1.676	230.956	63.224	54.4
Fractional polynomial dose-response function (random treatment effects)	293.2	0.497	237.338	69.606	55.6

Note: DIC: deviance information criterion; pD: effective number of parameters; SD: standard deviation.

14.8 Dose-Response Curves

Table S17. Dose-response characteristics by training modality.

Agent	Minimum Effective Dose	Characteristic
SIT	Flat curve near zero	Highest efficiency; peak benefit at ~500 METs-min/week
SSG	Flat curve near zero	Moderate dose required for significant benefit
HIIT	Flat curve near zero	Linear positive dose-response to 1,400 METs-min/week
AE	Not significant at any dose	95% CrI crosses zero across entire dose range
MICT	Not significant at any dose	95% CrI crosses zero across entire dose range
RT	Not significant at any dose	Minimal effect on VO2max; targets muscular strength

Note: SIT and HIIT demonstrated the most favorable dose-response profiles. The lack of significant dose-response for AE, MICT and RT likely reflects insufficient stimulus differentiation from athletes' regular training, rather than inefficacy. The doses represent training performed in addition to athletes' regular sport-specific routines.

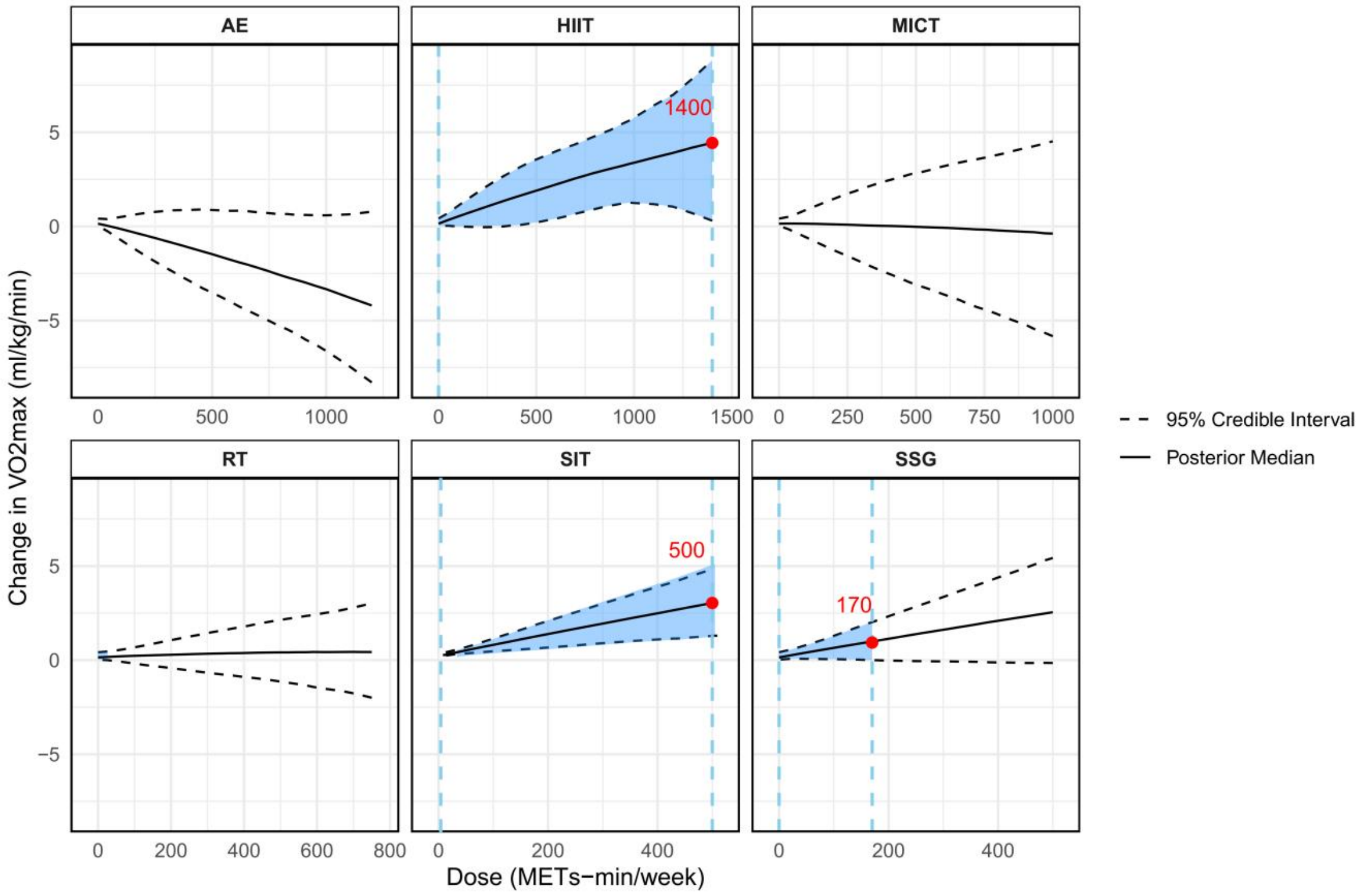

Figure 16. The dose-response relationship between exercise and the increase in athletes' maximal oxygen uptake level.

The blue dashed lines represent the minimum and maximum effective doses, while the blue shaded area indicates the significant region. and the red dots represent the optimal dose points. METs-min/week: Metabolic Equivalents of Task minutes per week

Table S18. Summary of dose-response network meta-analysis result.

Category	Effective dose (METs-min/week)	Optimal dose (METs-min/week)	Mean difference and 95% CI (optimal dose)
AE	NA	NA	NA
MICT	NA	NA	NA
RT	NA	NA	NA
HIIT	0-1400	1400	4.43 mL/kg/min (0.31, 8.83)
SIT	0-500	500	2.99 mL/kg/min (1.10, 4.98)
SSG	0-170	170	1.01 mL/kg/min (0.01, 2.02)

15. Additional Transitivity Assessment

Table S20. Descriptive assessment of potential effect modifiers across treatment nodes.

This table summarizes study-arm characteristics from the main network meta-analysis to support the descriptive assessment of transitivity. Values are summarized at the treatment-node level. Sex distribution is reported by study arm because participant-level sex counts were not consistently available across trials.

Treatment node	No. of studies (arms)	Main sport types represented	Age range, years	Baseline VO2max range, mL/kg/min	Sex distribution, arms	Intervention duration, weeks	Competitive level	Direct CG comparisons	Transitivity concern
AE	18 (18)	rowing, soccer, cycling, running, etc.	17.5-47.2	47.6-70.8	male=8; mixed=1; NR=9	4-12	Not consistently reported	Yes (k=1)	overlap with regular aerobic training.
BFR	3 (3)	basketball, rowing, cycling	19.7-39.6	39.5-63.0	male=2; NR=1	5-12	Not consistently reported	No direct CG comparison	no direct CG comparison; limited sport coverage.
HIIT	30 (32)	basketball, volleyball, rowing, soccer, etc.	13.5-47.2	36.7-81.0	male=18; mixed=4; NR=10	4-16	Not consistently reported	Yes (k=12)	heterogeneity across sports and baseline fitness.
HVT	2 (2)	soccer, endurance athletes	13.5-31.0	55.3-60.5	male=1; NR=1	5-9	Not consistently reported	No direct CG comparison	sparse evidence; no direct CG comparison; limited sport coverage.
IHT	10 (12)	triathlon, swimming, cycling, running	19.1-30.3	56.0-73.5	male=9; NR=3	4-7	Not consistently reported	Yes (k=1)	normoxic/hypoxic contexts pooled.
MICT	5 (6)	endurance athletes, cycling, rowing, running, etc.	15.3-39.2	46.2-80.0	male=2; NR=4	5-10	Not consistently reported	Yes (k=1)	overlap with regular aerobic training.
POL	2 (2)	ultra-endurance running, endurance athletes	31.0-40.6	55.8-60.6	male=1; NR=1	9-12	Not consistently reported	No direct CG comparison	sparse evidence; no direct CG comparison; limited sport coverage.
RT	8 (8)	cycling, volleyball, triathlon, karate, etc.	19.5-29.6	37.9-73.5	male=6; NR=2	6-25	Not consistently reported	Yes (k=7)	heterogeneity across sports and baseline fitness.
SIT	16 (17)	volleyball, running, football, judo, etc.	13.6-29.6	41.9-67.2	male=13; NR=4	4-8	Not consistently reported	Yes (k=10)	heterogeneity across sports and baseline fitness.
SSG	4 (4)	basketball, tennis, soccer	13.8-26.2	45.9-57.4	male=3; NR=1	6-8	Not consistently reported	Yes (k=1)	concentrated in team-ball contexts.
THR	2 (2)	ultra-endurance running, endurance athletes	31.0-36.8	57.1-63.2	male=1; NR=1	9-12	Not consistently reported	No direct CG comparison	sparse evidence; no direct CG comparison; limited sport coverage.
CG	28 (28)	cycling, volleyball,	16.0-47.2	35.2-77.0	male=17;	4-25	Not consistently	Reference node	Active sport-

Treatment node	No. of studies (arms)	Main sport types represented	Age range, years	Baseline VO2max range, mL/kg/min	Sex distribution, arms	Intervention duration, weeks	Competitive level	Direct CG comparisons	Transitivity concern
		triathlon, rowing, etc.			mixed=2; NR=9		reported		specific training; background load varied across sports.

Abbreviations: AE, aerobic exercise; BFR, blood flow restriction training; CG, control group; HIIT, high-intensity interval training; HVT, high-volume training; IHT, intermittent hypoxic training; MICT, moderate-intensity continuous training; POL, polarized training; RT, resistance training; SIT, sprint interval training; SSG, small-sided games; THR, threshold training; VO?max, maximal oxygen uptake. Direct CG comparisons indicate studies in which the treatment node was compared with a control arm maintaining regular sport-specific training.

References

1. Aagaard P, Andersen JL, Bennekou M, et al. Effects of resistance training on endurance capacity and muscle fiber composition in young top-level cyclists. *Scand J Med Sci Sports*. 2011;21(6):1-10. doi:10.1111/j.1600-0838.2010.01283.x

2. Arslan E, Kilit B, Clemente FM, et al. Effects of small-sided games training versus high-intensity interval training approaches in young basketball players. *Int J Environ Res Public Health*. 2022;19(5):2931. doi:10.3390/ijerph19052931

3. Biçer M. The effect of an eight-week strength training program supported with functional sports equipment on Male volleyball players' anaerobic and aerobic power. *Sci Sports*. 2021;36(2):137.e1-137.e9. doi:10.1016/j.scispo.2020.02.006

4. Bilici ÖF, Topateş TK. Comparative effects of high intensity interval and functional training on performance outcomes in adolescent female volleyball players. *BMC Sports Sci Med Rehabil*. 2025;18(1):34-53. doi:10.1186/s13102-025-01476-w

5. Ramos-Campo D, Martínez-Sánchez F, Esteban-García P, Rubio-Arias J, Clemente-Suarez V, Jiménez-Díaz J. The effects of intermittent hypoxia training on hematological and aerobic performance in triathletes. *Acta Physiol Hung*. 2015;102(4):409-418. doi:10.1556/036.102.2015.4.8

6. Ní Chéilleachair NJ, Harrison AJ, Warrington GD. HIIT enhances endurance performance and aerobic characteristics more than high-volume training in trained rowers. *J Sports Sci*. 2017;35(11):1052-1058. doi:10.1080/02640414.2016.1209539

7. Chen L, Zhang Z, Qu W, et al. Effects of blood flow restriction moderate intensity interval training on aerobic and anaerobic capabilities and lower extremity performance in Male college basketball players. *BMC Sports Sci Med Rehabil*. 2025;17(1):44-55. doi:10.1186/s13102-025-01100-x

8. Clark JE. The use of an 8-week mixed-intensity interval endurance-training program improves the aerobic fitness of female soccer players. *J Strength Cond Res*. 2010;24(7):1773-1781. doi:10.1519/JSC.0b013e3181ce2495

9. Czuba M, Wilk R, Karpiński J, Chalimoniuk M, Zajac A, Langfort J. Intermittent hypoxic training improves anaerobic performance in competitive swimmers when implemented into a direct competition mesocycle. Toborek M, ed. *PLOS One*. 2017;12(8):e0180380. doi:10.1371/journal.pone.0180380

10. M C, O FC, K P, A Z, J L. Comparison of the effect of intermittent hypoxic training vs. the live high, train low strategy on aerobic capacity and sports performance in cyclists in normoxia. *Biol Sport*. Published online 2017:1-10. doi:10.5114/biolsport.2018.70750

11. Driller MW, Fell JW, Gregory JR, Shing CM, Williams AD. The effects of high-intensity interval training in well-trained rowers. *Int J Sports Physiol Perform*. 2009;4(1):111-121. doi:10.1123/ijspp.4.1.110

12. Dufour SP, Ponsot E, Zoll J, et al. Exercise training in normobaric hypoxia in endurance runners. I. Improvement in aerobic performance capacity. *J Appl Physiol*. 2006;100(4):1238-1248. doi:10.1152/japplphysiol.00742.2005

13. Fernandez-Fernandez J, Zimek R, Wiewelhove T, Ferrauti A. High-intensity interval training vs. Repeated-sprint training in tennis. *J Strength Cond Res*. 2012;26(1):53-62. doi:10.1519/JSC.0b013e318220b4ff

14. Short-term effects of repeated-sprint training on vertical jump ability and aerobic fitness in collegiate volleyball players during pre-season. *Int J Exerc Sci*. 2022;15(6):1-12. doi:10.70252/UXOP1748

15. Held S, Behringer M, Donath L. Low intensity rowing with blood flow restriction over 5 weeks increases VO2max in elite rowers: a randomized controlled trial. *J Sci Med Sport*. 2020;23(3):304-308. doi:10.1016/j.jsams.2019.10.002

16. Jarstad E, Mamen A. The performance and aerobic endurance effects of high-intensity versus moderate-intensity continuous running. *Appl Physiol Nutr Metab*. 2019;44(9):991-996. doi:10.1139/apnm-2018-0575

17. Javaloyes A, Sarabia JM, Lamberts RP, Moya-Ramon M. Training prescription guided by heart-rate variability in cycling. *Int J Sports Physiol Perform*. 2019;14(1):23-32. doi:10.1123/ijspp.2018-0122

18. Jin K, Cai M, Zhang Y, Wu B, Yang Y. Effects of 6-week sprint interval training compared to traditional training on the running performance of distance runners: a randomized controlled trail. *Front Physiol*. 2025;16:1536287. doi:10.3389/fphys.2025.1536287

19. Jin R, Sun J, Jiang N, Chen C. Influence of 4-week lower extremity high-intensity interval training on energy metabolism and maximal oxygen uptake of elite swimmers. *PeerJ*. 2025;13:e19788. doi:10.7717/peerj.19788

20. Kaynak K, Eryılmaz SK, Aydoğan S, Mihailov D. The effects of 20-m repeated sprint training on aerobic capacity in college volleyball players. *Biomed Hum Kinet*. 2017;9(1):43-50. doi:10.1515/bhk-2017-0007

21. Kelly DT, Cregg CJ, O'Connor PL, Cullen BD, Moyna NM. Physiological and performance responses of sprint interval training and endurance training in gaelic football players. *Eur J Appl Physiol*. 2021;121(8):2265-2275. doi:10.1007/s00421-021-04699-0

22. Kilit B, Arslan E. Effects of high-intensity interval training vs. On-court tennis training in young tennis players. *J Strength Cond Res*. 2019;33(1):188-196. doi:10.1519/JSC.0000000000002766

23. Kim J, Lee N, Trilk J, et al. Effects of sprint interval training on elite judoists. *Int J Sports Med*. 2011;32(12):929-934. doi:10.1055/s-0031-1283183

24. Kirchenberger T, Ketelhut S, Ketelhut RG. Effects of moderate- versus mixed-intensity training on VO2peak in young well-trained rowers. *Sports*. 2021;9(7):92-100. doi:10.3390/sports9070092

25. Kristoffersen M, Sandbakk Ø, Rønnestad BR, Gundersen H. Comparison of short-sprint and heavy strength training on cycling performance. *Front Physiol*. 2019;10:1132. doi:10.3389/fphys.2019.01132

26. Latino F, Susanto N, Anam K, Setyawan H, Saraiello E, Tafuri F. The effects of circuit training versus high-intensity interval training on the endurance of volleyball athletes: a randomized controlled trial. *Retos*. 2024;58(58):1050-1060. doi:10.47197/retos.v58.107877

27. Liu H, Leng B, Li Q, Liu Y, Bao D, Cui Y. The effect of eight-week sprint interval training on aerobic performance of elite badminton players. *Int J Environ Res Public Health*. 2021;18(2):638-648. doi:10.3390/ijerph18020638

28. Liu H, Wang J. The effects of incorporating dry-land short intervals to long aerobic-dominant in-water swimming training on physiological parameters, hormonal factors, and performance: a randomized-controlled intervention study. *J Sports Sci Med*. Published online June 1, 2023:329-337. doi:10.52082/jssm.2023.329

29. Maggioni MA, Bonato M, Stahn A, et al. Effects of ball drills and repeated-sprint-ability training in basketball players. *Int J Sports Physiol Perform*. 2019;14(6):757-764. doi:10.1123/ijspp.2018-0433

30. Mallol M, Bentley DJ, Norton L, Norton K, Mejuto G, Yanci J. Comparison of Reduced-Volume High-Intensity Interval Training and High-Volume Training on Endurance Performance in Triathletes. *Int J*

Sports Physiol Perform. 2019;14(2):239-245. doi:10.1123/ijspp.2018-0359

31. Meckel Y, Gefen Y, Nemet D, Eliakim A. Influence of short vs. Long repetition sprint training on selected fitness components in young soccer players. *J Strength Cond Res*. 2012;26(7):1845-1851. doi:10.1519/JSC.0b013e318236d0f0

32. Millet GP, Jaouen B, Borrani F, Candau R. Effects of concurrent endurance and strength training on running economy and V˙ O2 kinetics. 1-9.

33. Monks L, Seo MW, Kim HB, Jung HC, Song JK. High-intensity interval training and athletic performance in taekwondo athletes. *J Sports Med Phys Fitness*. 2017;57(10):1-9. doi:10.23736/S0022-4707.17.06853-0

34. Morais J, Kilit B, Arslan E, Bragada J, Soylu Y, Marinho D. Effects of on-court tennis training combined with HIIT versus RST on aerobic capacity, speed, agility, jumping ability, and internal loads in young tennis players. *J Hum Kinet*. Published online December 6, 2024:1-13. doi:10.5114/jhk/189691

35. Quezada-Muñoz Y, Rodríguez-Artigas P, Aravena-Sagardia P, et al. Effects of a high-intensity interval training program on body composition and physical fitness in female field hockey players. *Int J Morphol*. 2021;39(5):1323-1330. doi:10.4067/S0717-95022021000501323

36. Ouergui I, Messaoudi H, Chtourou H, et al. Repeated sprint training vs. Repeated high-intensity technique training in adolescent taekwondo athletes—a randomized controlled trial. *Int J Environ Res Public Health*. 2020;17(12):4506. doi:10.3390/ijerph17124506

37. Øvretveit K. Aerobic interval training improves maximal oxygen uptake and reduces body fat in grapplers. *J Sports Med Phys Fitness*. 2020;59(12):1-13. doi:10.23736/S0022-4707.19.09584-7

38. Pérez A, Ramos-Campo DJ, Freitas TT, Rubio-Arias JÁ, Marín-Cascales E, Alcaraz PE. Effect of two different intensity distribution training programmes on aerobic and body composition variables in ultra-endurance runners. *Eur J Sport Sci*. 2019;19(5):636-644. doi:10.1080/17461391.2018.1539124

39. Ponsot E, Dufour SP, Zoll J, et al. Exercise training in normobaric hypoxia in endurance runners. II. Improvement of mitochondrial properties in skeletal muscle. *J Appl Physiol*. 2006;100(4):1249-1257. doi:10.1152/japplphysiol.00361.2005

40. Carrasco-Poyatos M, González-Quílez A, Altini M, Granero-Gallegos A. Heart rate variability-guided training in professional runners: effects on performance and vagal modulation. *Physiol Behav*. 2022;244:113654. doi:10.1016/j.physbeh.2021.113654

41. Pugliese L, Porcelli S, Vezzoli A, et al. Different training modalities improve energy cost and performance in master runners. *Front Physiol*. 2018;9:21-29. doi:10.3389/fphys.2018.00021

42. Robach P, Siebenmann C, Jacobs RA, et al. The role of haemoglobin mass on VO_2 max following normobaric 'live high–train low' in endurance-trained athletes. *Br J Sports Med*. 2012;46(11):822-827. doi:10.1136/bjsports-2012-091078

43. Rodríguez FA, Truijens MJ, Townsend NE, Stray-Gundersen J, Gore CJ, Levine BD. Performance of runners and swimmers after four weeks of intermittent hypobaric hypoxic exposure plus sea level training. *J Appl Physiol*. 2007;103(5):1523-1535. doi:10.1152/japplphysiol.01320.2006

44. Roels B, Millet GP, Marcoux CJL, Coste O, Bentley DJ, Candau RB. Effects of hypoxic interval training on cycling performance: *Med Sci Sports Exercise*. 2005;37(1):138-146. doi:10.1249/01.MSS.0000150077.30672.88

45. Rønnestad BR, Hansen EA, Raastad T. In-season strength maintenance training increases well-trained

cyclists' performance. *Eur J Appl Physiol*. 2010;110(6):1269-1282. doi:10.1007/s00421-010-1622-4

46. Rønnestad BR, Hansen EA, Raastad T. Effect of heavy strength training on thigh muscle cross-sectional area, performance determinants, and performance in well-trained cyclists. *Eur J Appl Physiol*. 2010;108(5):965-975. doi:10.1007/s00421-009-1307-z

47. Sandbakk Ø, Sandbakk SB, Ettema G, Welde B. Effects of intensity and duration in aerobic high-intensity interval training in highly trained junior cross-country skiers. *J Strength Cond Res*. 2013;27(7):1974-1980. doi:10.1519/JSC.0b013e3182752f08

48. Sheykhlouvand M, Gharaat M. Optimal homeostatic stress to maximize the homogeneity of adaptations to interval interventions in soccer players. *Front Physiol*. 2024;15:1377552. doi:10.3389/fphys.2024.1377552

49. Song Y, Sheykhlouvand M. A comparative analysis of high-intensity technique-specific intervals and short sprint interval training in taekwondo athletes: effects on cardiorespiratory fitness and anaerobic power. *J Sports Sci Med*. Published online September 1, 2024:672-683. doi:10.52082/jssm.2024.672

50. Sperlich B, De Marées M, Koehler K, Linville J, Holmberg HC, Mester J. Effects of 5 weeks of high-intensity interval training vs. Volume training in 14-year-old soccer players. *J Strength Cond Res*. 2011;25(5):1271-1278. doi:10.1519/JSC.0b013e3181d67c38

51. Stöggl T, Sperlich B. Polarized training has greater impact on key endurance variables than threshold, high intensity, or high volume training. *Front Physiol*. 2014;5:1-9. doi:10.3389/fphys.2014.00033

52. Talsnes RK, Van Den Tillaar R, Sandbakk Ø. Effects of increased load of low-versus high-intensity endurance training on performance and physiological adaptations in endurance athletes. *International journal of sports physiology and performance*. 2021;17(2):216-225. doi:10.1123/ijspp.2021-0190

53. Tangchaisuriya P, Chuensiri N, Tanaka H, Suksom D. Physiological adaptations to high-intensity interval training combined with blood flow restriction in masters road cyclists. *Med Sci Sports Exercise*. 2022;54(5):831-840. doi:10.1249/MSS.0000000000002857

54. Ventura N, Hoppeler H, Seiler R, Binggeli A, Mullis P, Vogt M. The response of trained athletes to six weeks of endurance training in hypoxia or normoxia. *Int J Sports Med*. 2003;24(3):166-172. doi:10.1055/s-2003-39086

55. Wang L, Wang J. Effects of high-intensity training on endurance in basketball players. *Rev Bras Med Esporte*. 2023;29:e2022_0633. doi:10.1590/1517-8692202329012022_0633

56. Wei C, An J, Zhou L. The effects of 8 weeks of sprint interval training on repeated sprinting and specialized ability in college volleyball players. Akaras E, ed. *PLOS One*. 2025;20(7):e0327561. doi:10.1371/journal.pone.0327561

57. Wells C, Edwards A, Fysh M, Drust B. Effects of high-intensity running training on soccer-specific fitness in professional male players. *Appl Physiol Nutr Metab*. 2014;39(7):763-769. doi:10.1139/apnm-2013-0199

58. Xu Y, Wang S. Sequencing effects of concurrent resistance and short sprint interval training on physical fitness, and aerobic and anaerobic performance of karate athletes. *J Sports Sci Med*. Published online March 1, 2025:205-216. doi:10.52082/jssm.2025.205

59. Yang G, Chen W, Qi D, Zhang J, Men Z. The effects of a 6-week plyometric and sprint interval training intervention on soccer player's physical performance. *J Sports Sci Med*. Published online September 1, 2024:526-536. doi:10.52082/jssm.2024.526

60. Zoll J, Ponsot E, Dufour S, et al. Exercise training in normobaric hypoxia in endurance runners. III.

Muscular adjustments of selected gene transcripts. *J Appl Physiol*. 2006;100(4):1258-1266. doi:10.1152/japplphysiol.00359.2005

61. Mallol et al.2019.

62. Yu S, Zhou S, Peng D, Jie D. Effect of complex training on lower limb strength and running economy in adolescent distance runners. *Front Physiol*. 2025;16:1718150. doi:10.3389/fphys.2025.1718150

63. Ainsworth BE, Haskell WL, Whitt MC, et al. Compendium of physical activities: an update of activity codes and MET intensities. *Med Sci Sports Exerc*. 2000;32(9 Suppl):S498-504. doi:10.1097/00005768-200009001-00009

64. Gibala MJ, Little JP, Macdonald MJ, Hawley JA. Physiological adaptations to low-volume, high-intensity interval training in health and disease. *J Physiol*. 2012;590(5):1077-1084. doi:10.1113/jphysiol.2011.224725

65. Buchheit M, Laursen PB. High-intensity interval training, solutions to the programming puzzle: Part I: cardiopulmonary emphasis. *Sports Med*. 2013;43(5):313-338. doi:10.1007/s40279-013-0029-x

66. Seiler S. What is best practice for training intensity and duration distribution in endurance athletes? *Int J Sports Physiol Perform*. 2010;5(3):276-291. doi:10.1123/ijspp.5.3.276

67. Garber CE, Blissmer B, Deschenes MR, et al. Quantity and Quality of Exercise for Developing and Maintaining Cardiorespiratory, Musculoskeletal, and Neuromotor Fitness in Apparently Healthy Adults: Guidance for Prescribing Exercise. *Med Sci Sports Exerc*. 2011;43(7):1334-1359. doi:10.1249/MSS.0b013e318213fefb

68. Hill-Haas SV, Dawson B, Impellizzeri FM, Coutts AJ. Physiology of Small-Sided Games Training in Football: A Systematic Review. *Sports Medicine*. 2011;41(3):199-220. doi:10.2165/11539740-000000000-00000

69. Progression Models in Resistance Training for Healthy Adults. *Medicine & Science in Sports & Exercise*. 2009;41(3):687-708. doi:10.1249/MSS.0b013e3181915670
