# Supplementary material for: Comparative and dose-response effects of supplementary exercise training on VO2max in trained athletes: a Systematic Review and Bayesian network meta-analysis
Source: Front Physiol. 2026 Jul 30;17:1906583. doi: 10.3389/fphys.2026.1906583 (PMC13467872; doi:10.3389/fphys.2026.1906583)
Supplement: Supplementary file 2 [file Table1.docx]

# **Supplementary Appendix**

**1. Search Strategy**

The following search strategies were executed on April 3, 2026. No language or date restrictions were applied.

**1.1 PubMed (n = 1,171)**

("Athletes"[Mesh] OR athlete*[Title/Abstract] OR "sports personnel"[Title/Abstract] OR

sportsmen[Title/Abstract] OR sportswomen[Title/Abstract] OR "elite athletes"[Title/Abstract])

AND

("Exercise"[Mesh] OR "Exercise Therapy"[Mesh] OR "Motor Activity"[Mesh] OR

"Sports"[Mesh] OR "Resistance Training"[Mesh] OR "Yoga"[Mesh] OR "Tai Ji"[Mesh] OR

"Walking"[Mesh] OR "Physical Exertion"[Mesh] OR "physical activity"[Title/Abstract] OR

movement[Title/Abstract] OR fitness[Title/Abstract] OR "aerobic exercise"[Title/Abstract] OR

"high-intensity interval training"[Title/Abstract] OR "HIIT"[Title/Abstract] OR

"resistance training"[Title/Abstract] OR "strength training"[Title/Abstract] OR

"endurance exercise"[Title/Abstract] OR yoga[Title/Abstract] OR "Tai Chi"[Title/Abstract] OR

pilates[Title/Abstract] OR dance[Title/Abstract] OR walking[Title/Abstract] OR

jogging[Title/Abstract] OR stretching[Title/Abstract] OR "mind-body exercise"[Title/Abstract])

AND

("Oxygen Consumption"[Mesh] OR "Cardiorespiratory Fitness"[Mesh] OR

"maximal oxygen uptake"[Title/Abstract] OR "maximal oxygen consumption"[Title/Abstract] OR

VO2max[Title/Abstract] OR "VO2 max"[Title/Abstract] OR "maximal aerobic capacity"[Title/Abstract] OR

"cardiorespiratory fitness"[Title/Abstract] OR "aerobic capacity"[Title/Abstract])

AND

(randomized controlled trial[pt] OR "Randomized Controlled Trial"[Title/Abstract] OR

RCT[Title/Abstract] OR "randomized trial"[Title/Abstract] OR "controlled clinical trial"[Title/Abstract] OR

"randomized study"[Title/Abstract] OR "randomized clinical trial"[Title/Abstract] OR

RCTs[Title/Abstract] OR "randomized clinical studies"[Title/Abstract] OR

"intervention trial"[Title/Abstract] OR "randomized intervention"[Title/Abstract] OR

trial[Title/Abstract] OR "experimental study"[Title/Abstract])

**1.2 Web of Science (n = 723)**

TS=(athlete* OR "sports personnel" OR sportsmen OR sportswomen OR "elite athletes")

AND

TS=(exercise* OR "physical activity" OR movement OR fitness OR "aerobic exercise" OR

"high-intensity interval training" OR HIIT OR "resistance training" OR "strength training" OR

"endurance exercise" OR yoga OR "Tai Chi" OR "Tai Ji" OR pilates OR dance OR walking OR

jogging OR stretching OR "mind-body exercise")

AND

TS=("maximal oxygen uptake" OR "maximal oxygen consumption" OR VO2max OR "VO2 max" OR

"maximal aerobic capacity" OR "cardiorespiratory fitness" OR "aerobic capacity" OR

"oxygen consumption")

AND

TS=("randomized controlled trial" OR RCT OR "randomized trial" OR "controlled clinical trial" OR

"randomized study" OR "randomized clinical trial" OR RCTs OR "randomized clinical studies" OR

"intervention trial" OR "randomized intervention" OR trial OR "experimental study")

**1.3 Embase (n = 1,749)**

('athlete'/exp OR athlete*:ti,ab OR 'sports personnel':ti,ab OR sportsmen:ti,ab OR

sportswomen:ti,ab OR 'elite athletes':ti,ab)

AND

('exercise'/exp OR 'motor activity'/exp OR 'sport'/exp OR 'resistance training'/exp OR

'yoga'/exp OR 'tai chi'/exp OR 'walking'/exp OR 'physical activity':ti,ab OR

movement:ti,ab OR fitness:ti,ab OR 'aerobic exercise':ti,ab OR

'high-intensity interval training':ti,ab OR hiit:ti,ab OR 'resistance training':ti,ab OR

'strength training':ti,ab OR 'endurance exercise':ti,ab OR yoga:ti,ab OR 'tai chi':ti,ab OR

pilates:ti,ab OR dance:ti,ab OR walking:ti,ab OR jogging:ti,ab OR stretching:ti,ab OR

'mind-body exercise':ti,ab)

AND

('oxygen consumption'/exp OR 'cardiorespiratory fitness'/exp OR

'maximal oxygen uptake':ti,ab OR 'maximal oxygen consumption':ti,ab OR vo2max:ti,ab OR

'vo2 max':ti,ab OR 'maximal aerobic capacity':ti,ab OR 'cardiorespiratory fitness':ti,ab OR

'aerobic capacity':ti,ab)

AND

('randomized controlled trial'/exp OR 'controlled clinical trial'/exp OR

'randomized controlled trial':ti,ab OR rct:ti,ab OR 'randomized trial':ti,ab OR

'controlled clinical trial':ti,ab OR 'randomized study':ti,ab OR 'randomized clinical trial':ti,ab OR

rcts:ti,ab OR 'randomized clinical studies':ti,ab OR 'intervention trial':ti,ab OR

'randomized intervention':ti,ab OR trial:ti,ab OR 'experimental study':ti,ab)

**1.4 Cochrane Library (n = 667)**

([mh "Athletes"] OR athlete*:ti,ab,kw OR "sports personnel":ti,ab,kw OR

sportsmen:ti,ab,kw OR sportswomen:ti,ab,kw OR "elite athletes":ti,ab,kw)

AND

([mh "Exercise"] OR [mh "Exercise Therapy"] OR [mh "Motor Activity"] OR [mh "Sports"] OR

[mh "Resistance Training"] OR [mh "Yoga"] OR [mh "Tai Ji"] OR [mh "Walking"] OR

[mh "Physical Exertion"] OR "physical activity":ti,ab,kw OR movement:ti,ab,kw OR

fitness:ti,ab,kw OR "aerobic exercise":ti,ab,kw OR "high-intensity interval training":ti,ab,kw OR

HIIT:ti,ab,kw OR "resistance training":ti,ab,kw OR "strength training":ti,ab,kw OR

"endurance exercise":ti,ab,kw OR yoga:ti,ab,kw OR "Tai Chi":ti,ab,kw OR pilates:ti,ab,kw OR

dance:ti,ab,kw OR walking:ti,ab,kw OR jogging:ti,ab,kw OR stretching:ti,ab,kw OR

"mind-body exercise":ti,ab,kw)

AND

([mh "Oxygen Consumption"] OR [mh "Cardiorespiratory Fitness"] OR

"maximal oxygen uptake":ti,ab,kw OR "maximal oxygen consumption":ti,ab,kw OR

VO2max:ti,ab,kw OR "VO2 max":ti,ab,kw OR "maximal aerobic capacity":ti,ab,kw OR

"cardiorespiratory fitness":ti,ab,kw OR "aerobic capacity":ti,ab,kw)

AND

("randomized controlled trial":ti,ab,kw OR RCT:ti,ab,kw OR "randomized trial":ti,ab,kw OR

"controlled clinical trial":ti,ab,kw OR "randomized study":ti,ab,kw OR

"randomized clinical trial":ti,ab,kw OR RCTs:ti,ab,kw OR

"randomized clinical studies":ti,ab,kw OR "intervention trial":ti,ab,kw OR

"randomized intervention":ti,ab,kw OR trial:ti,ab,kw OR "experimental study":ti,ab,kw)

**2.Definitions of exercise modalities**

**Table S1.** Definitions of exercise modalities

| Abbreviation | Full name | Definitions |
| --- | --- | --- |
| AE | Aerobic Exercise | Aerobic exercise is performed by repeating sequences of light-to-moderate intensity activities for extended periods of time. e.g., walking, bicycle, and treadmill training etc. |
| BFR | Blood Flow Restriction | Blood flow restriction (BFR) training is a method where athletes exercise under controlled blood flow occlusion using pneumatic tourniquets applied to the proximal limbs to induce intense local hypoxia and metabolic accumulation, thereby stimulating cardiovascular adaptations and improving overall athletic performance. |
| CG | Control group | Maintenance of regular sport-specific training without additional structured supplementary exercise intervention |
| IHT | Intermittent Hypoxic Training | Intermittent hypoxic training (IHT) is a method where athletes rest or train under intermittent bouts of hypobaric or normobaric hypoxia to simulate altitude acclimatization and improve sea-level athletic performance. |
| SSG | Small-Sided Games | Small-sided games (SSG) are modified, scaled-down versions of team sports played on a reduced pitch with fewer players, designed to replicate the physical and technical demands of competition while targeting specific physiological adaptations. |
| RT | Resistance Training | Exercise training designed to improve the strength, power, endurance and size of skeletal muscles. |
| SIT | Sprint Interval Training | Sprint interval training (SIT) is a form of high-intensity intermittent exercise that involves repeated bouts of very short (≤30 s), all-out maximal effort sprints interspersed with extended periods of active or passive recovery (2–4 min), aimed at enhancing both anaerobic and aerobic energy systems. |
| HIIT | High-Intensity Interval Training | It typically consists of alternating high-intensity exercise lasting 15 seconds to 4 minutes with passive or low-intensity recovery periods. The heart rate during the high-intensity phase approaches 80% to 95% of the maximum heart rate. |
| POL | Polarized Training | Polarized Training (POL) is a periodization model that concentrates training intensity distribution predominantly at low intensities (zone 1, < first ventilatory threshold) and high intensities (zone 3, > second ventilatory threshold) with very little work in the moderate-intensity zone (zone 2), typically following a 75–80% / 5–10% / 15–20% distribution, aiming to maximize endurance performance adaptations. |
| HVT | High-Volume Training | High-volume training (HVT) is a continuous endurance training method performed at low to moderate intensities (typically 60–75% of maximal heart rate or below the first ventilatory threshold) for prolonged durations per session, aimed at enhancing aerobic capacity through high accumulative training volume. |
| MICT | Moderate-Intensity Continuous Training | Moderate-intensity continuous training (MICT) is a form of aerobic exercise performed at a steady, submaximal intensity (typically 70–80% of maximal heart rate or between the first and second ventilatory thresholds) for a sustained duration without rest intervals, aiming to improve cardiorespiratory fitness and metabolic function. |
| THR | Threshold Training | Threshold training (THR) is a training method where athletes perform a high volume of exercise at an intensity just below, at, or slightly above the lactate threshold (LT) to improve the body's ability to sustain a high work rate before fatigue from lactate accumulation occurs. |

**3. Study Characteristics**

**Table S2.** Characteristics of included studies — Network Meta-Analysis.

| Studyname | Interventions (n) | Sports items | Gender | baseline | Length(weeks) | Age  (mean±sd) | Region |
| --- | --- | --- | --- | --- | --- | --- | --- |
|  |  |  |  |  |  |  |  |
| aagaard et al.2011[1] | RT (7) | cyclists | Male | 73.5 (8.2) | 16 | 19.5(0.8) | Denmark |
|  | CG (7) |  | Male | 71.5 (6.0) | 16 | 19.5(0.8) |  |
| Arslan et al.2022[2] | HIIT (6) | basketball | Male | 45.8 (0.85) | 6 | 14.5(0.5) | Turkey |
|  | SSG (6) |  | Male | 46.29 (1.03) | 6 | 14.5(0.5) |  |
| Bicer et al.2020[3] | RT (13) | volleyball | Male | 37.85 (4.34) | 8 | 27.08(3.99) | Turkey |
|  | CG (13) |  | Male | 51.81(19.03) | 8 | 27.46(4.05) |  |
| Bilici et al.2026[4] | HIIT (11) | volleyball | Female | 36.73 (2.37) | 12 | 16.18(1.17) | Turkey |
|  | HIIT (10) |  | Female | 37.40 (4.86) | 12 | 16.00(1.05) |  |
|  | CG (11) |  | Female | 37.82 (3.49) | 12 | 16.64(1.29) |  |
| Campo et al.2015[5] | IHT (9) | Triathlon | Male | 59.5 (5.0) | 7 | 26.0(6.7) | Spain |
|  | CG (9) |  | Male | 58.9 (4.5) | 7 | 29.3(6.8) |  |
| Chéilleachair et al.2017[6] | AE (9) | rowers | Mix | 61.7(6.6) | 8 | 22(4) | Ireland |
|  | HIIT (10) |  | Mix | 60.6(5.3) | 8 | 22(4) |  |
| Chen et al.2025[7] | BFR (17) | basketball | Male | 39.48(6.22) | 6 | 19.70(1.31) | China |
|  | HIIT (16) |  | Male | 39.75(6.92) | 6 | 19.87(1.25) |  |
| Clark et al.2010[8] | HIIT (17) | soccer | Female | 49.69(1.15) | 8 | 18.7(0.37) | USA |
|  | AE (15) |  | Female | 49.54(1.89) | 8 | 18.6(0.51) |  |
| Czuba et al.2017[9] | HIIT (7) | swimmers | Male | 54.0(6.2) | 4 | 20.5(1.3) | Poland |
|  | IHT (8) |  | Male | 56.0(4.0) | 4 | 19.1(1.3) |  |
| Czuba et al.2018[10] | AE (10) | cyclists | Male | 67.0(2.9) | 4 | 21.8(4.0) | Poland |
|  | IHT (10) |  | Male | 66.0(4.0) | 4 | 20.5(2.9) |  |
|  | IHT (10) |  | Male | 67.6(2.7) | 4 | 20.7(3.1) |  |
| driller et al.2009[11] | HIIT (10) | rowers | Mix | 53.2(6.4) | 4 | 19(2) | Australia |
|  | CG (10) |  | Mix | 54.8(7.3) | 4 | 19(2) |  |
| dufour et al.2006[12] | AE (9) | endurance runners | Male | 61.5(1.1) | 6 | 30.3(6.1) | France |
|  | IHT (9) |  | Male | 64.2(1.2) | 6 | 30.3(6.3) |  |
| Fernandez et al.2012[13] | HIIT (11) | tennis | Male | 56.3(4.0) | 6 | 22.6(4.8) | Germany |
|  | CG (9) |  | Male | 57.3(4.0) | 6 | 22.1(3.3) |  |
| Gantois et al.2022[14] | SIT (10) | volleyball | Male | 50.39(4.30) | 6 | 21.6(3.34) | Brazil |
|  | CG (9) |  | Male | 48.84(5.89) | 6 | 21.33(2.91) |  |
| held et al.2020[15] | AE (15) | rowers | Mix | 63.2(8.5) | 5 | 21.7(3.7) | Germany |
|  | BFR (16) |  | Mix | 63.0(7.0) | 5 | 21.9(3.2) |  |
| Jarstad et al.2019[16] | MICT (7) | endurance athletes | Mix | 80(4) | 10 | 28(5) | Norway |
|  | HIIT (7) |  | Mix | 81(3) | 10 | 28(5) |  |
|  | CG (6) |  | Mix | 77(4) | 10 | 28(5) |  |
| javaloyes et al.2019[17] | MICT (9) | cyclists | Male | 56.34(7.58) | 8 | 39.22(5.33) | spain |
|  | AE (8) |  | Male | 54.30(7.81) | 8 | 37.62(7.09) |  |
| Jin et al.2025.January[18] | HIIT (12) | swimmers | Mix | 51.48(3.85) | 4 | 19.94(1.4) | China |
|  | CG (12) |  | Mix | 49.40(3.75) | 4 | 21.17(1.6) |  |
| Jin et al.2025.March[19] | AE (10) | distance runners | Male | 68.8(7.0) | 6 | 20(1) | China |
|  | SIT (10) |  | Male | 67.2(5.0) | 6 | 19(1) |  |
| kaynak et al.2017[20] | SIT (9) | volleyball | Male | 50(3.6) | 6 | 21.2(1.3) | volleyball |
|  | CG (9) |  | Male | 50.4(4) | 6 | 21.2(1.6) |  |
| kelly et al.2021[21] | SIT (13) | football | Male | 52(6) | 6 | 26.5(4.9) | Turkey |
|  | CG (12) |  | Male | 53.4(4.7) | 6 | 25.4(2.6) |  |
| Kilit et al.2018[22] | HIIT (14) | tennis | Male | 45.4(1.9) | 6 | 13.8(0.4) | Turkey |
|  | SSG (15) |  | Male | 45.9(1.7) | 6 | 13.8(0.4) |  |
| Kim et al.2011[23] | SIT (11) | Judo | Male | 49.84(4.08) | 8 | 20.00(1.10) | Korea |
|  | CG (19) |  | Male | 46.63(4.99) | 8 | 19.94(1.16) |  |
| Kirchenberger et al.2021[24] | HIIT (10) | Rowers | Male | 59.4(2.3) | 8 | 15.4(1.3) | Germany |
|  | MICT (7) |  | Male | 58.4(5.7) | 8 | 15.3(1.2) |  |
| Kristoffersen et al.2019[25] | RT (16) | cyclists | Mix | 61.1(6.0) | 6 | 29.6(0.6) | Norway |
|  | SIT (16) |  | Mix | 63.3(5.9) | 6 | 29.6(0.6) |  |
| Latino et al.2024[26] | HIIT (15) | volleyball | Male | 48.45(1.88) | 16 | 19.14(1.61) | Italy |
|  | CG (15) |  | Male | 47.78(2.42) | 16 | 19.14(1.61) |  |
| Liu et al.2021[27] | SIT (8) | Badminton | Male | 56.8(7.0) | 8 | 20.0(1.3) | China |
|  | CG (8) |  | Male | 55.8(8.0) | 8 | 21.5(2.2) |  |
| Liu et al.2023[28] | SIT (8) | swimmers | Male | 54.6(3.6) | 4 | 25(6) | China |
|  | CG (8) |  | Male | 54.5(3.5) | 4 | 25(6) |  |
| Maggioni et al.2019[29] | SSG (12) | Basketball | NR | 57.4(6.7) | 8 | 19(1) | Italy |
|  | SIT (9) |  | NR | 55.7(7.2) | 8 | 19(1) |  |
|  | CG (9) |  | NR | 56.7(2.8) | 8 | 19(1) |  |
| Mallol et al.2019[30] | HIIT (8) | triathletes | Mix | 42.4(5.2) | 4 | 42.9(12.1) | Australia |
|  | CG (8) |  | Mix | 43.1(4.5) | 4 | 37.2(13.3) |  |
| meckel et al.2012[31] | HIIT (13) | soccer | Male | 44.9(5.2) | 7 | 14.5(0.6) | Israel |
|  | SIT (11) |  | Male | 44.6(5.3) | 7 | 14.5(0.6) |  |
| Millet et al.2002[32] | RT (7) | triathletes | NR | 69.7(3.60 | 14 | 24.3(5.2) | France |
|  | CG (8) |  | NR | 67.6(6.4) | 14 | 21.4(2.1) |  |
| MonKS et al.2017[33] | HIIT (16) | Taekwondo | Mix | 56.1(1.38) | 4 | 18-22 | Korea |
|  | AE (17) |  | Mix | 51.5(1.34) | 4 | 18-22 |  |
| Morais et al.2025[34] | HIIT (12) | tennis | Male | 45.21(2.06) | 6 | 13.6(0.3) | Spain |
|  | SIT (12) |  | Male | 45.50(1.85) | 6 | 13.6(0.3) |  |
| Muñoz et al.2021[35] | HIIT (10) | Field Hockey | Female | 37.50(6.04) | 8 | 23.6(2.8) | Chile |
|  | CG (10) |  | Female | 35.25(5.50) | 8 | 23.6(3.0) |  |
| ouergui et al.2020[36] | SIT (13) | Taekwondo | Mix | 51.1(4.9) | 4 | 16(1) | Tunisia |
|  | SIT (13) |  | Mix | 50.9(3.4) | 4 | 16(1) |  |
|  | CG (13) |  | Mix | 48.3(3.2) | 4 | 16(1) |  |
| ØVRETVEIT et al.2019[37] | HIIT (6) | BJJ | Male | 52.7(5.3) | 6 | 30.3(4.0) | Norway |
|  | CG (6) |  | Male | 53.3(1.2) | 6 | 30.3(4.0) |  |
| Pérez et al.2019[38] | POL (11) | ultra-endurance runners | Male | 55.8(4.9) | 12 | 40.6(9.7) | Spain |
|  | THR (9) |  | Male | 57.1(5.2) | 12 | 36.8(9.2) |  |
| ponsot et al.2006[39] | HIIT (7) | endurance runners | Male | 62.0(1.4) | 6 | 31.3(2.3) | France |
|  | IHT (8) |  | Male | 63.6(1.1) | 6 | 29.9(2.3) |  |
| Poyatos et al.2022[40] | MICT (6) | runners | NR | 58.8(4.99) | 8 | 31.5(8.36) | Spain |
|  | AE (6) |  | NR | 61(7.37) | 8 | 31.2(10.3) |  |
| Pugliese et al.2018[41] | AE (10) | Runners | Male | 47.6(4.2) | 8 | 47.2(7.4) | Italy |
|  | HIIT (10) |  | Male | 48.8(5.5) | 8 | 47.2(7.4) |  |
|  | CG (10) |  | Male | 48.4(4.4) | 8 | 47.2(7.4) |  |
| robach et al.2012[42] | AE (6) | endurance runners | Mix | 70.8(5.3) | 4 | 29(6) | France |
|  | IHT (10) |  | Mix | 73.5(2.9) | 4 | 29(6) |  |
| Rodriguez et al.2007[43] | AE (12) | endurance runners | Mix | 55.8(8.0) | 4 | 22.8(9.3) | USA |
|  | IHT (11) |  | Mix | 56.7(8.9) | 4 | 22.2(7.0) |  |
| Roels et al.2005[44] | AE (8) | endurance runners | Male | 62.43(5.31) | 7 | 33.0(2.85) | France |
|  | IHT (10) |  | Male | 70.43(5.55) | 7 | 22.8(2.5) |  |
|  | IHT (11) |  | Male | 57.08(7.30) | 7 | 21.9(2.8) |  |
| Ronnestad et al.2010.August[45] | RT (6) | cyclists | Male | 65.2(2.2) | 25 | 29(3) | Norway |
|  | CG (6) |  | Mix | 67.3(2.7) | 25 | 31(3) |  |
| Rønnestad et al.2010.November[46] | RT (11) | cyclists | Male | 66.8(1.6) | 12 | 27(2) | Norway |
|  | CG (9) |  | Mix | 65.9(2.0) | 12 | 30(2) |  |
| Sandbakk et al.2013[47] | HIIT (7) | SKIERS | Mix | 67.4(7.7) | 8 | 17.5(0.4) | Norway |
|  | AE (7) |  | Mix | 67.4(7.7) | 8 | 17.5(0.4) |  |
| Sheykhlouvand et al.2024[48] | SIT (10) | soccer | Male | 51.57(2.6) | 6 | 25.8(3.5) | Iran |
|  | SSG (10) |  | Male | 51.84(2.6) | 6 | 26.2(4.0) |  |
| Song et al.2024[49] | HIIT (10) | Taekwondo | Male | 46.4(3.0) | 6 | 19.8(1.3) | Iran |
|  | HIIT (10) |  | Male | 48.8(2.5) | 6 | 19.8(1.3) |  |
|  | CG (10) |  | Male | 46.6(2.9) | 6 | 19.8(1.3) |  |
| Sperlich et al.2011[50] | HIIT (8) | soccer | Male | 55.1(4.9) | 5 | 13.5(0.4) | Germany |
|  | HVT (8) |  | Male | 55.3(4.3) | 5 | 13.5(0.4) |  |
| Stöggl et al.2014[51] | HIIT (10) | endurance athletes | Mix | 63.7(7.1) | 9 | 31(6) | Germany |
|  | HVT (11) |  | Mix | 60.5(9.4) | 9 | 31(6) |  |
|  | POL (11) |  | Mix | 60.6(8.3) | 9 | 31(6) |  |
|  | THR (8) |  | Mix | 63.2(4.6) | 9 | 31(6) |  |
| Talsnes et al.2022[52] | AE (22) | Cross-Country Skiing、Biathlon | Mix | 65.7(7.6) | 8 | 18(1) | Norway |
|  | HIIT (20) |  | Mix | 66.7(7.1) | 8 | 18(1) |  |
| Tangchaisuriya et al.2022[53] | AE (16) | cyclists | Male | 55.9(5.7) | 12 | 41.5(4.5) | Thailand |
|  | BFR (17) |  | Male | 54.8(4.7) | 12 | 39.6(3.8) |  |
|  | HIIT (17) |  | Male | 55.2(6.7) | 12 | 41.7(3.8) |  |
| ventura et al.2003[54] | AE (7) | cyclists | Mix | 62.3(1.5) | 6 | 25(2.2) | Switzerland |
|  | IHT (7) |  | Mix | 62.1(2.7) | 6 | 25(2.2) |  |
| Wang et al.2022[55] | HIIT (5) | Basketball | NR | 45.2(3.6) | 5 | 18-20 | China |
|  | MICT (5) |  | NR | 46.2(3.7) | 5 | 18-20 |  |
|  | MICT (5) |  | NR | 46.5(3.7) | 5 | 18-20 |  |
| Wei et al.2025[56] | HIIT (14) | volleyball | Male | 45.55(5.34) | 8 | 20.7(1.4) | China |
|  | SIT (14) |  | Male | 46.93(4.25) | 8 | 19.8(2.67) |  |
| Wells et al.2014[57] | HIIT (8) | soccer | Male | 57.6(5.4) | 8 | 21.3(2.1) | UK |
|  | CG (8) |  | Male | 57.1(3.6) | 8 | 21.3(2.1) |  |
| Xu et al.2025[58] | SIT (8) | Karate | Male | 41.9(2.7) | 8 | 21.4(2.2) | China |
|  | RT (8) |  | Male | 42.3(2.8) | 8 | 21.7(2.4) |  |
|  | CG (8) |  | Male | 41.8(2.6) | 8 | 21.2(2.4) |  |
| Yang et al.2024[59] | SIT (10) | soccer | Male | 50.2(2.3) | 6 | 22.4(1.6) | China |
|  | RT (10) |  | Male | 50.1(2.2) | 6 | 21.2(1.4) |  |
|  | CG (10) |  | Male | 50.1(2.6) | 6 | 22.2(1.9) |  |
| zoll et al.2006[60] | AE (6) | distance runners | Male | 64.3(1.2) | 6 | 31.3(3.1) | France |
|  | IHT (9) |  | Male | 59.4(1.0) | 6 | 30.3(2.1) |  |

AE: Aerobic Exercise; BFR: Blood Flow Restriction (or Aquatic Exercise); CG: Control Group; IHT: Intermittent Hypoxic Training; SSG: Small-Sided Games; RT: Resistance Training; SIT: Sprint Interval Training; HIIT: High-Intensity Interval Training; POL: Polarized Training; HVT: High-Volume Training; MICT: Moderate-Intensity Continuous Training; THR: Threshold Training; NR: Not Report.

**Table S3.** Characteristics of included studies — Dose-Response Analysis.

| Studyname | Interventions (n) | Baseline | length (week) | Age  (mean±sd) | Region | Sports items | Gender |
| --- | --- | --- | --- | --- | --- | --- | --- |
| aagaard et al.2011[1] | RT (7) | 73.5(8.2) | 16 | 19.5(0.8) | Denmark | cyclists | Male |
|  | CG (7) | 71.5(6.0) | 16 | 19.5(0.8) |  |  | Male |
| Bicer et al.2020[3] | RT (13) | 37.85(4.34) | 8 | 27.08(3.99) | Turkey | volleyball | Male |
|  | CG (13) | 51.81(19.03) | 8 | 27.46(4.05) |  |  | Male |
| Bilici et al.2026[4] | HIIT (10) | 37.40(4.86) | 12 | 16.00(1.05) | Turkey | volleyball | Female |
|  | HIIT (11) | 36.73(2.37) | 12 | 16.18(1.17) |  |  | Female |
|  | CG (11) | 37.82(3.49) | 12 | 16.64(1.29) |  |  | Female |
| Fernandez et al.2012[13] | HIIT (11) | 56.3(4.0) | 6 | 22.6(4.8) | Germany | tennis | Male |
|  | SIT (12) | 55.6(5.0) | 6 | 21.2(5.1) |  |  | Male |
|  | CG (9) | 57.3(4.0) | 6 | 22.1(3.3) |  |  | Male |
| Gantois et al.2022[14] | SIT (10) | 50.39(4.30) | 6 | 21.6(3.34) | Brazil | volleyball | Male |
|  | CG (9) | 48.84(5.89) | 6 | 21.33(2.91) |  |  | Male |
| Jarstad et al.2019[16] | HIIT (7) | 50.6(5.4) | 10 | 28(5) | Norway | endurance athletes | Mix |
|  | MICT (7) | 50.9(7.1) | 10 | 28(5) |  |  | Mix |
|  | CG (6) | 54.5(7.3) | 10 | 28(5) |  |  | Mix |
| Jin et al.2025.March[19] | HIIT (12) | 51.48(3.85) | 4 | 19.94(1.4) | China | swimmers | Mix |
|  | CG (12) | 49.40(3.75) | 4 | 21.17(1.6) |  |  | Mix |
| Jin et al.2025.January[18] | SIT (10) | 67.2(5.0) | 6 | 19(1) | China | distance runners | Male |
|  | AE (10) | 68.8(7.0) | 6 | 20(1) |  |  | Male |
| kaynak et al.2017[20] | SIT (9) | 50(3.6) | 6 | 21.2(1.3) | Turkey | volleyball | Male |
|  | CG (9) | 50.4(4) | 6 | 21.2(1.6) |  |  | Male |
| Kim et al.2011[23] | SIT (11) | 49.84(4.08) | 8 | 20.00(1.10) | Korea | Judo | Male |
|  | CG (19) | 46.63(4.99) | 8 | 19.94(1.16) |  |  | Male |
| Kirchenberger et al.2021[24] | HIIT (10) | 58.4(3.9) | 8 | 15.4(1.3) | Germany | Rowers | Male |
|  | MICT (7) | 58.4(5.7) | 8 | 15.3(1.2) |  |  | Male |
| Kristoffersen et al.2019[25] | SIT (16) | 63.3(5.9) | 6 | 29.6(0.6) | Norway | cyclists | Mix |
|  | RT (16) | 61.1(6.0) | 6 | 29.6(0.6) |  |  | Mix |
| Liu et al.2023[28] | SIT (8) | 54.6(3.6) | 4 | 25(6) | Korea | swimmers | Male |
|  | CG (8) | 54.5(3.5) | 4 | 25(6) |  |  | Male |
| Maggioni et al.2019[29] | SSG (12) | 57.4(6.7) | 8 | 19(1) | Italy | Basketball | NR |
|  | SIT (9) | 55.7(7.2) | 8 | 19(1) |  |  | NR |
|  | CG (9) | 56.7(2.8) | 8 | 19(1) |  |  | NR |
| Mallol et al.2019[61] | HIIT (8) | 42.4(5.2) | 4 | 42.9(12.1) | Australia | triathletes | Mix |
|  | CG (8) | 43.1(4.5) | 4 | 37.2(13.3) |  |  | Mix |
| Millet et al.2002[32] | RT (7) | 69.7(3.60) | 14 | 24.3(5.2) | France | triathletes | NR |
|  | CG (8) | 67.6(6.4) | 14 | 21.4(2.1) |  |  | NR |
| MonKS et al.2017[33] | HIIT (16) | 56.1(1.38) | 4 | 18-22 | Korea | Taekwondo | Mix |
|  | AE (17) | 51.5(1.34) | 4 | 18-22 |  |  | Mix |
| Morais et al.2025[34] | HIIT (12) | 45.21(2.06) | 6 | 13.6(0.3) | Spain | tennis | Male |
|  | SIT (12) | 45.50(1.85) | 6 | 13.6(0.3) |  |  | Male |
| Muñoz et al.2021[35] | HIIT (10) | 37.50(6.04) | 8 | 23.6(2.8) | Chile | Field Hockey | Female |
|  | CG (10) | 35.25(5.50) | 8 | 23.6(3.0) |  |  | Female |
| ouergui et al.2020[36] | SIT (13) | 50.9(3.4) | 4 | 16(1) | Tunisia | Taekwondo | Mix |
|  | SSG (13) | 51.1(4.9) | 4 | 16(1) |  |  | Mix |
|  | CG (13) | 48.3(3.2) | 4 | 16(1) |  |  | Mix |
| ØVRETVEIT et al.2019[37] | HIIT (6) | 52.7(5.3) | 6 | 30.3(4.0) | Norway | BJJ | Male |
|  | CG (6) | 53.3(1.2) | 6 | 30.3(4.0) |  |  | Male |
| Ronnestad et al.2010.August[45] | RT (6) | 65.2(2.2) | 25 | 29(3) | Norway | cyclists | Male |
|  | CG (6) | 67.3(2.7) | 25 | 31(3) |  |  | Mix |
| Rønnestad et al.2010.November[46] | RT (11) | 66.8(1.6) | 12 | 27(2) | Norway | cyclists | Male |
|  | CG (9) | 65.9(2.0) | 12 | 30(2) |  |  | Mix |
| Sandbakk et al.2013[47] | HIIT (7) | 68(8.4) | 8 | 17.5(0.4) | Norway | SKIERS | Mix |
|  | HIIT (7) | 67.4(7.7) | 8 | 17.5(0.4) |  |  | Mix |
|  | AE (7) | 66.6(9.6) | 8 | 17.5(0.4) |  |  | Mix |
| Sheykhlouvand et al.2024[48] | SSG (10) | 51.84(2.6) | 6 | 26.2(4.0) | Iran | soccer | Male |
|  | SIT (10) | 51.57(2.6) | 6 | 25.8(3.5) |  |  | Male |
| Song et al.2024[49] | SIT (10) | 48.8(2.5) | 6 | 19.8(1.3) | Iran | Taekwondo | Male |
|  | SSG (10) | 46.4(3.0) | 6 | 19.8(1.3) |  |  | Male |
|  | CG (10) | 46.6(2.9) | 6 | 19.8(1.3) |  |  | Male |
| Wei et al.2025[56] | SIT (14) | 46.93(4.25) | 8 | 19.8(2.67) | China | volleyball | Male |
|  | HIIT (14) | 45.55(5.34) | 8 | 20.7(1.4) |  |  | Male |
| Wells et al.2014[57] | HIIT (8) | 57.6(5.4) | 8 | 21.3(2.1) | UK | soccer | Male |
|  | CG (8) | 57.1(3.6) | 8 | 21.3(2.1) |  |  | Male |
| Xu et al.2025[58] | RT (8) | 41.9(2.7) | 8 | 21.4(2.2) | China | Taekwondo | Male |
|  | SIT (8) | 42.3(2.8) | 8 | 21.7(2.4) |  |  | Male |
|  | CG (8) | 41.8(2.6) | 8 | 21.2(2.4) |  |  | Male |
| Yang et al.2024[59] | RT (10) | 50.1(2.2) | 6 | 21.2(1.4) | China | soccer | Male |
|  | SIT (10) | 50.2(2.3) | 6 | 22.4(1.6) |  |  | Male |
|  | CG (10) | 50.1(2.6) | 6 | 22.2(1.9) |  |  | Male |
| Yu et al.2025[62] | RT (16) | 64.92(5.18) | 8 | 16.81(0.61) | China | endurance runners | Male |
|  | RT (16) | 65.84(5.95) | 8 | 16.75(0.68) |  |  | Male |

AE: Aerobic Exercise; BFR: Blood Flow Restriction (or Aquatic Exercise); CG: Control Group; IHT: Intermittent Hypoxic Training; SSG: Small-Sided Games; RT: Resistance Training; SIT: Sprint Interval Training; HIIT: High-Intensity Interval Training; POL: Polarized Training; HVT: High-Volume Training; MICT: Moderate-Intensity Continuous Training; THR: Threshold Training; NR: Not Report.

**4.Network Geometry**

**Table S4.** Network geometry summary.

| **Characteristic** | **Value** |
| --- | --- |
| Number of studies | 60 |
| Number of treatment arms (after pooling) | 135 |
| Number of treatments (nodes) | 12 |
| Number of pairwise comparisons | 36 |
| Number of study designs | 14 |
| Multi-arm studies | 4 (3-arm: 3; 4-arm: 1) |
| Reference treatment | CG (Control group) |

**Figure S1.** Network plot

**Figure S2.**SUCRA rankings plot

**5.Risk of Bias Assessment**

**Figure S3.** The detail of risk of bias.

**Figure S4.** The summary of risk of bias.

**6. Network Meta-Analysis Main Results**

**Table S5.** Random-effects treatment estimates vs. CG and SUCRA rankings

| Treatment | MD vs CG | 95% CrI | SUCRA | Rank |
| --- | --- | --- | --- | --- |
| BFR | 3.93 | [0.60, 7.27] | 0.857 | 1 |
| HIIT | 2.71 | [1.51, 3.93] | 0.726 | 2 |
| IHT | 2.74 | [0.60, 4.84] | 0.717 | 3 |
| SSG | 2.56 | [0.31, 4.79] | 0.681 | 4 |
| SIT | 2.49 | [1.25, 3.75] | 0.676 | 5 |
| POL | 2.28 | [-3.79, 8.41] | 0.598 | 6 |
| THR | 0.49 | [-5.32, 6.49] | 0.355 | 7 |
| RT | 0.82 | [-0.88, 2.55] | 0.352 | 8 |
| HVT | 0.48 | [-4.16, 5.11] | 0.340 | 9 |
| AE | 0.33 | [-1.49, 2.16] | 0.256 | 10 |
| MICT | 0.16 | [-2.66, 2.91] | 0.253 | 11 |
| CG | — | — | 0.191 | 12 |

Heterogeneity: tau = 1.22 (posterior mean). Five treatments significantly superior to CG: BFR, HIIT, IHT, SIT, SSG (95% CrI excludes zero).

**Figure S5**. Forest plot

**Figure S6.** League plot

**7. Convergence Diagnostics**

All model parameters achieved R-hat < 1.05 (range: 1.000–1.004), confirming adequate convergence of all four MCMC chains.

**Figure S7**. Convergence diagnostic plots

**8.Inconsistency Assessment**

DIC comparison: Consistency model DIC = 99.37; UME model DIC = 98.39; Difference = -0.98 (< 5, indicating good global consistency).

**Table S6.** Node-splitting results — significantly inconsistent comparisons (p < 0.05).

| Comparison | Direct MD [95% CrI] | Indirect MD [95% CrI] | Network MD [95% CrI] | p-value | Assessment |
| --- | --- | --- | --- | --- | --- |
| AE vs IHT | 1.58 [-0.01, 3.09] | 5.84 [2.64, 8.89] | 2.40 [0.84, 3.89] | 0.023 | Inconsistent |
| HIIT vs IHT | 2.49 [-0.56, 5.37] | -1.30 [-3.42, 0.85] | 0.00 [-1.86, 1.89] | 0.050 | Inconsistent |

**Table S7.** (continued). Node-splitting — consistent comparisons (p ≥ 0.05).

| Comparison | Direct MD [95% CrI] | Indirect MD [95% CrI] | Network MD [95% CrI] | p-value | Assessment |
| --- | --- | --- | --- | --- | --- |
| AE vs HIIT | 3.29 [1.48, 4.98] | 0.48 [-1.92, 3.05] | 2.41 [0.89, 3.86] | 0.085 | Consistent |
| RT vs SIT | 0.82 [-1.80, 3.43] | 2.81 [-0.07, 5.56] | 1.67 [-0.24, 3.52] | 0.309 | Consistent |
| CG vs RT | 0.48 [-1.40, 2.38] | 3.52 [-2.15, 9.12] | 0.81 [-0.90, 2.54] | 0.314 | Consistent |
| CG vs SSG | 5.29 [-0.88, 11.59] | 2.19 [-0.25, 4.50] | 2.54 [0.35, 4.77] | 0.352 | Consistent |
| CG vs IHT | 5.79 [-1.67, 13.30] | 2.47 [0.19, 4.68] | 2.71 [0.56, 4.81] | 0.400 | Consistent |
| BFR vs HIIT | -1.16 [-4.99, 2.69] | -2.68 [-9.73, 4.30] | -1.22 [-4.28, 1.86] | 0.717 | Consistent |
| AE vs BFR | 3.30 [-0.85, 7.40] | 4.32 [-1.13, 9.74] | 3.63 [0.51, 6.75] | 0.773 | Consistent |
| SIT vs SSG | -0.45 [-3.92, 3.21] | 0.17 [-2.90, 3.09] | 0.06 [-2.13, 2.28] | 0.791 | Consistent |
| HIIT vs MICT | -1.64 [-4.71, 1.45] | -2.41 [-8.25, 3.33] | -2.54 [-5.19, 0.08] | 0.819 | Consistent |
| HIIT vs SIT | -0.04 [-2.33, 2.29] | -0.34 [-2.20, 1.53] | -0.21 [-1.65, 1.21] | 0.835 | Consistent |
| AE vs SIT | 2.70 [-3.74, 9.24] | 2.08 [-0.03, 4.14] | 2.18 [0.19, 4.12] | 0.842 | Consistent |
| CG vs HIIT | 2.52 [1.09, 3.91] | 2.80 [0.36, 5.31] | 2.71 [1.46, 3.91] | 0.851 | Consistent |
| CG vs SIT | 2.56 [1.03, 4.02] | 2.34 [-0.08, 4.76] | 2.48 [1.23, 3.72] | 0.860 | Consistent |
| AE vs CG | -0.56 [-6.10, 5.00] | -0.30 [-2.26, 1.61] | -0.31 [-2.09, 1.50] | 0.935 | Consistent |
| AE vs MICT | 0.06 [-5.59, 5.61] | -0.17 [-3.59, 3.17] | -0.13 [-3.14, 2.73] | 0.950 | Consistent |
| CG vs MICT | -1.01 [-5.88, 3.85] | -1.10 [-4.50, 2.38] | 0.17 [-2.62, 2.94] | 0.975 | Consistent |
| HIIT vs SSG | -0.17 [-2.66, 2.42] | -0.15 [-3.68, 3.61] | -0.16 [-2.24, 1.96] | 0.995 | Consistent |

BFR, HIIT, and SIT demonstrated robust significant effects vs. CG across both sensitivity analyses. IHT was conditionally significant (present in the primary analysis and Sensitivity 1, absent by design from Sensitivity 2). SSG lost statistical significance when IHT interventions were excluded (Sensitivity 2), suggesting its effect estimate is partly dependent on indirect evidence routed through IHT nodes in the network.

**Figure S8.** Node-splitting plot

**9.Sensitivity Analyses**

**Figure S9.** Sensitivity analysis summary

Figure S9 summarizes the primary analysis and both sensitivity analyses: Blue circles = primary analysis (n = 60); Red triangles = Sensitivity 1, excluding high risk of bias studies (n = 54); Green squares = Sensitivity 2, excluding IHT interventions (n = 50).

**Table S8.** Robustness assessment of treatments with significant effects vs. CG.

| Treatment vs CG | Primary (n=60) | Sens.1: No high RoB (n=54) | Sens.2: No IHT (n=50) | Robust? |
| --- | --- | --- | --- | --- |
| BFR | 3.92 [0.60, 7.27] ✓ | ✓ | ✓ | Yes |
| HIIT | 2.71 [1.51, 3.93] ✓ | ✓ | ✓ | Yes |
| SIT | 2.49 [1.25, 3.75] ✓ | ✓ | ✓ | Yes |
| IHT | 2.75 [0.60, 4.84] ✓ | ✓ | — (excluded) | Conditional |
| SSG | 2.57 [0.31, 4.79] ✓ | ✓ | Crosses zero | Unstable |

**Note:**SSG lost statistical significance when IHT interventions were excluded (Sensitivity 2), suggesting its effect estimate is partly dependent on indirect evidence routed through IHT nodes in the network.

**10. Network Meta-Regression**

**Table S9.**Network meta-regression ( age, baseline VO2max, intervention duration)

| **Covariate** | **DIC** | **Residual deviance** | **Shared beta**  **(median and 95%CrI)** | **SD** |
| --- | --- | --- | --- | --- |
| Age (years) | 219.9 | 122.5 | -1.09 (-3.04, 0.91) | 1.65 (1.13, 2.29) |
| Baseline VO2max (mL/kg/min) | 220.3 | 122.6 | -1.22  (-2.98, 0.57) | 1.63 (1.12, 2.29) |
| Intervention  length (weeks) | 221.3 | 123.2 | 0.42  (-1.19, 1.96) | 1.67 (1.15, 2.36) |

**Note:** DIC: deviance information criterion; SD: standard deviation

**11. Subgroup Analysis by Sport Type**

**Table S10.** SUCRA rankings — Endurance subgroup (25 studies, Bayesian NMA).

| Rank | Treatment | SUCRA |
| --- | --- | --- |
| 1 | IHT | 0.821 |
| 2 | BFR | 0.671 |
| 3 | MICT | 0.623 |
| 4 | SIT | 0.557 |
| 5 | CG | 0.508 |
| 6 | POL | 0.448 |
| 7 | RT | 0.437 |
| 8 | AE | 0.423 |
| 9 | HIIT | 0.416 |
| 10 | HVT | 0.338 |
| 11 | THR | 0.258 |

No treatment showed statistically significant difference vs. CG (all 95% CrI cross zero). All 12 node-splitting comparisons consistent. Tau = 1.22.

**Figure S10.** SUCRA rankings plot

**Table S11.** Pairwise meta-analysis vs. CG — subgroups with sparse networks (random-effects, DerSimonian-Laird).

| Subgroup | Treatment | k | MD [95% CI] | I² | p-value |
| --- | --- | --- | --- | --- | --- |
| Team Ball (18) | SIT | 5 | 2.70 [1.17, 4.23] | 0% | 0.001 |
|  | RT | 2 | 2.90 [0.69, 5.11] | 0% | 0.010 |
|  | HIIT | 4 | 2.85 [-1.57, 7.28] | 1% | 0.207 |
|  | SSG | 1 | 5.30 [0.00, 10.60] | — | 0.050 |
| Water Sports (7) | HIIT | 2 | 4.69 [1.76, 7.62] | 0% | 0.002 |
|  | SIT | 1 | 2.50 [-0.87, 5.87] | — | 0.145 |
| Combat/Racket (10) | SIT | 5 | 2.59 [1.20, 3.99] | 0% | <0.001 |
|  | HIIT | 3 | 2.46 [0.75, 4.17] | 0% | 0.005 |
|  | RT | 1 | -0.30 [-2.88, 2.28] | — | 0.820 |

k = number of studies with direct CG comparison. BFR, AE, HVT, and MICT had no direct CG comparisons in these subgroups. SIT and HIIT were the only treatments with consistent, statistically significant benefits vs. CG across multiple sport subgroups.

**Figure S11.** Forest plots

**12. Publication Bias**

Publication bias was assessed using a comparison-adjusted funnel plot with Egger's regression test.

**Figure S12.** Comparison-adjusted funnel plot

**13.Grade summary of all studies**

Based on the CINeMA online guidelines ([https://cinema.ispm.unibe.ch/](https://cinema.ispm.unibe.ch/?utm_source=chatgpt.com)), we evaluated the confidence of evidence for the maximal oxygen uptake (VO₂max) outcome:

**13.1 Within-study Bias**
Each included study was rated for risk of bias as low (RoB2 1 point), moderate (RoB2 2 points), or high (RoB2 3 points). The rule applied was the average RoB. No downgrade occurred for “no concerns”; one level was downgraded for “some concerns,” and two levels for “major concerns.”

**13.2 Reporting Bias (Publication Bias and Selective Reporting)**
To minimize publication bias, we examined the reference lists of recent relevant studies. Publication bias was assessed using comparison-adjusted funnel plots, **Begg's** and Egger’s test (Section 12; Figure S12). The results indicated no statistically significant bias (p > 0.05), suggesting a low risk of publication bias. Furthermore, no high risk of selective reporting was identified; therefore, this domain was not downgraded.

**13.3 Indirectness**
Indirectness assessment was conducted by analyzing the clinical and methodological relevance of the included studies to the research question. All included studies strictly adhered to the predefined PICOS criteria: they involved healthy athletes, employed various exercise training modalities, established appropriate control groups (maintaining baseline training), and used maximum oxygen uptake (VO₂max) as the primary outcome measure. Although variations in specific sports disciplines and baseline cardiorespiratory fitness levels across studies may introduce some indirectness, this was effectively addressed through predefined subgroup analyses (e.g., endurance vs. team sports) and rigorous sensitivity analyses.

**13.4 Imprecision**
In this network meta-analysis, maximum oxygen uptake (VO₂max) was analyzed as a continuous variable. Because VO₂max is universally reported in standardized units (mL/kg/min) across the included trials, the mean difference (MD) was employed as the effect size indicator, calculated via change scores (endpoint minus baseline values). Recognizing the lack of a universally established minimum clinically important difference (MCID) for VO₂max improvements across diverse athletic disciplines, the null value (0 mL/kg/min) was established as the absolute reference threshold to evaluate the efficacy of the exercise interventions.

**13.5 Heterogeneity**
Beyond statistical heterogeneity, physiological and methodological heterogeneity arising from variations in baseline cardiorespiratory fitness, specific athletic disciplines, foundational training loads, and the standardization of exercise interventions was rigorously evaluated when assigning heterogeneity ratings. Comparisons exhibiting prediction intervals that extended substantially beyond their corresponding credible intervals—indicating variability of practical relevance to athletic training prescription—were downgraded accordingly, as detailed in the individual comparison assessments in Table S12.

**13.6 Incoherence**
Incoherence was addressed by p-value thresholds: no downgrade for p > 0.1, one level for p 0.05–0.1, and two levels for p < 0.05.

**13.7 Summarizing Judgments Across CINeMA Domains**
The CINeMA framework assesses confidence in evidence across six domains: within-study bias, reporting bias, indirectness, imprecision, heterogeneity, and incoherence. These judgments are summarized into an overall confidence rating using the GRADE approach (high, moderate, low, or very low). As a single issue may impact multiple domains (e.g., indirectness due to intransitivity or heterogeneity affecting both imprecision and bias), all domains are considered together to avoid redundant downgrading and ensure a balanced assessment.

**Figure S13.** Network plot of study limitations of the included studies

The colors in the circles indicate the percentage of low RoB studies (green), moderate RoB studies (yellow), high RoB studies (red) about each physical activity type. Edge width by the number of studies. Edge color by average RoB. The colors of the lines indicate the summative RoB assessment of each comparison. Low RoB is green, moderate RoB is yellow, high RoB is red.

**Table S12.** The confidence of evidence using the CINeMA

| **Comparison** | **Number of studies** | **Within-study bias** | **Reporting bias** | **Indirectness** | **Imprecision** | **Heterogeneity** | **Incoherence** | **Confidence rating** | **Reason**  **(s) for downgrading** |
| --- | --- | --- | --- | --- | --- | --- | --- | --- | --- |
| AE:BFR | 2 | No concerns | Low risk | No concerns | No concerns | Major concerns | No concerns | Low | ["Heterogeneity"] |
| **AE:CG** | 1 | Some concerns | Low risk | No concerns | Major concerns | No concerns | No concerns | **Very low** | ["Within-study bias","Imprecision"] |
| AE:HIIT | 7 | Some concerns | Low risk | No concerns | No concerns | Major concerns | No concerns | Low | ["Within-study bias","Heterogeneity"] |
| AE:IHT | 7 | No concerns | Low risk | No concerns | No concerns | Major concerns | Major concerns | Very low | ["Heterogeneity","Incoherence"] |
| AE:MICT | 2 | Some concerns | Low risk | No concerns | Major concerns | No concerns | No concerns | Very low | ["Within-study bias","Imprecision"] |
| AE:SIT | 1 | Some concerns | Low risk | No concerns | No concerns | Major concerns | No concerns | Very low | ["Within-study bias","Heterogeneity"] |
| BFR:HIIT | 2 | No concerns | Low risk | No concerns | Major concerns | No concerns | No concerns | Low | ["Imprecision"] |
| **HIIT:CG** | 12 | No concerns | Low risk | No concerns | No concerns | Major concerns | No concerns | **Low** | ["Heterogeneity"] |
| **IHT:CG** | 1 | Some concerns | Low risk | No concerns | No concerns | Major concerns | No concerns | **Very low** | ["Within-study bias","Heterogeneity"] |
| **MICT:CG** | 1 | Some concerns | Low risk | No concerns | Major concerns | No concerns | No concerns | **Very low** | ["Within-study bias","Imprecision"] |
| **RT:CG** | 7 | No concerns | Low risk | No concerns | Major concerns | No concerns | No concerns | **Low** | ["Imprecision"] |
| **SIT:CG** | 11 | No concerns | Low risk | No concerns | No concerns | Major concerns | No concerns | **Low** | ["Heterogeneity"] |
| **SSG:CG** | 1 | No concerns | Low risk | No concerns | No concerns | Major concerns | No concerns | **Low** | ["Heterogeneity"] |
| HIIT:HVT | 2 | Some concerns | Low risk | No concerns | Major concerns | No concerns | Major concerns | Very low | ["Within-study bias","Imprecision","Incoherence"] |
| HIIT:IHT | 2 | Some concerns | Low risk | No concerns | Major concerns | No concerns | No concerns | Very low | ["Within-study bias","Imprecision"] |
| HIIT:MICT | 3 | Some concerns | Low risk | No concerns | Major concerns | No concerns | No concerns | Very low | ["Within-study bias","Imprecision"] |
| HIIT:POL | 1 | Some concerns | Low risk | No concerns | Major concerns | No concerns | No concerns | Very low | ["Within-study bias","Imprecision"] |
| HIIT:SIT | 4 | Some concerns | Low risk | No concerns | Major concerns | No concerns | No concerns | Very low | ["Within-study bias","Imprecision"] |
| HIIT:SSG | 2 | Some concerns | Low risk | No concerns | Major concerns | No concerns | No concerns | Very low | ["Within-study bias","Imprecision"] |
| HIIT:THR | 1 | Some concerns | Low risk | No concerns | Major concerns | No concerns | No concerns | Very low | ["Within-study bias","Imprecision"] |
| HVT:POL | 1 | Some concerns | Low risk | No concerns | Major concerns | No concerns | No concerns | Very low | ["Within-study bias","Imprecision"] |
| HVT:THR | 1 | Some concerns | Low risk | No concerns | Major concerns | No concerns | No concerns | Very low | ["Within-study bias","Imprecision"] |
| POL:THR | 2 | Some concerns | Low risk | No concerns | Major concerns | No concerns | Major concerns | Very low | ["Within-study bias","Imprecision","Incoherence"] |
| RT:SIT | 3 | No concerns | Low risk | No concerns | Major concerns | No concerns | No concerns | Low | ["Imprecision"] |
| SIT:SSG | 2 | No concerns | Low risk | No concerns | Major concerns | No concerns | No concerns | Low | ["Imprecision"] |

# **14.Dose-Response Analysis**

**14.1 Quantification of Training Dose**

Due to heterogeneity in exercise intensity reporting across included RCTs—ranging from absolute mechanical workload to relative physiological thresholds (%HRmax, %VO₂max) or qualitative descriptors ("all-out")—a hierarchical data extraction strategy was developed to standardize the quantification of training dose in METs.

**Tier 1:** Direct matching. For studies reporting absolute mechanical workload (e.g., power output in watts, absolute speed), the precise MET value was assigned using the 2024 Compendium of Physical Activities [63].

**Tier 2:** Standardized MET anchoring matrix. For studies reporting only relative intensity, the following standardized MET values were applied. Baseline values were derived from the 2024 Compendium, with intensity-specific boundaries calibrated against consensus thresholds from exercise physiology literature.

**Table S13.** Standardized MET assignment rules for dose-response modelling

| Training Modality | Intensity Description / Physiological Anchor | Assigned MET | Key Reference |
| --- | --- | --- | --- |
| SIT (Sprint Interval Training) | All-out / Supramaximal (>100% VO₂max) | 15.0 | Gibala et al [64]. |
| HIIT (High-Intensity Interval Training) | 90–95% HRmax / Severe intensity domain | 12.0 | Buchheit & Laursen [65] |
| AE (High-Intensity Aerobic Exercise) | 80–90% HRmax | 5.5 | Seiler [66]; ACSM [67]. |
| SSG (Small-Sided Games) | 75–85% HRmax / Sport-specific | 9.5 | Hill-Haas et al [68]. |
| MICT (Moderate-Intensity Continuous Training) | 70–80% HRmax | 8.0 | Seiler [66]; ACSM [67]. |
| RT (Heavy Resistance Training) | ≥80% 1RM | 6.0 | ACSM [69]. |
| CG (Control Group) | Usual training maintenance | 0 | — |

**Note:** MET values were used as external-dose anchors for harmonizing heterogeneous supplementary training interventions, not as measured internal physiological load. When studies reported absolute workload or directly matchable activities, values were matched to the 2024 Adult Compendium of Physical Activities. When studies reported relative intensity descriptors, modality-level anchors were applied using established exercise-physiology references. For supramaximal SIT and resistance-based exercise, MET-based scaling may underestimate internal physiological stimulus, anaerobic contribution, neuromuscular demand, and recovery cost. Study-arm-level dose values used in the model are reported in Supplementary Table S19. MET, metabolic equivalent of task; SIT, sprint interval training; HIIT, high-intensity interval training; AE, vigorous aerobic exercise; SSG, small-sided games; MICT, moderate-intensity continuous training; RT, resistance training; CG, control group; HRmax, maximum heart rate; 1RM, one-repetition maximum. Assigned MET values were calibrated against the 2024 Compendium of Physical Activities and corresponding physiological consensus guidelines.

The specific dose calculation method is detailed in Section 14.3.

**14.2 Note on SIT Dose Characteristics**

In the dose-response models, SIT sometimes showed a relatively low computed MET-min/week dose with favourable estimated effects. This should not be interpreted as a low physiological stimulus. MET-min/week is an external exposure metric and may underrepresent supramaximal exercise because it does not fully capture anaerobic contribution, neuromuscular demand, or recovery cost.

**14.3**  **Standardized MET assignment rules for supplementary training modalities.**

A subset of 31 studies with quantified supplementary dose data was analyzed using a Bayesian dose-response NMA with the quadratic dose-response model selected by DIC (Section 14.7). Weekly dose (METs-min/week) was calculated as METs × session duration × frequency ×average adherence rate, representing net additional training beyond regular sport-specific practice.

- **Agent:** The agent level.
- **MET_min:** The energy expenditure per min in the study.
- **Min_session：**The duration of a single exercise intervention.
- **Frequency:** The number of days participants engaged in physical activity.
- **Exact_dose:** The exact estimated METs accumulated per week by participants.
- **Residual_dose:** The difference between the exact dose and the approximated dose.
- **Dose:** The dose group by approximation.
- **CG:** Control group

| studyID | Agent | MET_min | min_session | MET_session | frequency | exact_dose | residual_dose | dose | Average adherence rate (%) | n |
| --- | --- | --- | --- | --- | --- | --- | --- | --- | --- | --- |
| 1 | RT | 3.5 | 90 | 315 | 2 | 504 | 4 | 500 | 80% | 20 |
| 1 | CG | 0 | 0 | 0 | 0 | 0 | 0 | 0 | 0 | 20 |

**Note:** MET values were assigned using a two-step approach. When studies reported absolute workload or directly matchable activities, values were matched to the 2024 Adult Compendium of Physical Activities. When studies reported relative intensity descriptors, standardized modality-level anchors were assigned using established exercise-physiology references. These values were used to calculate weekly supplementary dose as MET value × session duration × weekly frequency × adherence.

**Table S19. Study-arm-level data used in the dose-response network meta-analysis.**

| **Study** | **Study ID** | **Agent** | **Model dose (MET-min/week)** | **Original dose (MET-min/week)** | **Baseline VO2max** | **Change (y)** | **SD** | **SE** | **n** | **Length (weeks)** | **Age** | **Country/Region** | **Sport** | **Sex** |
| --- | --- | --- | --- | --- | --- | --- | --- | --- | --- | --- | --- | --- | --- | --- |
| aagaard et al.2011 | 1 | RT | 700 | 700 | 73.5（8.2） | 1.50 | 7.35 | 2.78 | 7 | 16 | 19.5(0.8) | Denmark | cyclists | Male |
| aagaard et al.2011 | 1 | CG | 0 | 0 | 71.5（6.0） | 1.50 | 5.24 | 1.98 | 7 | 16 | 19.5(0.8) | Denmark | cyclists | Male |
| Bicer et al.2020 | 2 | RT | 750 | 740 | 37.85（4.34） | 4.65 | 7.70 | 2.14 | 13 | 8 | 27.08(3.99) | Turkey | volleyball | Male |
| Bicer et al.2020 | 2 | CG | 0 | 0 | 51.81（19.03） | -0.85 | 19.24 | 5.34 | 13 | 8 | 27.46(4.05) | Turkey | volleyball | Male |
| Bilici et al.2026 | 3 | HIIT | 550 | 550 | 37.40(4.86) | 0.90 | 4.87 | 1.54 | 10 | 12 | 16.00(1.05) | Turkey | volleyball | Female |
| Bilici et al.2026 | 3 | HIIT | 650 | 650 | 36.73(2.37) | 1.45 | 2.54 | 0.77 | 11 | 12 | 16.18(1.17) | Turkey | volleyball | Female |
| Bilici et al.2026 | 3 | CG | 0 | 0 | 37.82(3.49) | 0.18 | 3.35 | 1.01 | 11 | 12 | 16.64(1.29) | Turkey | volleyball | Female |
| Fernandez et al.2012 | 4 | HIIT | 500 | 486 | 56.3(4.0) | 2.80 | 3.58 | 1.08 | 11 | 6 | 22.6(4.8) | Germany | tennis | Male |
| Fernandez et al.2012 | 4 | SIT | 150 | 120 | 55.6(5.0) | 3 | 4.35 | 1.26 | 12 | 6 | 21.2(5.1) | Germany | tennis | Male |
| Fernandez et al.2012 | 4 | CG | 0 | 0 | 57.3(4.0) | 0.10 | 3.90 | 1.30 | 9 | 6 | 22.1(3.3) | Germany | tennis | Male |
| Gantois et al.2022 | 5 | SIT | 50 | 52.70 | 50.39(4.30) | 1.91 | 4.64 | 1.47 | 10 | 6 | 21.6(3.34) | Brazil | volleyball | Male |
| Gantois et al.2022 | 5 | CG | 0 | 0 | 48.84(5.89) | -0.97 | 5.55 | 1.85 | 9 | 6 | 21.33(2.91) | Brazil | volleyball | Male |
| Jarstad et al.2019 | 6 | HIIT | 700 | 700 | 50.6(5.4) | 2.90 | 6.29 | 2.38 | 7 | 10 | 28(5) | Norway | endurance athletes | Mix |
| Jarstad et al.2019 | 6 | MICT | 1000 | 1000 | 50.9(7.1) | 2.40 | 7.15 | 2.70 | 7 | 10 | 28(5) | Norway | endurance athletes | Mix |
| Jarstad et al.2019 | 6 | CG | 0 | 0 | 54.5(7.3) | 1.20 | 7.73 | 3.16 | 6 | 10 | 28(5) | Norway | endurance athletes | Mix |
| Jin et al.2025 | 7 | HIIT | 1350 | 1350 | 51.48(3.85) | 3.55 | 4.47 | 1.29 | 12 | 4 | 19.94(1.4) | China | swimmers | Mix |
| Jin et al.2025 | 7 | CG | 0 | 0 | 49.40(3.75) | -1.21 | 3.65 | 1.05 | 12 | 4 | 21.17(1.6) | China | swimmers | Mix |
| Jin et al.2025 | 8 | SIT | 150 | 150 | 67.2（5.0） | 2.90 | 5.38 | 1.70 | 10 | 6 | 19（1) | China | distance runners | Male |
| Jin et al.2025 | 8 | AE | 700 | 720 | 68.8（7.0） | 0.10 | 7.05 | 2.23 | 10 | 6 | 20(1) | China | distance runners | Male |
| kaynak et al.2017 | 9 | SIT | 50 | 34.10 | 50(3.6) | 3.40 | 3.12 | 1.04 | 9 | 6 | 21.2(1.3) | Turkey | volleyball | Male |
| kaynak et al.2017 | 9 | CG | 0 | 0 | 50.4(4) | 0.20 | 4 | 1.33 | 9 | 6 | 21.2(1.6) | Turkey | volleyball | Male |
| Kim et al.2011 | 10 | SIT | 250 | 250 | 49.84(4.08) | 3.28 | 4.47 | 1.35 | 11 | 8 | 20.00(1.10) | Korea | Judo | Male |
| Kim et al.2011 | 10 | CG | 0 | 0 | 46.63(4.99) | 3.46 | 4.68 | 1.07 | 19 | 8 | 19.94(1.16) | Korea | Judo | Male |
| Kirchenberger et al.2021 | 11 | HIIT | 450 | 448 | 58.4(3.9) | 3.70 | 3.40 | 1.07 | 10 | 8 | 15.4(1.3) | Germany | Rowers | Male |
| Kirchenberger et al.2021 | 11 | MICT | 800 | 800 | 58.4(5.7) | -0.10 | 6.08 | 2.30 | 7 | 8 | 15.3(1.2) | Germany | Rowers | Male |
| Kristoffersen et al.2019 | 12 | SIT | 50 | 36 | 63.3（5.9） | 1.50 | 6.06 | 1.51 | 16 | 6 | 29.6（0.6） | Norway | cyclists | Mix |
| Kristoffersen et al.2019 | 12 | RT | 550 | 540 | 61.1（6.0） | 2.30 | 6.38 | 1.59 | 16 | 6 | 29.6（0.6） | Norway | cyclists | Mix |
| Liu et al.2023 | 13 | SIT | 130 | 135 | 54.6（3.6） | 3.20 | 3.42 | 1.21 | 8 | 4 | 25(6) | Korea | swimmers | Male |
| Liu et al.2023 | 13 | CG | 0 | 0 | 54.5（3.5） | 0.70 | 3.45 | 1.22 | 8 | 4 | 25(6) | Korea | swimmers | Male |
| Maggioni et al.2019 | 14 | SSG | 300 | 288 | 57.4(6.7) | 2.80 | 8.38 | 2.42 | 12 | 8 | 19(1) | Italy | Basketball | NR |
| Maggioni et al.2019 | 14 | SIT | 50 | 65 | 55.7(7.2) | 1.80 | 6.61 | 2.20 | 9 | 8 | 19(1) | Italy | Basketball | NR |
| Maggioni et al.2019 | 14 | CG | 0 | 0 | 56.7(2.8) | -2.50 | 3.63 | 1.21 | 9 | 8 | 19(1) | Italy | Basketball | NR |
| Mallol et al.2019 | 15 | HIIT | 350 | 352 | 42.4(5.2) | 2.80 | 5.64 | 1.99 | 8 | 4 | 42.9(12.1) | Australia | triathletes | Mix |
| Mallol et al.2019 | 15 | CG | 0 | 0 | 43.1(4.5) | -0.30 | 4.23 | 1.50 | 8 | 4 | 37.2(13.3) | Australia | triathletes | Mix |
| Millet et al.2002 | 16 | RT | 550 | 540 | 69.7(3.60) | -2.50 | 4.06 | 1.53 | 7 | 14 | 24.3(5.2) | France | triathletes | NR |
| Millet et al.2002 | 16 | CG | 0 | 0 | 67.6(6.4) | -0.30 | 6.04 | 2.14 | 8 | 14 | 21.4(2.1) | France | triathletes | NR |
| MonKS et al.2017 | 17 | HIIT | 250 | 240 | 56.1（1.38） | 4.70 | 1.49 | 0.37 | 16 | 4 | 18-22 | Korea | Taekwondo | Mix |
| MonKS et al.2017 | 17 | AE | 550 | 550 | 51.5（1.34） | 0.90 | 1.44 | 0.35 | 17 | 4 | 18-22 | Korea | Taekwondo | Mix |
| Morais et al.2025 | 18 | HIIT | 200 | 216 | 45.21(2.06) | 3.62 | 2.18 | 0.63 | 12 | 6 | 13.6(0.3) | Spain | tennis | Male |
| Morais et al.2025 | 18 | SIT | 50 | 45 | 45.50(1.85) | 3.13 | 1.93 | 0.56 | 12 | 6 | 13.6(0.3) | Spain | tennis | Male |
| Muñoz et al.2021 | 19 | HIIT | 150 | 154 | 37.50(6.04) | 2.70 | 5.64 | 1.78 | 10 | 8 | 23.6（2.8） | Chile | Field Hockey | Female |
| Muñoz et al.2021 | 19 | CG | 0 | 0 | 35.25(5.50) | 1.88 | 5.18 | 1.64 | 10 | 8 | 23.6（3.0） | Chile | Field Hockey | Female |
| ouergui et al.2020 | 20 | SIT | 150 | 125 | 50.9(3.4) | 4.10 | 3.18 | 0.88 | 13 | 4 | 16(1) | Tunisia | Taekwondo | Mix |
| ouergui et al.2020 | 20 | SSG | 100 | 95 | 51.1(4.9) | 5.30 | 4.42 | 1.23 | 13 | 4 | 16(1) | Tunisia | Taekwondo | Mix |
| ouergui et al.2020 | 20 | CG | 0 | 0 | 48.3(3.2) | 1.20 | 3.36 | 0.93 | 13 | 4 | 16(1) | Tunisia | Taekwondo | Mix |
| ØVRETVEIT et al.2019 | 21 | HIIT | 400 | 384 | 52.7（5.3） | 4.10 | 5.11 | 2.09 | 6 | 6 | 30.3(4.0) | Norway | BJJ | Male |
| ØVRETVEIT et al.2019 | 21 | CG | 0 | 0 | 53.3（1.2） | 0.80 | 1.37 | 0.56 | 6 | 6 | 30.3(4.0) | Norway | BJJ | Male |
| Rønnestad et al.2010 | 22 | RT | 400 | 400 | 65.2(2.2) | 8.70 | 2.84 | 1.16 | 6 | 25 | 29(3) | Norway | cyclists | Male |
| Rønnestad et al.2010 | 22 | CG | 0 | 0 | 67.3(2.7) | 6.10 | 2.92 | 1.19 | 6 | 25 | 31(3) | Norway | cyclists | Mix |
| Rønnestad et al.2010 | 23 | RT | 550 | 540 | 66.8(1.6) | 2.20 | 1.60 | 0.48 | 11 | 12 | 27（2） | Norway | cyclists | Male |
| Rønnestad et al.2010 | 23 | CG | 0 | 0 | 65.9(2.0) | 3.90 | 2.29 | 0.76 | 9 | 12 | 30（2） | Norway | cyclists | Mix |
| Sandbakk et al.2013 | 24 | HIIT | 600 | 603 | 68(8.4) | 2.20 | 7.85 | 2.97 | 7 | 8 | 17.5(0.4) | Norway | SKIERS | Mix |
| Sandbakk et al.2013 | 24 | HIIT | 1000 | 1000 | 67.4(7.7) | 2.60 | 7.80 | 2.95 | 7 | 8 | 17.5(0.4) | Norway | SKIERS | Mix |
| Sandbakk et al.2013 | 24 | AE | 1200 | 1200 | 66.6(9.6) | 0.20 | 7.70 | 2.91 | 7 | 8 | 17.5(0.4) | Norway | SKIERS | Mix |
| Sheykhlouvand et al.2024 | 25 | SSG | 500 | 500 | 51.84（2.6） | 2.03 | 2.39 | 0.76 | 10 | 6 | 26.2(4.0) | Iran | soccer | Male |
| Sheykhlouvand et al.2024 | 25 | SIT | 450 | 432 | 51.57（2.6） | 2.89 | 2.65 | 0.84 | 10 | 6 | 25.8(3.5) | Iran | soccer | Male |
| Song et al.2024 | 26 | SIT | 400 | 400 | 48.8(2.5) | 3.10 | 2.66 | 0.84 | 10 | 6 | 19.8（1.3） | Iran | Taekwondo | Male |
| Song et al.2024 | 26 | SSG | 350 | 324 | 46.4(3.0) | 2.10 | 3.10 | 0.98 | 10 | 6 | 19.8（1.3） | Iran | Taekwondo | Male |
| Song et al.2024 | 26 | CG | 0 | 0 | 46.6(2.9) | 0.50 | 3.06 | 0.97 | 10 | 6 | 19.8（1.3） | Iran | Taekwondo | Male |
| Wei et al.2025 | 27 | SIT | 200 | 209 | 46.93(4.25) | 3.97 | 4.21 | 1.13 | 14 | 8 | 19.8(2.67) | China | volleyball | Male |
| Wei et al.2025 | 27 | HIIT | 201 | 216 | 45.55(5.34) | 1.98 | 5.34 | 1.43 | 14 | 8 | 20.7(1.4) | China | volleyball | Male |
| Wells et al.2014 | 28 | CG | 0 | 0 | 57.1（3.6） | 0.50 | 3.38 | 1.19 | 8 | 8 | 21.3（2.1） | UK | soccer | Male |
| Wells et al.2014 | 28 | HIIT | 250 | 228 | 57.6（5.4） | 1.30 | 5.09 | 1.80 | 8 | 8 | 21.3（2.1） | UK | soccer | Male |
| Xu et al.2025 | 29 | RT | 200 | 198 | 41.9（2.7） | 0.20 | 2.65 | 0.94 | 8 | 8 | 21.4（2.2） | China | Taekwondo | Male |
| Xu et al.2025 | 29 | SIT | 350 | 346 | 42.3（2.8） | 2.30 | 2.95 | 1.04 | 8 | 8 | 21.7（2.4） | China | Taekwondo | Male |
| Xu et al.2025 | 29 | CG | 0 | 0 | 41.8(2.6) | 0.10 | 2.55 | 0.90 | 8 | 8 | 21.2(2.4) | China | Taekwondo | Male |
| Yang et al.2024 | 30 | RT | 200 | 200 | 50.1（2.2） | 3.10 | 2.36 | 0.75 | 10 | 6 | 21.2(1.4) | China | soccer | Male |
| Yang et al.2024 | 30 | SIT | 500 | 500 | 50.2（2.3） | 3.10 | 2.30 | 0.73 | 10 | 6 | 22.4(1.6) | China | soccer | Male |
| Yang et al.2024 | 30 | CG | 0 | 0 | 50.1（2.6） | 0.30 | 2.76 | 0.87 | 10 | 6 | 22.2(1.9) | China | soccer | Male |
| Yu et al.2025 | 31 | RT | 200 | 198 | 64.92（5.18） | 3.42 | 5.43 | 1.36 | 16 | 8 | 16.81(0.61) | China | endurance runners | Male |
| Yu et al.2025 | 31 | RT | 300 | 284 | 65.84（5.95） | 3.33 | 5.76 | 1.44 | 16 | 8 | 16.75(0.68) | China | endurance runners | Male |

**Note:** MET assignment rules for each modality are provided in Supplementary Table S13. The model dose column reports the MET-min/week value used in the dose-response network meta-analysis. Original dose refers to the extracted or pre-rounded dose value before recoding for model input. CG was assigned a dose of 0 because it represented maintenance of regular sport-specific training without additional supplementary exercise.

## **14.4 Connectivity**

Connectivity is a fundamental assumption in network meta-analysis (NMA). Insufficient connectivity, often resulting from limited direct comparisons, can reduce statistical power and bias results. In our study, connectivity was assessed across all dose and agent levels for each outcome (Figure S14-S15), with no evidence of network disconnection observed.

## **Figure S14.** Dose-level network

Line thickness represents the number of studies available for comparison. The first value represents the specific intervention, and the second value corresponds to the dose (METs-min/week).

**Figure S15.** Agent-level network

Line thickness represents the number of studies available for comparison.

## **14.5 Consistency**

We performed a consistency analysis of the data by comparing the consistency of the network i.e., network effect size with the unrelated mean effects UME model i.e., pairwise effect sizes. In practice, we checked whether the bias, the number of estimated parameters in the network, and the Deviance Information Criterion DIC metric were similar for both models, indicating a good fit. The comparison of these parameters showed a good agreement between the different models Table S14.

**Table S14.** Comparison of consistency and UME model fit.

| **Model** | **PD** | **Deviance** | **Residual deviance** | **DIC** | **SD** |
| --- | --- | --- | --- | --- | --- |
| Consistent | 47.2 | 67.106 | 234.838 | 281.5 | 1.011 |
| UME | 53.2 | 66.725 | 234.457 | 286.6 | 1.352 |

Note: UME = unrelated mean effects model; DIC = deviance information criterion; PD = effective number of parameters; SD = standard deviation

## **14.6 Consistency assessment**

Node-splitting was used to compare direct and indirect evidence where both sources were available. This analysis evaluates local inconsistency rather than transitivity. The node-splitting results for the dose-response network meta-analysis are presented in Table S15. The separate descriptive assessment of potential effect modifiers relevant to the transitivity assumption is provided in Supplementary Table S20.

**Table S15.** Node-splitting analysis of consistency.

========================================

|Comparison | p-value| Median| 2.5%| 97.5%|

|:----------------------|-------:|------:|------:|------:|

|SSG_500 vs SIT_450 | **0.608**| | | |

|-> direct | | -0.853| -3.726| 1.931|

|-> indirect | | 0.465| -1.785| 2.876|

|-> MBNMA | | -0.114| -1.804| 1.661|

| | | | | |

|SIT_50 vs RT_550 | **0.568**| | | |

|-> direct | | -0.906| -5.369| 3.635|

|-> indirect | | -0.950| -2.650| 0.663|

|-> MBNMA | | -0.741| -2.309| 0.779|

| | | | | |

|RT_300 vs RT_200 | **0.086**| | | |

|-> direct | | -0.114| -4.194| 3.938|

|-> indirect | | 0.037| -0.061| 0.211|

|-> MBNMA | | 0.036| -0.058| 0.200|

| | | | | |

|MICT_800 vs HIIT_450 | **0.562**| | | |

|-> direct | | -3.996| -9.281| 1.457|

|-> indirect | | -0.456| -6.701| 6.022|

|-> MBNMA | | -2.662| -6.678| 1.759|

| | | | | |

|SIT_200 vs HIIT_201 | **0.323**| | | |

|-> direct | | 1.961| -2.113| 5.779|

|-> indirect | | -0.230| -1.582| 1.099|

|-> MBNMA | | -0.371| -1.647| 0.904|

| | | | | |

|SIT_50 vs HIIT_200 | **0.576**| | | |

|-> direct | | -0.488| -2.891| 1.901|

|-> indirect | | 0.482| -1.041| 1.849|

|-> MBNMA | | 0.451| -0.887| 1.695|

| | | | | |

|HIIT_1000 vs AE_1200 | **0.115**| | | |

|-> direct | | 3.103| -3.737| 10.632|

|-> indirect | | -4.201| -6.131| -1.996|

|-> MBNMA | | -4.277| -6.159| -2.168|

| | | | | |

|HIIT_600 vs AE_1200 | **0.207**| | | |

|-> direct | | 1.816| -6.366| 9.431|

|-> indirect | | -4.166| -6.122| -2.115|

|-> MBNMA | | -4.142| -5.990| -2.036|

| | | | | |

|SIT_150 vs AE_700 | **0.102**| | | |

|-> direct | | 2.812| -3.264| 8.451|

|-> indirect | | -3.903| -6.196| -1.398|

|-> MBNMA | | -3.794| -5.833| -1.513|

| | | | | |

|HIIT_250 vs AE_550 | **0.066**| | | |

|-> direct | | 3.800| 1.598| 5.902|

|-> indirect | | -2.205| -6.411| 2.031|

|-> MBNMA | | -3.647| -5.305| -1.730|

| | | | | |

|SSG_350 vs Placebo_0 | **0.631**| | | |

|-> direct | | 1.695| -1.293| 4.433|

|-> indirect | | 2.825| 0.687| 5.132|

|-> MBNMA | | 2.322| 0.507| 4.299|

| | | | | |

|SSG_300 vs Placebo_0 | **0.387**| | | |

|-> direct | | 4.664| -0.404| 10.137|

|-> indirect | | 2.055| 0.261| 3.971|

|-> MBNMA | | 2.268| 0.496| 4.175|

| | | | | |

|SSG_100 vs Placebo_0 | **0.293**| | | |

|-> direct | | 3.756| 0.565| 6.981|

|-> indirect | | 1.280| -0.008| 2.934|

|-> MBNMA | | 1.691| 0.375| 3.529|

| | | | | |

|SIT_500 vs Placebo_0 | **0.519**| | | |

|-> direct | | 1.752| -0.634| 4.456|

|-> indirect | | 2.937| 1.548| 4.480|

|-> MBNMA | | 2.580| 1.462| 3.848|

| | | | | |

|SIT_400 vs Placebo_0 | **0.583**| | | |

|-> direct | | 2.615| -0.341| 5.698|

|-> indirect | | 2.523| 1.323| 3.855|

|-> MBNMA | | 2.505| 1.434| 3.706|

| | | | | |

|SIT_350 vs Placebo_0 | **0.607**| | | |

|-> direct | | 2.343| -0.577| 5.284|

|-> indirect | | 2.462| 1.269| 3.800|

|-> MBNMA | | 2.455| 1.393| 3.610|

| | | | | |

|SIT_250 vs Placebo_0 | **0.207**| | | |

|-> direct | | -0.156| -3.785| 3.593|

|-> indirect | | 2.523| 1.513| 3.623|

|-> MBNMA | | 2.311| 1.319| 3.379|

| | | | | |

|SIT_150 vs Placebo_0 | **0.519**| | | |

|-> direct | | 2.746| 0.579| 4.965|

|-> indirect | | 1.915| 0.963| 3.043|

|-> MBNMA | | 2.053| 1.132| 3.031|

| | | | | |

|SIT_130 vs Placebo_0 | **0.419**| | | |

|-> direct | | 2.454| -1.175| 6.218|

|-> indirect | | 1.947| 1.025| 2.935|

|-> MBNMA | | 1.966| 1.064| 2.959|

| | | | | |

|RT_750 vs Placebo_0 | **0.192**| | | |

|-> direct | | 4.510| -5.622| 16.387|

|-> indirect | | 0.662| -0.820| 2.072|

|-> MBNMA | | 0.667| -0.688| 2.105|

| | | | | |

|RT_700 vs Placebo_0 | **0.373**| | | |

|-> direct | | -0.049| -6.608| 6.606|

|-> indirect | | 0.685| -0.769| 2.261|

|-> MBNMA | | 0.664| -0.680| 2.086|

| | | | | |

|RT_400 vs Placebo_0 | **0.334**| | | |

|-> direct | | 2.493| -0.901| 5.985|

|-> indirect | | 0.368| -0.934| 1.728|

|-> MBNMA | | 0.623| -0.640| 1.978|

| | | | | |

|RT_200 vs Placebo_0 | **0.331**| | | |

|-> direct | | 1.561| -0.364| 3.527|

|-> indirect | | 0.001| -1.246| 1.436|

|-> MBNMA | | 0.536| -0.558| 1.742|

| | | | | |

|MICT_1000 vs Placebo_0 | **0.597**| | | |

|-> direct | | 1.586| -5.317| 8.593|

|-> indirect | | -1.784| -7.110| 4.054|

|-> MBNMA | | -0.431| -4.646| 4.223|

| | | | | |

|HIIT_1350 vs Placebo_0 | **0.291**| | | |

|-> direct | | 4.675| 1.034| 8.419|

|-> indirect | | 2.040| 0.564| 3.683|

|-> MBNMA | | 2.475| 1.051| 4.060|

| | | | | |

|HIIT_700 vs Placebo_0 | **0.313**| | | |

|-> direct | | 1.382| -7.353| 9.874|

|-> indirect | | 2.396| 0.984| 3.797|

|-> MBNMA | | 2.342| 0.992| 3.725|

| | | | | |

|HIIT_650 vs Placebo_0 | **0.623**| | | |

|-> direct | | 1.619| -1.340| 4.493|

|-> indirect | | 2.341| 0.993| 3.872|

|-> MBNMA | | 2.325| 0.983| 3.678|

| | | | | |

|HIIT_550 vs Placebo_0 | **0.362**| | | |

|-> direct | | 0.576| -3.275| 4.388|

|-> indirect | | 2.521| 1.086| 4.086|

|-> MBNMA | | 2.281| 0.955| 3.561|

| | | | | |

|HIIT_500 vs Placebo_0 | **0.532**| | | |

|-> direct | | 2.738| -1.076| 6.367|

|-> indirect | | 2.173| 0.805| 3.592|

|-> MBNMA | | 2.248| 0.942| 3.491|

| | | | | |

|HIIT_400 vs Placebo_0 | **0.397**| | | |

|-> direct | | 3.326| -1.224| 7.558|

|-> indirect | | 2.033| 0.824| 3.341|

|-> MBNMA | | 2.167| 0.914| 3.358|

| | | | | |

|HIIT_350 vs Placebo_0 | **0.389**| | | |

|-> direct | | 3.130| -1.946| 8.512|

|-> indirect | | 2.003| 0.842| 3.299|

|-> MBNMA | | 2.111| 0.901| 3.272|

| | | | | |

|HIIT_250 vs Placebo_0 | **0.336**| | | |

|-> direct | | 0.485| -3.829| 5.227|

|-> indirect | | 2.019| 0.912| 3.112|

|-> MBNMA | | 1.952| 0.842| 3.088|

| | | | | |

|HIIT_150 vs Placebo_0 | **0.352**| | | |

|-> direct | | 0.857| -4.030| 5.964|

|-> indirect | | 1.709| 0.756| 2.869|

|-> MBNMA | | 1.661| 0.721| 2.803|

| | | | | |

**Note:** The first value represents the specific intervention, and the second value corresponds to the dose (METs-min/week).

## **14.7 Model Selection**

For model selection, we adopted a quadratic function model. In accordance with the default settings in MBNMAdose, we applied vague (non-informative) prior distributions to minimize bias and allow the data to drive the results, thereby enabling the model to adapt flexibly to the underlying data structure. This approach is designed to capture a specific form of non-linear dose–response relationship and to identify an optimal dose under the assumption of a quadratic association. Below, we present a comparison of parameter estimates across different models (Table S16). In general, lower DIC indicate a better model fit. As the quadratic function model exhibited the lowest DIC value, we selected it for our analysis.

**Table S16.** Models fit comparison

| **Model** | **DIC** | **SD** | **Deviance** | **Residual deviance** | **pD** |
| --- | --- | --- | --- | --- | --- |
| Emax  (random treatment effects) | 272.9 | 0.730 | 229.671 | 61.939 | 43.7 |
| Restricted cubic spline (random treatment effects; 3 knots) | 284.2 | 0.616 | 231.008 | 63.276 | 52.9 |
| Log Linear  (random treatment effect) | 272.6 | 0.724 | 229.690 | 61.958 | 42.8 |
| Non-parametric monotonically up (random treatment effects) | 287.3 | 0.555 | 231.767 | 64.035 | 54.8 |
| Exponential dose-response function  (random treatment effects) | 288.3 | 0.320 | 238.934 | 71.202 | 50.3 |
| Quadratic function  (random treatment effects) | 272.1 | 1.048 | 234.929 | 67.197 | 47.1 |
| Spline dose-response functions  (random treatment effects) | 285.2 | 1.676 | 230.956 | 63.224 | 54.4 |
| Fractional polynomial dose-response function  (random treatment effects) | 293.2 | 0.497 | 237.338 | 69.606 | 55.6 |

**Note:** DIC: deviance information criterion; pD: effective number of parameters; SD: standard deviation.

## **14.8 Dose-Response Curves**

**Table S17.** Dose-response characteristics by training modality.

| Agent | Minimum Effective Dose | Characteristic |
| --- | --- | --- |
| SIT | Flat curve near zero | Highest efficiency; peak benefit at ~500 METs-min/week |
| SSG | Flat curve near zero | Moderate dose required for significant benefit |
| HIIT | Flat curve near zero | Linear positive dose-response to 1,400 METs-min/week |
| AE | Not significant at any dose | 95% CrI crosses zero across entire dose range |
| MICT | Not significant at any dose | 95% CrI crosses zero across entire dose range |
| RT | Not significant at any dose | Minimal effect on VO2max; targets muscular strength |

**Note:** SIT and HIIT demonstrated the most favorable dose-response profiles. The lack of significant dose-response for AE, MICT and RT likely reflects insufficient stimulus differentiation from athletes' regular training, rather than inefficacy. The doses represent training performed in addition to athletes' regular sport-specific routines.

**Figure 16.** The dose-response relationship between exercise and the increase in athletes' maximal oxygen uptake level.

The blue dashed lines represent the minimum and maximum effective doses, while the blue shaded area indicates the significant region. and the red dots represent the optimal dose points. METs-min/week: Metabolic Equivalents of Task minutes per week

**Table S18.** Summary of dose-response network meta-analysis result.

| Category | Effective dose (METs-min/week) | Optimal dose (METs-min/week) | Mean difference and 95% CI (optimal dose) |
| --- | --- | --- | --- |
| AE | NA | NA | NA |
| MICT | NA | NA | NA |
| RT | NA | NA | NA |
| HIIT | 0-1400 | 1400 | 4.43 mL/kg/min (0.31, 8.83) |
| SIT | 0-500 | 500 | 2.99 mL/kg/min (1.10, 4.98) |
| SSG | 0-170 | 170 | 1.01 mL/kg/min (0.01, 2.02) |

**15. Additional Transitivity Assessment**

**Table S20.** Descriptive assessment of potential effect modifiers across treatment nodes.

This table summarizes study-arm characteristics from the main network meta-analysis to support the descriptive assessment of transitivity. Values are summarized at the treatment-node level. Sex distribution is reported by study arm because participant-level sex counts were not consistently available across trials.

| **Treatment node** | **No. of studies (arms)** | **Main sport types represented** | **Age range, years** | **Baseline VO2max range, mL/kg/min** | **Sex distribution, arms** | **Intervention duration, weeks** | **Competitive level** | **Direct CG comparisons** | **Transitivity concern** |
| --- | --- | --- | --- | --- | --- | --- | --- | --- | --- |
| AE | 18 (18) | rowing, soccer, cycling, running, etc. | 17.5-47.2 | 47.6-70.8 | male=8; mixed=1; NR=9 | 4-12 | Not consistently reported | Yes (k=1) | overlap with regular aerobic training. |
| BFR | 3 (3) | basketball, rowing, cycling | 19.7-39.6 | 39.5-63.0 | male=2; NR=1 | 5-12 | Not consistently reported | No direct CG comparison | no direct CG comparison; limited sport coverage. |
| HIIT | 30 (32) | basketball, volleyball, rowing, soccer, etc. | 13.5-47.2 | 36.7-81.0 | male=18; mixed=4; NR=10 | 4-16 | Not consistently reported | Yes (k=12) | heterogeneity across sports and baseline fitness. |
| HVT | 2 (2) | soccer, endurance athletes | 13.5-31.0 | 55.3-60.5 | male=1; NR=1 | 5-9 | Not consistently reported | No direct CG comparison | sparse evidence; no direct CG comparison; limited sport coverage. |
| IHT | 10 (12) | triathlon, swimming, cycling, running | 19.1-30.3 | 56.0-73.5 | male=9; NR=3 | 4-7 | Not consistently reported | Yes (k=1) | normoxic/hypoxic contexts pooled. |
| MICT | 5 (6) | endurance athletes, cycling, rowing, running, etc. | 15.3-39.2 | 46.2-80.0 | male=2; NR=4 | 5-10 | Not consistently reported | Yes (k=1) | overlap with regular aerobic training. |
| POL | 2 (2) | ultra-endurance running, endurance athletes | 31.0-40.6 | 55.8-60.6 | male=1; NR=1 | 9-12 | Not consistently reported | No direct CG comparison | sparse evidence; no direct CG comparison; limited sport coverage. |
| RT | 8 (8) | cycling, volleyball, triathlon, karate, etc. | 19.5-29.6 | 37.9-73.5 | male=6; NR=2 | 6-25 | Not consistently reported | Yes (k=7) | heterogeneity across sports and baseline fitness. |
| SIT | 16 (17) | volleyball, running, football, judo, etc. | 13.6-29.6 | 41.9-67.2 | male=13; NR=4 | 4-8 | Not consistently reported | Yes (k=10) | heterogeneity across sports and baseline fitness. |
| SSG | 4 (4) | basketball, tennis, soccer | 13.8-26.2 | 45.9-57.4 | male=3; NR=1 | 6-8 | Not consistently reported | Yes (k=1) | concentrated in team-ball contexts. |
| THR | 2 (2) | ultra-endurance running, endurance athletes | 31.0-36.8 | 57.1-63.2 | male=1; NR=1 | 9-12 | Not consistently reported | No direct CG comparison | sparse evidence; no direct CG comparison; limited sport coverage. |
| CG | 28 (28) | cycling, volleyball, triathlon, rowing, etc. | 16.0-47.2 | 35.2-77.0 | male=17; mixed=2; NR=9 | 4-25 | Not consistently reported | Reference node | Active sport-specific training; background load varied across sports. |

Abbreviations: AE, aerobic exercise; BFR, blood flow restriction training; CG, control group; HIIT, high-intensity interval training; HVT, high-volume training; IHT, intermittent hypoxic training; MICT, moderate-intensity continuous training; POL, polarized training; RT, resistance training; SIT, sprint interval training; SSG, small-sided games; THR, threshold training; VO?max, maximal oxygen uptake. Direct CG comparisons indicate studies in which the treatment node was compared with a control arm maintaining regular sport-specific training.

**References**

1.	Aagaard P, Andersen JL, Bennekou M, et al. Effects of resistance training on endurance capacity and muscle fiber composition in young top-level cyclists. *Scand J Med Sci Sports*. 2011;21(6):1-10. doi:10.1111/j.1600-0838.2010.01283.x

2.	Arslan E, Kilit B, Clemente FM, et al. Effects of small-sided games training versus high-intensity interval training approaches in young basketball players. *Int J Environ Res Public Health*. 2022;19(5):2931. doi:10.3390/ijerph19052931

3.	Biçer M. The effect of an eight-week strength training program supported with functional sports equipment on Male volleyball players’ anaerobic and aerobic power. *Sci Sports*. 2021;36(2):137.e1-137.e9. doi:10.1016/j.scispo.2020.02.006

4.	Bilici ÖF, Topateş TK. Comparative effects of high intensity interval and functional training on performance outcomes in adolescent female volleyball players. *BMC Sports Sci Med Rehabil*. 2025;18(1):34-53. doi:10.1186/s13102-025-01476-w

5.	Ramos-Campo D, Martínez-Sánchez F, Esteban-García P, Rubio-Arias J, Clemente-Suarez V, Jiménez-Díaz J. The effects of intermittent hypoxia training on hematological and aerobic performance in triathletes. *Acta Physiol Hung*. 2015;102(4):409-418. doi:10.1556/036.102.2015.4.8

6.	Ní Chéilleachair NJ, Harrison AJ, Warrington GD. HIIT enhances endurance performance and aerobic characteristics more than high-volume training in trained rowers. *J Sports Sci*. 2017;35(11):1052-1058. doi:10.1080/02640414.2016.1209539

7.	Chen L, Zhang Z, Qu W, et al. Effects of blood flow restriction moderate intensity interval training on aerobic and anaerobic capabilities and lower extremity performance in Male college basketball players. *BMC Sports Sci Med Rehabil*. 2025;17(1):44-55. doi:10.1186/s13102-025-01100-x

8.	Clark JE. The use of an 8-week mixed-intensity interval endurance-training program improves the aerobic fitness of female soccer players. *J Strength Cond Res*. 2010;24(7):1773-1781. doi:10.1519/JSC.0b013e3181ce2495

9.	Czuba M, Wilk R, Karpiński J, Chalimoniuk M, Zajac A, Langfort J. Intermittent hypoxic training improves anaerobic performance in competitive swimmers when implemented into a direct competition mesocycle. Toborek M, ed. *PLOS One*. 2017;12(8):e0180380. doi:10.1371/journal.pone.0180380

10.	M C, O FC, K P, A Z, J L. Comparison of the effect of intermittent hypoxic training vs. the live high, train low strategy on aerobic capacity and sports performance in cyclists in normoxia. *Biol Sport*. Published online 2017:1-10. doi:10.5114/biolsport.2018.70750

11.	Driller MW, Fell JW, Gregory JR, Shing CM, Williams AD. The effects of high-intensity interval training in well-trained rowers. *Int J Sports Physiol Perform*. 2009;4(1):111-121. doi:10.1123/ijspp.4.1.110

12.	Dufour SP, Ponsot E, Zoll J, et al. Exercise training in normobaric hypoxia in endurance runners. I. Improvement in aerobic performance capacity. *J Appl Physiol*. 2006;100(4):1238-1248. doi:10.1152/japplphysiol.00742.2005

13.	Fernandez-Fernandez J, Zimek R, Wiewelhove T, Ferrauti A. High-intensity interval training vs. Repeated-sprint training in tennis. *J Strength Cond Res*. 2012;26(1):53-62. doi:10.1519/JSC.0b013e318220b4ff

14.	Short-term effects of repeated-sprint training on vertical jump ability and aerobic fitness in collegiate volleyball players during pre-season. *Int J Exerc Sci*. 2022;15(6):1-12. doi:10.70252/UXOP1748

15.	Held S, Behringer M, Donath L. Low intensity rowing with blood flow restriction over 5 weeks increases V̇O2max in elite rowers: a randomized controlled trial. *J Sci Med Sport*. 2020;23(3):304-308. doi:10.1016/j.jsams.2019.10.002

16.	Jarstad E, Mamen A. The performance and aerobic endurance effects of high-intensity versus moderate-intensity continuous running. *Appl Physiol Nutr Metab*. 2019;44(9):991-996. doi:10.1139/apnm-2018-0575

17.	Javaloyes A, Sarabia JM, Lamberts RP, Moya-Ramon M. Training prescription guided by heart-rate variability in cycling. *Int J Sports Physiol Perform*. 2019;14(1):23-32. doi:10.1123/ijspp.2018-0122

18.	Jin K, Cai M, Zhang Y, Wu B, Yang Y. Effects of 6-week sprint interval training compared to traditional training on the running performance of distance runners: a randomized controlled trail. *Front Physiol*. 2025;16:1536287. doi:10.3389/fphys.2025.1536287

19.	Jin R, Sun J, Jiang N, Chen C. Influence of 4-week lower extremity high-intensity interval training on energy metabolism and maximal oxygen uptake of elite swimmers. *PeerJ*. 2025;13:e19788. doi:10.7717/peerj.19788

20.	Kaynak K, Eryılmaz SK, Aydoğan S, Mihailov D. The effects of 20-m repeated sprint training on aerobic capacity in college volleyball players. *Biomed Hum Kinet*. 2017;9(1):43-50. doi:10.1515/bhk-2017-0007

21.	Kelly DT, Cregg CJ, O’Connor PL, Cullen BD, Moyna NM. Physiological and performance responses of sprint interval training and endurance training in gaelic football players. *Eur J Appl Physiol*. 2021;121(8):2265-2275. doi:10.1007/s00421-021-04699-0

22.	Kilit B, Arslan E. Effects of high-intensity interval training vs. On-court tennis training in young tennis players. *J Strength Cond Res*. 2019;33(1):188-196. doi:10.1519/JSC.0000000000002766

23.	Kim J, Lee N, Trilk J, et al. Effects of sprint interval training on elite judoists. *Int J Sports Med*. 2011;32(12):929-934. doi:10.1055/s-0031-1283183

24.	Kirchenberger T, Ketelhut S, Ketelhut RG. Effects of moderate- versus mixed-intensity training on VO2peak in young well-trained rowers. *Sports*. 2021;9(7):92-100. doi:10.3390/sports9070092

25.	Kristoffersen M, Sandbakk Ø, Rønnestad BR, Gundersen H. Comparison of short-sprint and heavy strength training on cycling performance. *Front Physiol*. 2019;10:1132. doi:10.3389/fphys.2019.01132

26.	Latino F, Susanto N, Anam K, Setyawan H, Saraiello E, Tafuri F. The effects of circuit training versus high-intensity interval training on the endurance of volleyball athletes: a randomized controlled trial. *Retos*. 2024;58(58):1050-1060. doi:10.47197/retos.v58.107877

27.	Liu H, Leng B, Li Q, Liu Y, Bao D, Cui Y. The effect of eight-week sprint interval training on aerobic performance of elite badminton players. *Int J Environ Res Public Health*. 2021;18(2):638-648. doi:10.3390/ijerph18020638

28.	Liu H, Wang J. The effects of incorporating dry-land short intervals to long aerobic-dominant in-water swimming training on physiological parameters, hormonal factors, and performance: a randomized-controlled intervention study. *J Sports Sci Med*. Published online June 1, 2023:329-337. doi:10.52082/jssm.2023.329

29.	Maggioni MA, Bonato M, Stahn A, et al. Effects of ball drills and repeated-sprint-ability training in basketball players. *Int J Sports Physiol Perform*. 2019;14(6):757-764. doi:10.1123/ijspp.2018-0433

30.	Mallol M, Bentley DJ, Norton L, Norton K, Mejuto G, Yanci J. Comparison of Reduced-Volume High-Intensity Interval Training and High-Volume Training on Endurance Performance in Triathletes. *Int J Sports Physiol Perform*. 2019;14(2):239-245. doi:10.1123/ijspp.2018-0359

31.	Meckel Y, Gefen Y, Nemet D, Eliakim A. Influence of short vs. Long repetition sprint training on selected fitness components in young soccer players. *J Strength Cond Res*. 2012;26(7):1845-1851. doi:10.1519/JSC.0b013e318236d0f0

32.	Millet GP, Jaouen B, Borrani F, Candau R. Effects of concurrent endurance and strength training on running economy and V˙ O2 kinetics. 1-9.

33.	Monks L, Seo MW, Kim HB, Jung HC, Song JK. High-intensity interval training and athletic performance in taekwondo athletes. *J Sports Med Phys Fitness*. 2017;57(10):1-9. doi:10.23736/S0022-4707.17.06853-0

34.	Morais J, Kilit B, Arslan E, Bragada J, Soylu Y, Marinho D. Effects of on-court tennis training combined with HIIT versus RST on aerobic capacity, speed, agility, jumping ability, and internal loads in young tennis players. *J Hum Kinet*. Published online December 6, 2024:1-13. doi:10.5114/jhk/189691

35.	Quezada-Muñoz Y, Rodríguez-Artigas P, Aravena-Sagardia P, et al. Effects of a high-intensity interval training program on body composition and physical fitness in female field hockey players. *Int J Morphol*. 2021;39(5):1323-1330. doi:10.4067/S0717-95022021000501323

36.	Ouergui I, Messaoudi H, Chtourou H, et al. Repeated sprint training vs. Repeated high-intensity technique training in adolescent taekwondo athletes—a randomized controlled trial. *Int J Environ Res Public Health*. 2020;17(12):4506. doi:10.3390/ijerph17124506

37.	Øvretveit K. Aerobic interval training improves maximal oxygen uptake and reduces body fat in grapplers. *J Sports Med Phys Fitness*. 2020;59(12):1-13. doi:10.23736/S0022-4707.19.09584-7

38.	Pérez A, Ramos-Campo DJ, Freitas TT, Rubio-Arias JÁ, Marín-Cascales E, Alcaraz PE. Effect of two different intensity distribution training programmes on aerobic and body composition variables in ultra-endurance runners. *Eur J Sport Sci*. 2019;19(5):636-644. doi:10.1080/17461391.2018.1539124

39.	Ponsot E, Dufour SP, Zoll J, et al. Exercise training in normobaric hypoxia in endurance runners. II. Improvement of mitochondrial properties in skeletal muscle. *J Appl Physiol*. 2006;100(4):1249-1257. doi:10.1152/japplphysiol.00361.2005

40.	Carrasco-Poyatos M, González-Quílez A, Altini M, Granero-Gallegos A. Heart rate variability-guided training in professional runners: effects on performance and vagal modulation. *Physiol Behav*. 2022;244:113654. doi:10.1016/j.physbeh.2021.113654

41.	Pugliese L, Porcelli S, Vezzoli A, et al. Different training modalities improve energy cost and performance in master runners. *Front Physiol*. 2018;9:21-29. doi:10.3389/fphys.2018.00021

42.	Robach P, Siebenmann C, Jacobs RA, et al. The role of haemoglobin mass on VO_2_ max following normobaric ‘live high–train low’ in endurance-trained athletes. *Br J Sports Med*. 2012;46(11):822-827. doi:10.1136/bjsports-2012-091078

43.	Rodríguez FA, Truijens MJ, Townsend NE, Stray-Gundersen J, Gore CJ, Levine BD. Performance of runners and swimmers after four weeks of intermittent hypobaric hypoxic exposure plus sea level training. *J Appl Physiol*. 2007;103(5):1523-1535. doi:10.1152/japplphysiol.01320.2006

44.	Roels B, Millet GP, Marcoux CJL, Coste O, Bentley DJ, Candau RB. Effects of hypoxic interval training on cycling performance: *Med Sci Sports Exercise*. 2005;37(1):138-146. doi:10.1249/01.MSS.0000150077.30672.88

45.	Rønnestad BR, Hansen EA, Raastad T. In-season strength maintenance training increases well-trained cyclists’ performance. *Eur J Appl Physiol*. 2010;110(6):1269-1282. doi:10.1007/s00421-010-1622-4

46.	Rønnestad BR, Hansen EA, Raastad T. Effect of heavy strength training on thigh muscle cross-sectional area, performance determinants, and performance in well-trained cyclists. *Eur J Appl Physiol*. 2010;108(5):965-975. doi:10.1007/s00421-009-1307-z

47.	Sandbakk Ø, Sandbakk SB, Ettema G, Welde B. Effects of intensity and duration in aerobic high-intensity interval training in highly trained junior cross-country skiers. *J Strength Cond Res*. 2013;27(7):1974-1980. doi:10.1519/JSC.0b013e3182752f08

48.	Sheykhlouvand M, Gharaat M. Optimal homeostatic stress to maximize the homogeneity of adaptations to interval interventions in soccer players. *Front Physiol*. 2024;15:1377552. doi:10.3389/fphys.2024.1377552

49.	Song Y, Sheykhlouvand M. A comparative analysis of high-intensity technique-specific intervals and short sprint interval training in taekwondo athletes: effects on cardiorespiratory fitness and anaerobic power. *J Sports Sci Med*. Published online September 1, 2024:672-683. doi:10.52082/jssm.2024.672

50.	Sperlich B, De Marées M, Koehler K, Linville J, Holmberg HC, Mester J. Effects of 5 weeks of high-intensity interval training vs. Volume training in 14-year-old soccer players. *J Strength Cond Res*. 2011;25(5):1271-1278. doi:10.1519/JSC.0b013e3181d67c38

51.	Stöggl T, Sperlich B. Polarized training has greater impact on key endurance variables than threshold, high intensity, or high volume training. *Front Physiol*. 2014;5:1-9. doi:10.3389/fphys.2014.00033

52.	Talsnes RK, Van Den Tillaar R, Sandbakk Ø. Effects of increased load of low-versus high-intensity endurance training on performance and physiological adaptations in endurance athletes. *International journal of sports physiology and performance*. 2021;17(2):216-225. doi:10.1123/ijspp.2021-0190

53.	Tangchaisuriya P, Chuensiri N, Tanaka H, Suksom D. Physiological adaptations to high-intensity interval training combined with blood flow restriction in masters road cyclists. *Med Sci Sports Exercise*. 2022;54(5):831-840. doi:10.1249/MSS.0000000000002857

54.	Ventura N, Hoppeler H, Seiler R, Binggeli A, Mullis P, Vogt M. The response of trained athletes to six weeks of endurance training in hypoxia or normoxia. *Int J Sports Med*. 2003;24(3):166-172. doi:10.1055/s-2003-39086

55.	Wang L, Wang J. Effects of high-intensity training on endurance in basketball players. *Rev Bras Med Esporte*. 2023;29:e2022_0633. doi:10.1590/1517-8692202329012022_0633

56.	Wei C, An J, Zhou L. The effects of 8 weeks of sprint interval training on repeated sprinting and specialized ability in college volleyball players. Akaras E, ed. *PLOS One*. 2025;20(7):e0327561. doi:10.1371/journal.pone.0327561

57.	Wells C, Edwards A, Fysh M, Drust B. Effects of high-intensity running training on soccer-specific fitness in professional male players. *Appl Physiol Nutr Metab*. 2014;39(7):763-769. doi:10.1139/apnm-2013-0199

58.	Xu Y, Wang S. Sequencing effects of concurrent resistance and short sprint interval training on physical fitness, and aerobic and anaerobic performance of karate athletes. *J Sports Sci Med*. Published online March 1, 2025:205-216. doi:10.52082/jssm.2025.205

59.	Yang G, Chen W, Qi D, Zhang J, Men Z. The effects of a 6-week plyometric and sprint interval training intervention on soccer player’s physical performance. *J Sports Sci Med*. Published online September 1, 2024:526-536. doi:10.52082/jssm.2024.526

60.	Zoll J, Ponsot E, Dufour S, et al. Exercise training in normobaric hypoxia in endurance runners. III. Muscular adjustments of selected gene transcripts. *J Appl Physiol*. 2006;100(4):1258-1266. doi:10.1152/japplphysiol.00359.2005

61.	Mallol et al.2019.

62.	Yu S, Zhou S, Peng D, Jie D. Effect of complex training on lower limb strength and running economy in adolescent distance runners. *Front Physiol*. 2025;16:1718150. doi:10.3389/fphys.2025.1718150

63.	Ainsworth BE, Haskell WL, Whitt MC, et al. Compendium of physical activities: an update of activity codes and MET intensities. *Med Sci Sports Exerc*. 2000;32(9 Suppl):S498-504. doi:10.1097/00005768-200009001-00009

64.	Gibala MJ, Little JP, Macdonald MJ, Hawley JA. Physiological adaptations to low-volume, high-intensity interval training in health and disease. *J Physiol*. 2012;590(5):1077-1084. doi:10.1113/jphysiol.2011.224725

65.	Buchheit M, Laursen PB. High-intensity interval training, solutions to the programming puzzle: Part I: cardiopulmonary emphasis. *Sports Med*. 2013;43(5):313-338. doi:10.1007/s40279-013-0029-x

66.	Seiler S. What is best practice for training intensity and duration distribution in endurance athletes? *Int J Sports Physiol Perform*. 2010;5(3):276-291. doi:10.1123/ijspp.5.3.276

67.	Garber CE, Blissmer B, Deschenes MR, et al. Quantity and Quality of Exercise for Developing and Maintaining Cardiorespiratory, Musculoskeletal, and Neuromotor Fitness in Apparently Healthy Adults: Guidance for Prescribing Exercise. *Med Sci Sports Exerc*. 2011;43(7):1334-1359. doi:10.1249/MSS.0b013e318213fefb

68.	Hill-Haas SV, Dawson B, Impellizzeri FM, Coutts AJ. Physiology of Small-Sided Games Training in Football: A Systematic Review. *Sports Medicine*. 2011;41(3):199-220. doi:10.2165/11539740-000000000-00000

69.	Progression Models in Resistance Training for Healthy Adults. *Medicine & Science in Sports & Exercise*. 2009;41(3):687-708. doi:10.1249/MSS.0b013e3181915670
